# Supplementary figures and images for: APE1 recruits ATRIP to ssDNA in an RPA-dependent and -independent manner to promote the ATR DNA damage response (part 1 of 4)
Source: eLife. 2023 May 22;12:e82324. doi: 10.7554/eLife.82324 (PMC10202453; doi:10.7554/eLife.82324)

Figure 1A

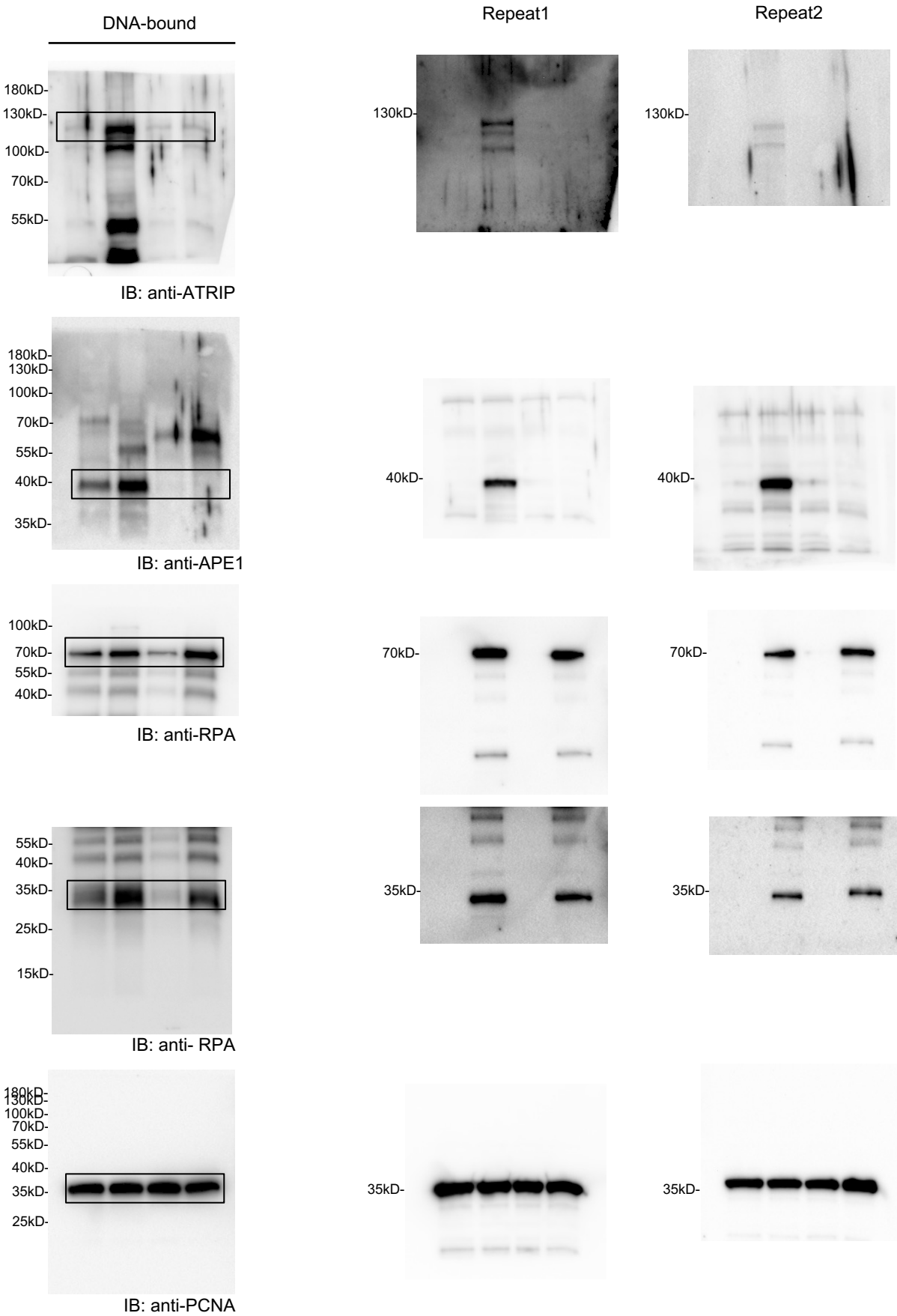

Figure 1A

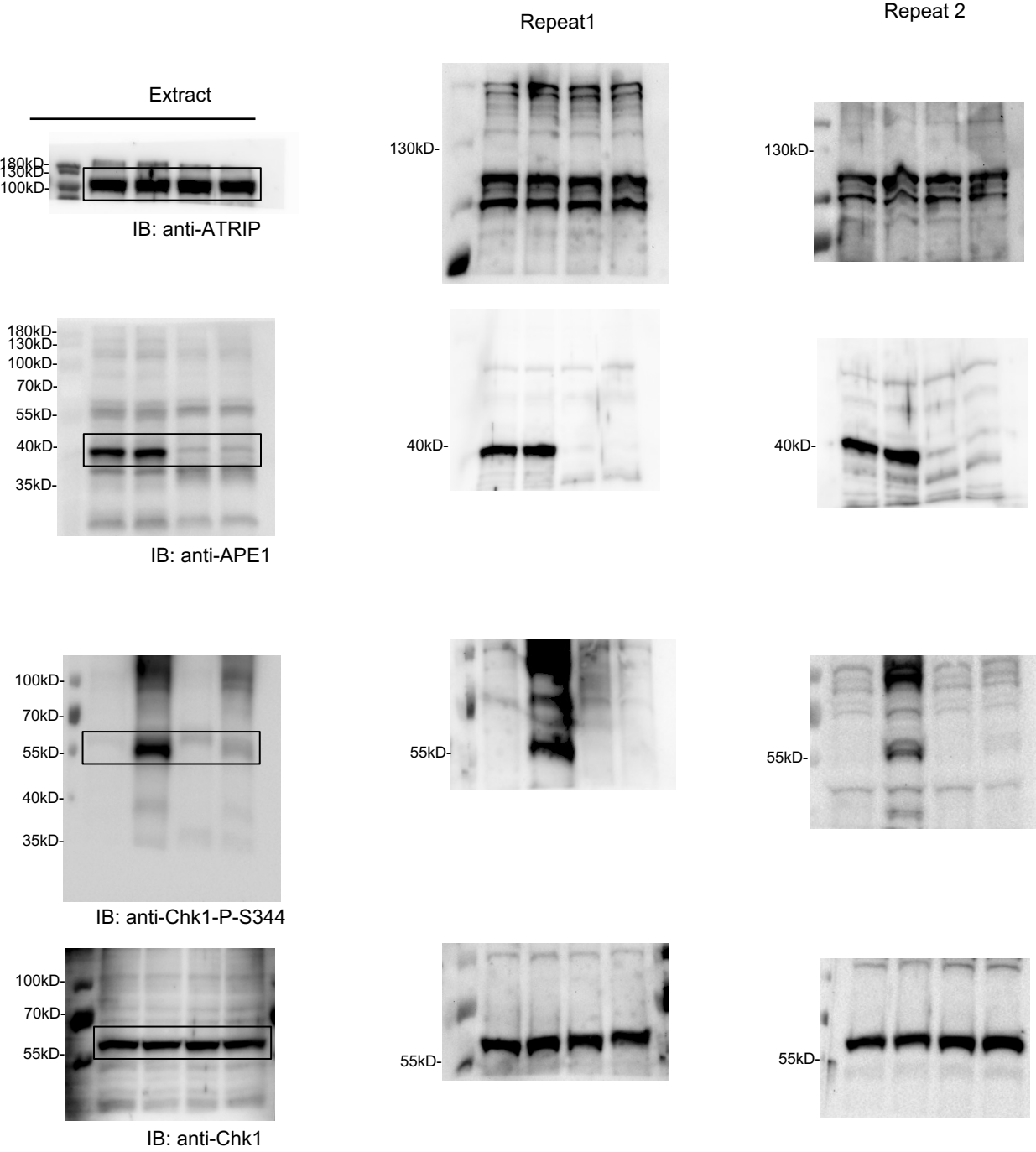

Supplement: Figure 1—source data 1. [file elife-82324-fig1-data1.zip › Figure 1-source data 1/IB-data-Figure 1A.pdf]

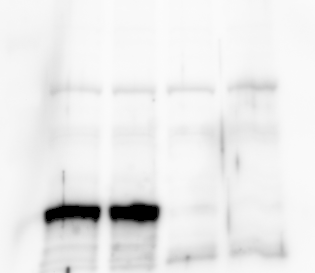

Supplement: Figure 1—source data 1. [file elife-82324-fig1-data1.zip › Figure 1-source data 1/Figure 1A Repeat1/Extract-APE1.tif]

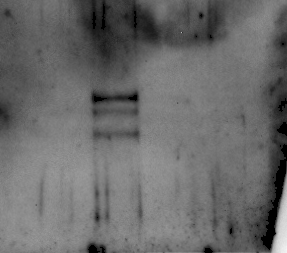

Supplement: Figure 1—source data 1. [file elife-82324-fig1-data1.zip › Figure 1-source data 1/Figure 1A Repeat1/DNA-bound-ATRIP.tif]

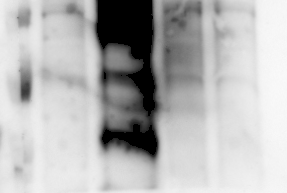

Supplement: Figure 1—source data 1. [file elife-82324-fig1-data1.zip › Figure 1-source data 1/Figure 1A Repeat1/Extract-Chk1-P-S344.tif]

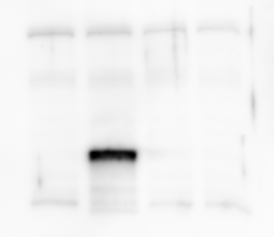

Supplement: Figure 1—source data 1. [file elife-82324-fig1-data1.zip › Figure 1-source data 1/Figure 1A Repeat1/DNA-bound-APE1.tif]

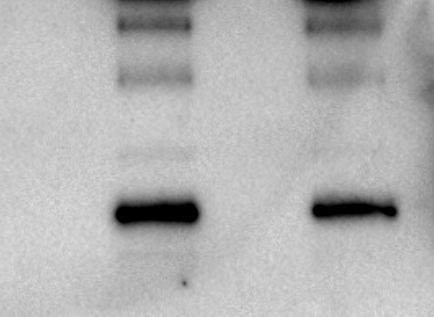

Supplement: Figure 1—source data 1. [file elife-82324-fig1-data1.zip › Figure 1-source data 1/Figure 1A Repeat1/DNA-bound-RPA32.tif]

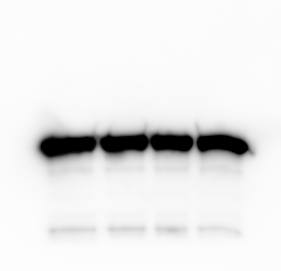

Supplement: Figure 1—source data 1. [file elife-82324-fig1-data1.zip › Figure 1-source data 1/Figure 1A Repeat1/DNA-bound-PCNA.tif]

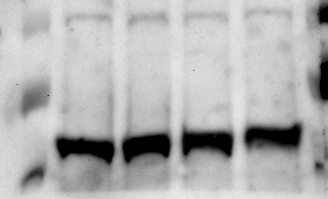

Supplement: Figure 1—source data 1. [file elife-82324-fig1-data1.zip › Figure 1-source data 1/Figure 1A Repeat1/Extract-Chk1.tif]

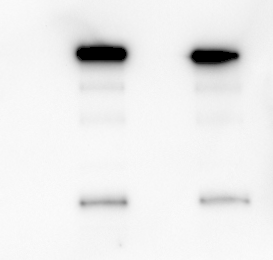

Supplement: Figure 1—source data 1. [file elife-82324-fig1-data1.zip › Figure 1-source data 1/Figure 1A Repeat1/DNA-bound-RPA70.tif]

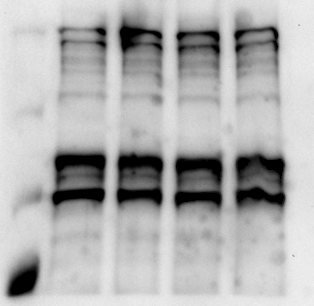

Supplement: Figure 1—source data 1. [file elife-82324-fig1-data1.zip › Figure 1-source data 1/Figure 1A Repeat1/Extract-ATRIP.tif]

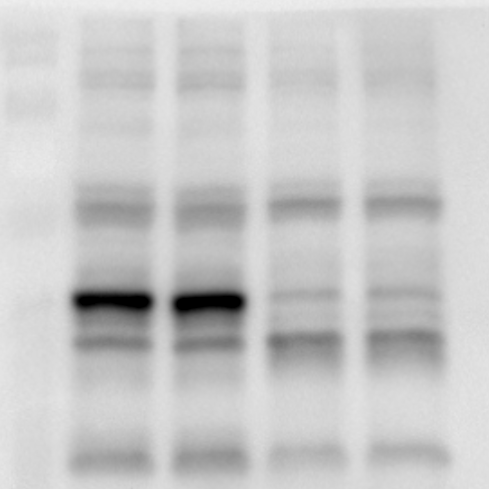

Supplement: Figure 1—source data 1. [file elife-82324-fig1-data1.zip › Figure 1-source data 1/Figure 1A initial trial/Extract-APE1.tif]

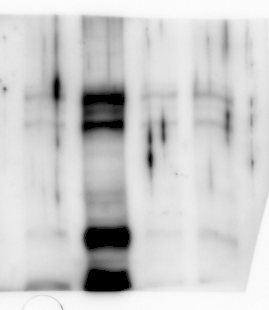

Supplement: Figure 1—source data 1. [file elife-82324-fig1-data1.zip › Figure 1-source data 1/Figure 1A initial trial/DNA-bound-ATRIP.tif]

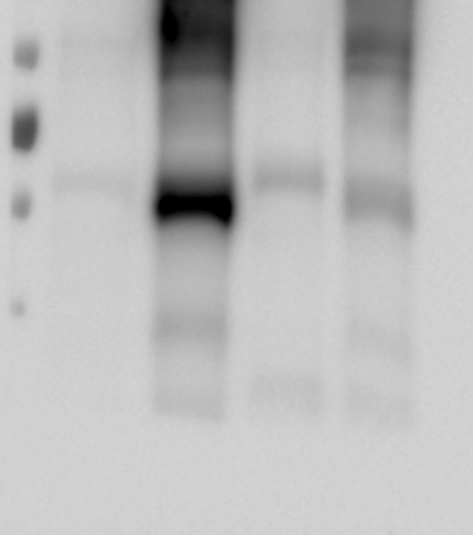

Supplement: Figure 1—source data 1. [file elife-82324-fig1-data1.zip › Figure 1-source data 1/Figure 1A initial trial/Extract-Chk1-P-S344.tif]

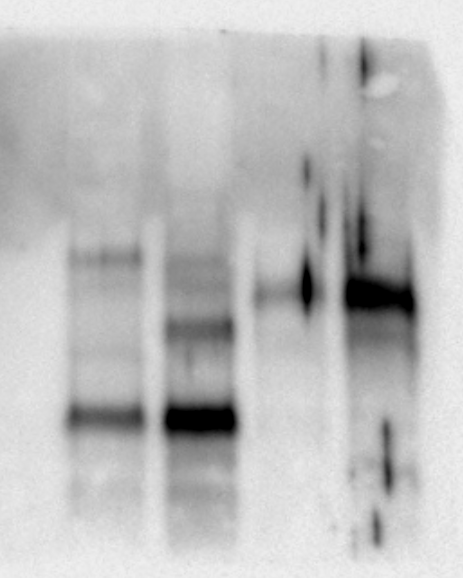

Supplement: Figure 1—source data 1. [file elife-82324-fig1-data1.zip › Figure 1-source data 1/Figure 1A initial trial/DNA-bound-APE1.tif]

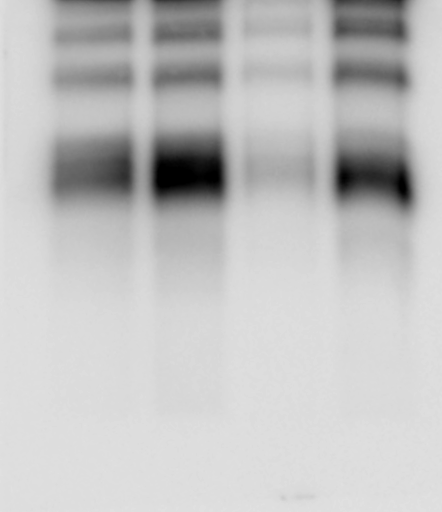

Supplement: Figure 1—source data 1. [file elife-82324-fig1-data1.zip › Figure 1-source data 1/Figure 1A initial trial/DNA-bound-RPA32.tif]

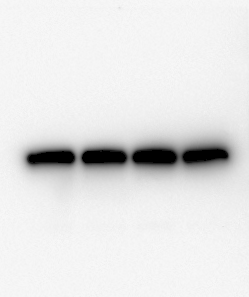

Supplement: Figure 1—source data 1. [file elife-82324-fig1-data1.zip › Figure 1-source data 1/Figure 1A initial trial/DNA-bound-PCNA.tif]

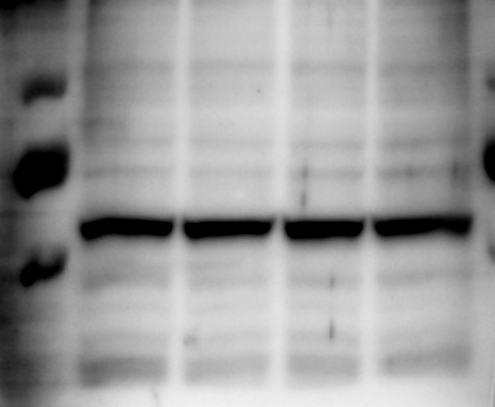

Supplement: Figure 1—source data 1. [file elife-82324-fig1-data1.zip › Figure 1-source data 1/Figure 1A initial trial/Extract-Chk1.tif]

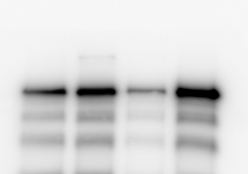

Supplement: Figure 1—source data 1. [file elife-82324-fig1-data1.zip › Figure 1-source data 1/Figure 1A initial trial/DNA-bound-RPA70.tif]

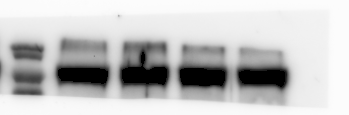

Supplement: Figure 1—source data 1. [file elife-82324-fig1-data1.zip › Figure 1-source data 1/Figure 1A initial trial/Extract-ATRIP.tif]

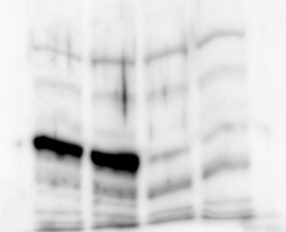

Supplement: Figure 1—source data 1. [file elife-82324-fig1-data1.zip › Figure 1-source data 1/Figure 1A Repeat2/Extract-APE1.tif]

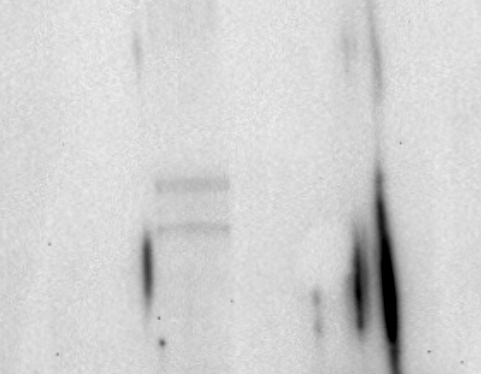

Supplement: Figure 1—source data 1. [file elife-82324-fig1-data1.zip › Figure 1-source data 1/Figure 1A Repeat2/DNA-bound-ATRIP.tif]

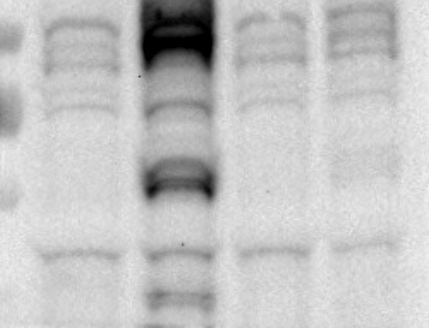

Supplement: Figure 1—source data 1. [file elife-82324-fig1-data1.zip › Figure 1-source data 1/Figure 1A Repeat2/Extract-Chk1-P-S344.tif]

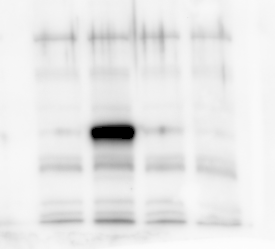

Supplement: Figure 1—source data 1. [file elife-82324-fig1-data1.zip › Figure 1-source data 1/Figure 1A Repeat2/DNA-bound-APE1.tif]

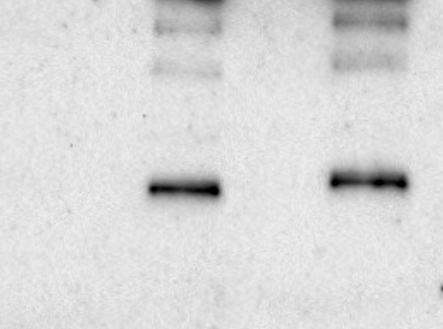

Supplement: Figure 1—source data 1. [file elife-82324-fig1-data1.zip › Figure 1-source data 1/Figure 1A Repeat2/DNA-bound-RPA32.tif]

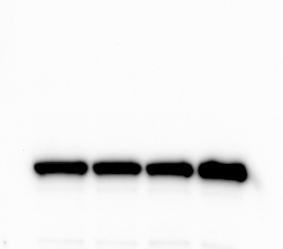

Supplement: Figure 1—source data 1. [file elife-82324-fig1-data1.zip › Figure 1-source data 1/Figure 1A Repeat2/DNA-bound-PCNA.tif]

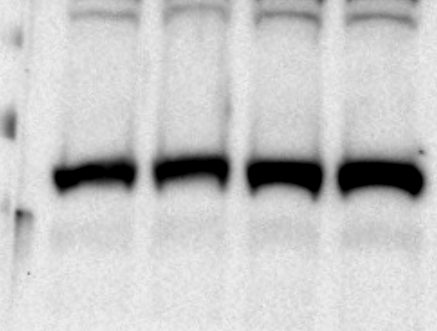

Supplement: Figure 1—source data 1. [file elife-82324-fig1-data1.zip › Figure 1-source data 1/Figure 1A Repeat2/Extract-Chk1.tif]

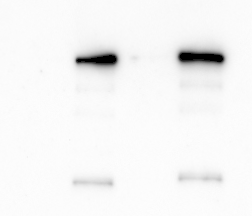

Supplement: Figure 1—source data 1. [file elife-82324-fig1-data1.zip › Figure 1-source data 1/Figure 1A Repeat2/DNA-bound-RPA70.tif]

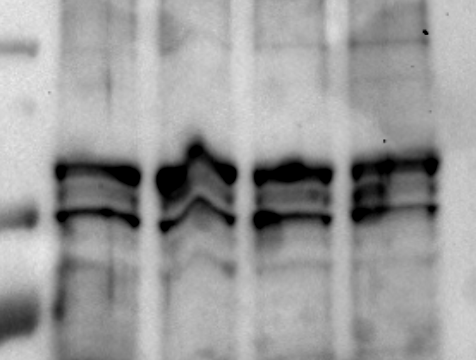

Supplement: Figure 1—source data 1. [file elife-82324-fig1-data1.zip › Figure 1-source data 1/Figure 1A Repeat2/Extract-ATRIP.tif]

Figure 1B

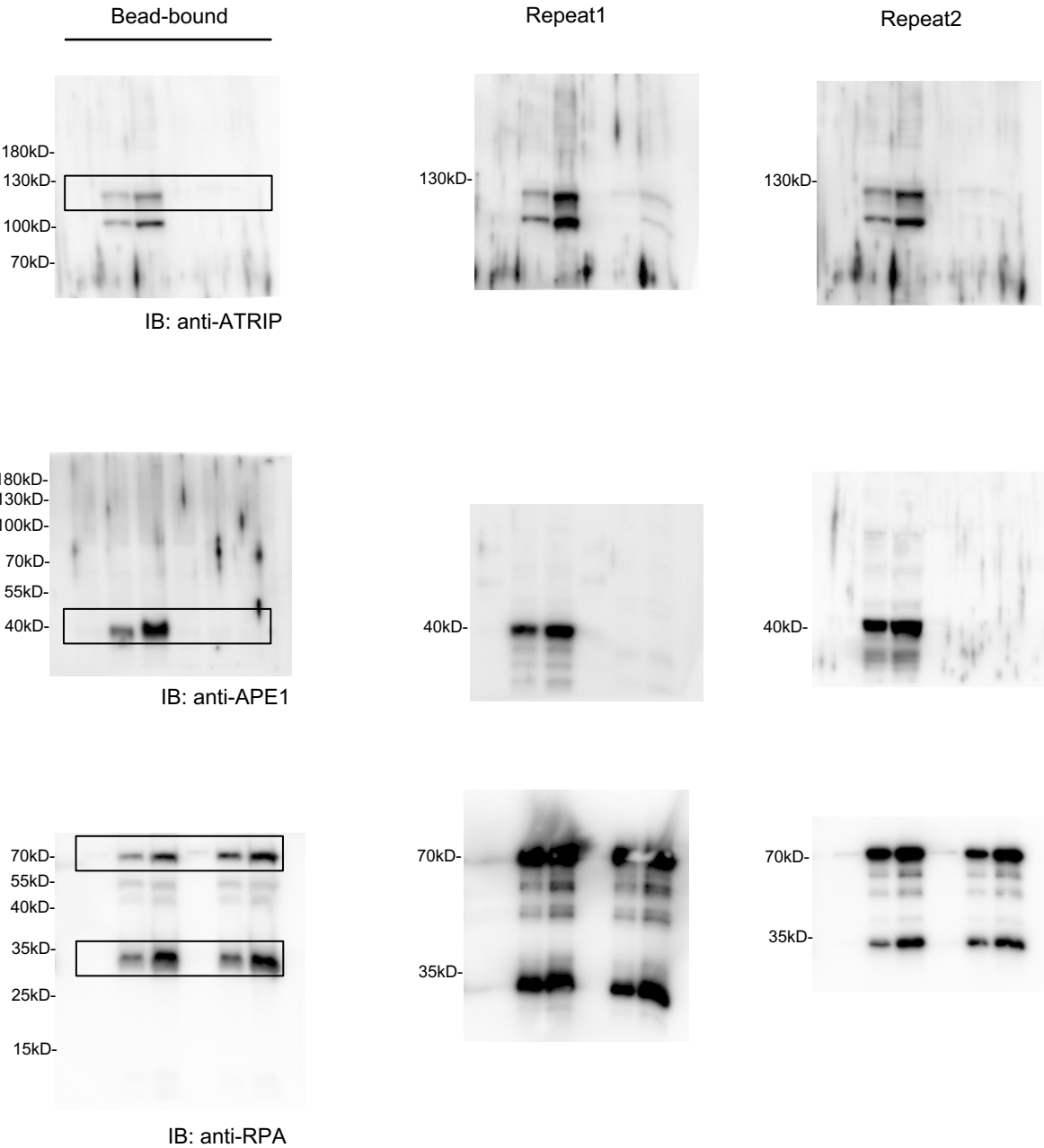

Figure 1B

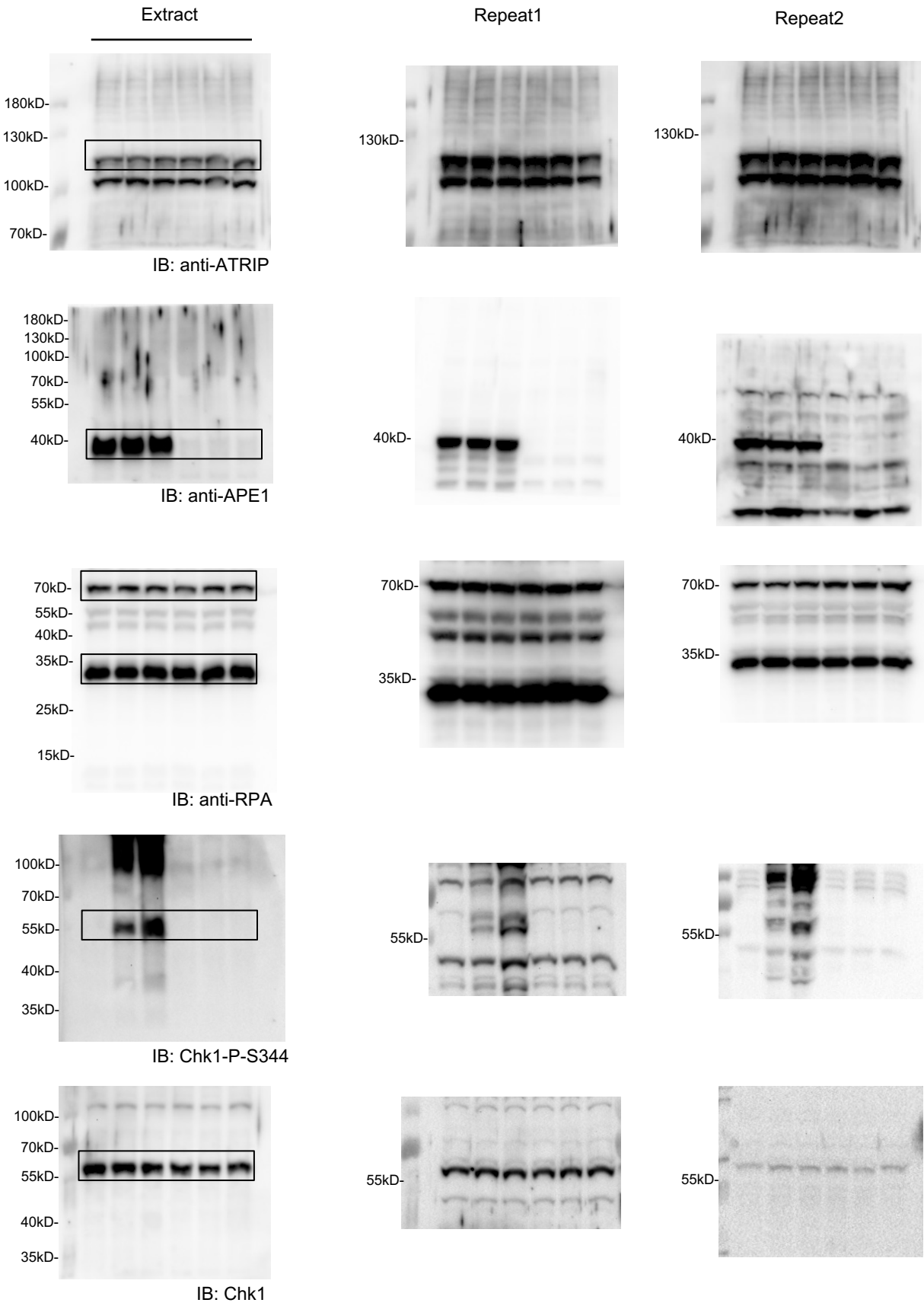

Supplement: Figure 1—source data 2. [file elife-82324-fig1-data2.zip › Figure 1-source data 2/IB-data-Figure 1B.pdf]

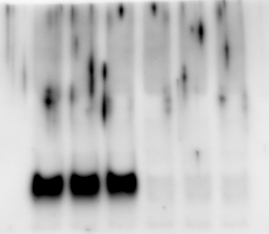

Supplement: Figure 1—source data 2. [file elife-82324-fig1-data2.zip › Figure 1-source data 2/Figure 1B initial trial/Extract-APE1.tif]

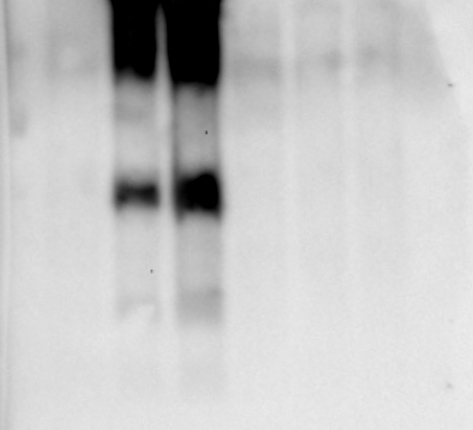

Supplement: Figure 1—source data 2. [file elife-82324-fig1-data2.zip › Figure 1-source data 2/Figure 1B initial trial/Extract-Chk1-P-S344.tif]

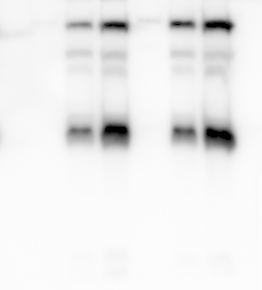

Supplement: Figure 1—source data 2. [file elife-82324-fig1-data2.zip › Figure 1-source data 2/Figure 1B initial trial/Bead-bound-RPA.tif]

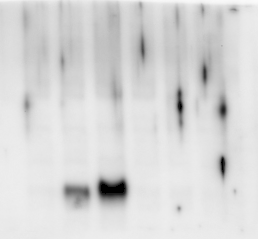

Supplement: Figure 1—source data 2. [file elife-82324-fig1-data2.zip › Figure 1-source data 2/Figure 1B initial trial/Bead-bound-APE1.tif]

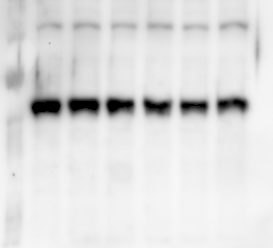

Supplement: Figure 1—source data 2. [file elife-82324-fig1-data2.zip › Figure 1-source data 2/Figure 1B initial trial/Extract-Chk1.tif]

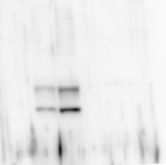

Supplement: Figure 1—source data 2. [file elife-82324-fig1-data2.zip › Figure 1-source data 2/Figure 1B initial trial/Bead-bound-ATRIP.tif]

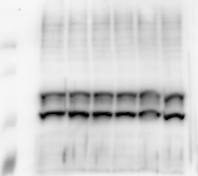

Supplement: Figure 1—source data 2. [file elife-82324-fig1-data2.zip › Figure 1-source data 2/Figure 1B initial trial/Extract-ATRIP.tif]

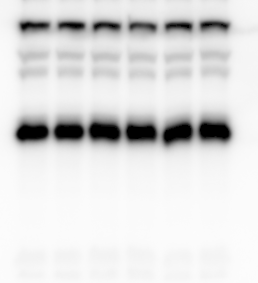

Supplement: Figure 1—source data 2. [file elife-82324-fig1-data2.zip › Figure 1-source data 2/Figure 1B initial trial/Extract-RPA.tif]

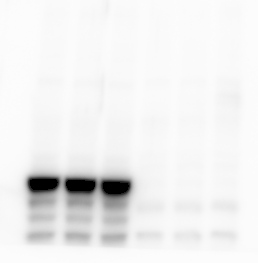

Supplement: Figure 1—source data 2. [file elife-82324-fig1-data2.zip › Figure 1-source data 2/Figure 1B Repeat1/Extract-APE1.tif]

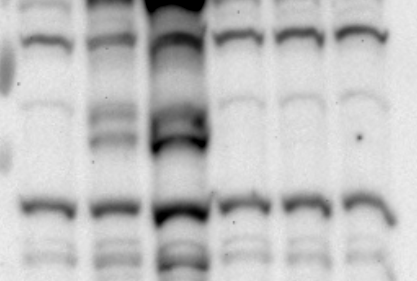

Supplement: Figure 1—source data 2. [file elife-82324-fig1-data2.zip › Figure 1-source data 2/Figure 1B Repeat1/Extract-Chk1-P-S344.tif]

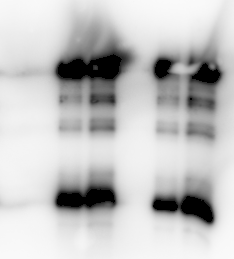

Supplement: Figure 1—source data 2. [file elife-82324-fig1-data2.zip › Figure 1-source data 2/Figure 1B Repeat1/Bead-bound-RPA.tif]

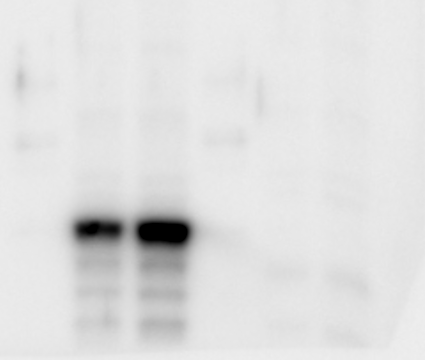

Supplement: Figure 1—source data 2. [file elife-82324-fig1-data2.zip › Figure 1-source data 2/Figure 1B Repeat1/Bead-bound-APE1.tif]

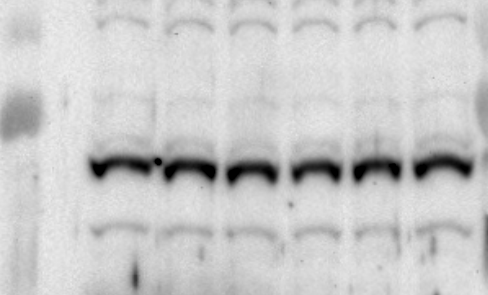

Supplement: Figure 1—source data 2. [file elife-82324-fig1-data2.zip › Figure 1-source data 2/Figure 1B Repeat1/Extract-Chk1.tif]

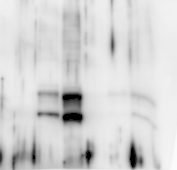

Supplement: Figure 1—source data 2. [file elife-82324-fig1-data2.zip › Figure 1-source data 2/Figure 1B Repeat1/Bead-bound-ATRIP.tif]

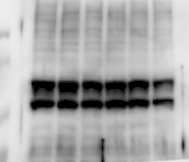

Supplement: Figure 1—source data 2. [file elife-82324-fig1-data2.zip › Figure 1-source data 2/Figure 1B Repeat1/Extract-ATRIP.tif]

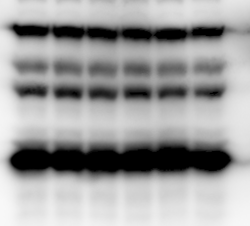

Supplement: Figure 1—source data 2. [file elife-82324-fig1-data2.zip › Figure 1-source data 2/Figure 1B Repeat1/Extract-RPA.tif]

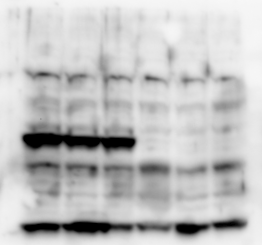

Supplement: Figure 1—source data 2. [file elife-82324-fig1-data2.zip › Figure 1-source data 2/Figure 1B Repeat2/Extract-APE1.tif]

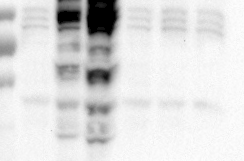

Supplement: Figure 1—source data 2. [file elife-82324-fig1-data2.zip › Figure 1-source data 2/Figure 1B Repeat2/Extract-Chk1-P-S344.tif]

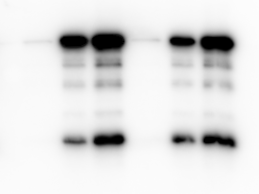

Supplement: Figure 1—source data 2. [file elife-82324-fig1-data2.zip › Figure 1-source data 2/Figure 1B Repeat2/Bead-bound-RPA.tif]

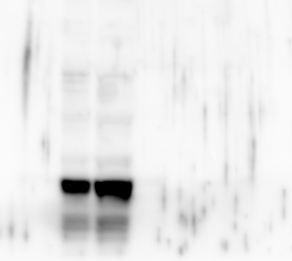

Supplement: Figure 1—source data 2. [file elife-82324-fig1-data2.zip › Figure 1-source data 2/Figure 1B Repeat2/Bead-bound-APE1.tif]

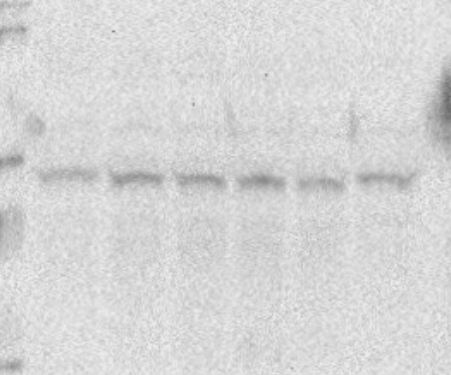

Supplement: Figure 1—source data 2. [file elife-82324-fig1-data2.zip › Figure 1-source data 2/Figure 1B Repeat2/Extract-Chk1.tif]

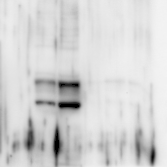

Supplement: Figure 1—source data 2. [file elife-82324-fig1-data2.zip › Figure 1-source data 2/Figure 1B Repeat2/Bead-bound-ATRIP.tif]

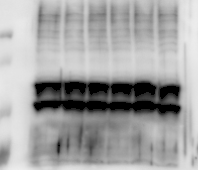

Supplement: Figure 1—source data 2. [file elife-82324-fig1-data2.zip › Figure 1-source data 2/Figure 1B Repeat2/Extract-ATRIP.tif]

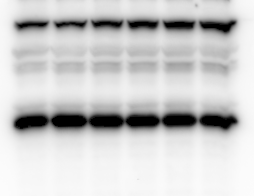

Supplement: Figure 1—source data 2. [file elife-82324-fig1-data2.zip › Figure 1-source data 2/Figure 1B Repeat2/Extract-RPA.tif]

Figure 1C

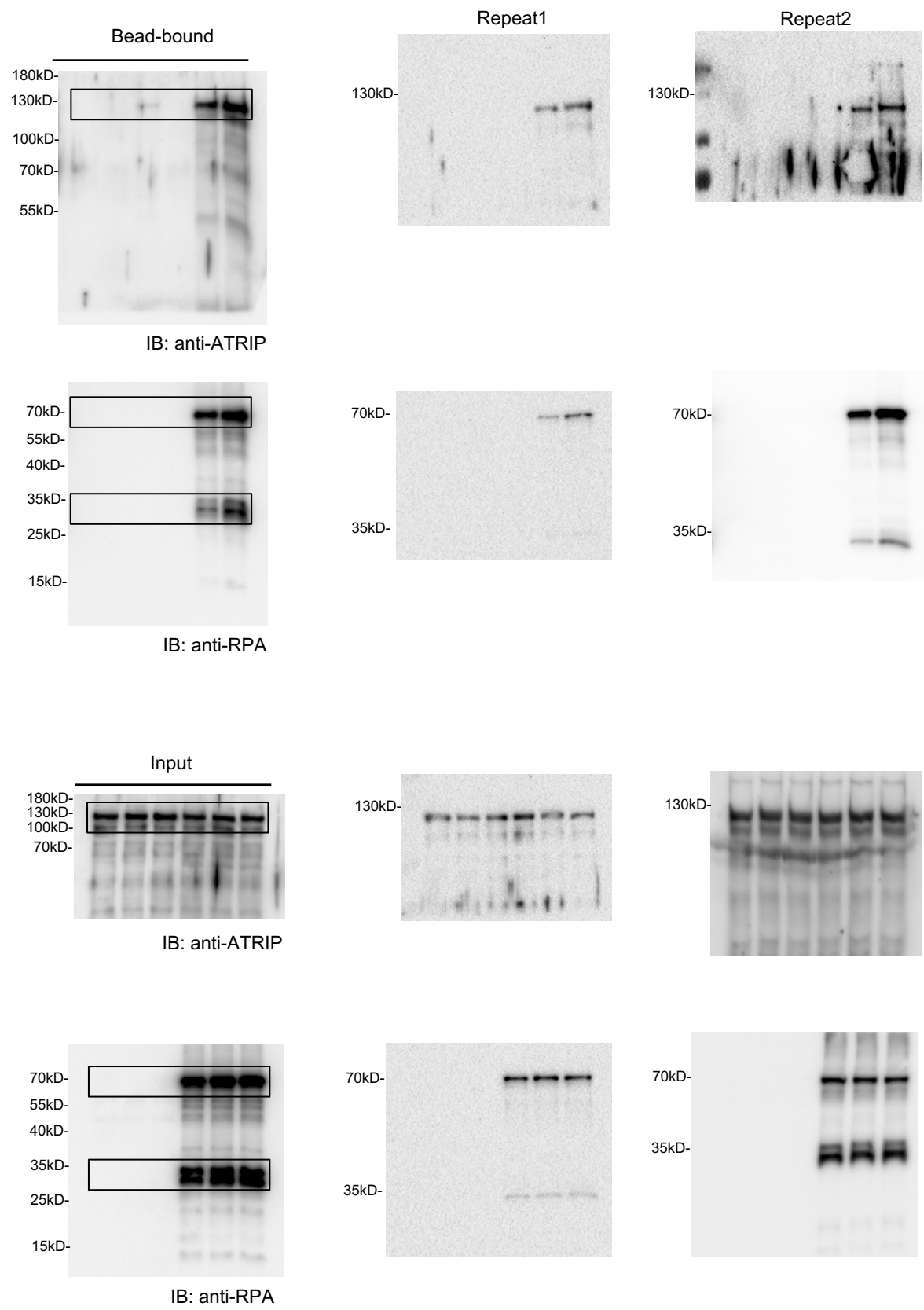

Supplement: Figure 1—source data 3. [file elife-82324-fig1-data3.zip › Figure 1-source data 3/IB-data-Figure 1C.pdf]

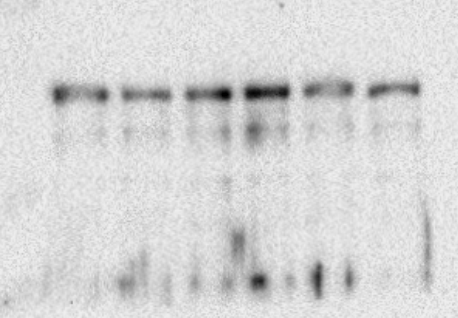

Supplement: Figure 1—source data 3. [file elife-82324-fig1-data3.zip › Figure 1-source data 3/Figure 1C Repeat1/Input-ATRIP.tif]

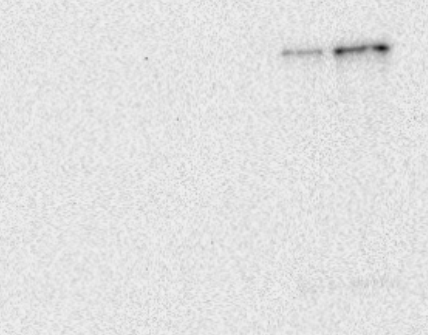

Supplement: Figure 1—source data 3. [file elife-82324-fig1-data3.zip › Figure 1-source data 3/Figure 1C Repeat1/Bead-bound-RPA.tif]

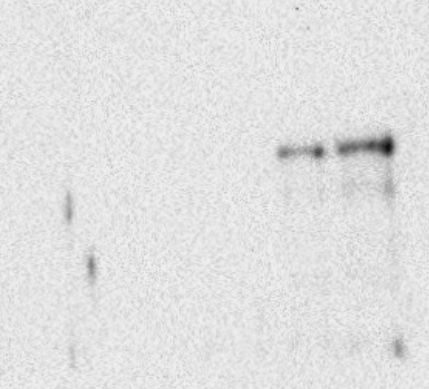

Supplement: Figure 1—source data 3. [file elife-82324-fig1-data3.zip › Figure 1-source data 3/Figure 1C Repeat1/Bead-bound-ATRIP.tif]

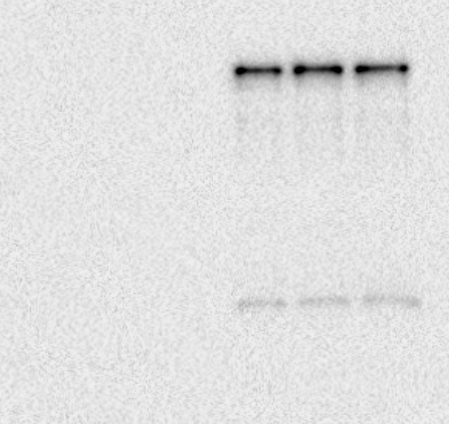

Supplement: Figure 1—source data 3. [file elife-82324-fig1-data3.zip › Figure 1-source data 3/Figure 1C Repeat1/Input-RPA.tif]

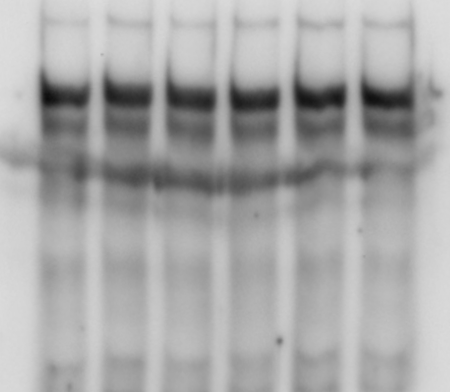

Supplement: Figure 1—source data 3. [file elife-82324-fig1-data3.zip › Figure 1-source data 3/Figure 1C Repeat2/Input-ATRIP.tif]

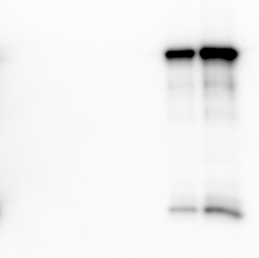

Supplement: Figure 1—source data 3. [file elife-82324-fig1-data3.zip › Figure 1-source data 3/Figure 1C Repeat2/Bead-bound-RPA.tif]

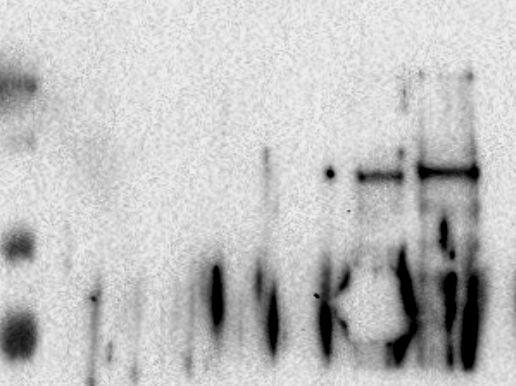

Supplement: Figure 1—source data 3. [file elife-82324-fig1-data3.zip › Figure 1-source data 3/Figure 1C Repeat2/Bead-bound-ATRIP.tif]

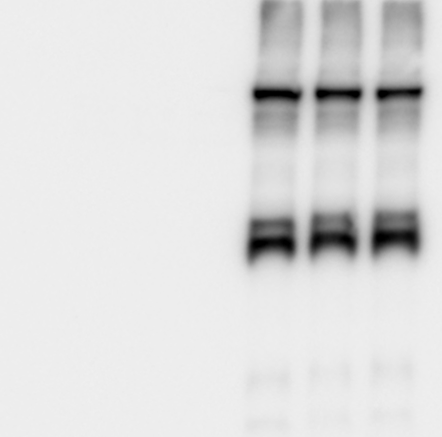

Supplement: Figure 1—source data 3. [file elife-82324-fig1-data3.zip › Figure 1-source data 3/Figure 1C Repeat2/Input-RPA.tif]

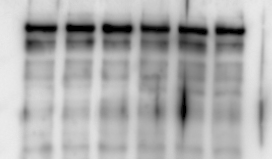

Supplement: Figure 1—source data 3. [file elife-82324-fig1-data3.zip › Figure 1-source data 3/Figure 1C intial trial/Input-ATRIP.tif]

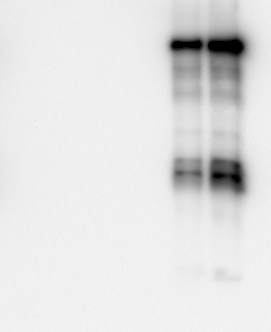

Supplement: Figure 1—source data 3. [file elife-82324-fig1-data3.zip › Figure 1-source data 3/Figure 1C intial trial/Bead-bound-RPA.tif]

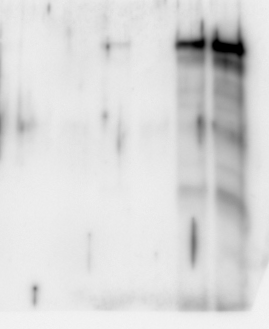

Supplement: Figure 1—source data 3. [file elife-82324-fig1-data3.zip › Figure 1-source data 3/Figure 1C intial trial/Bead-bound-ATRIP.tif]

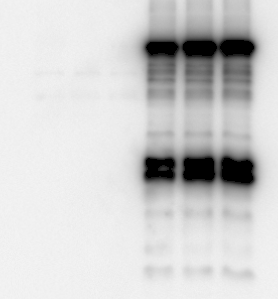

Supplement: Figure 1—source data 3. [file elife-82324-fig1-data3.zip › Figure 1-source data 3/Figure 1C intial trial/Input-RPA.tif]

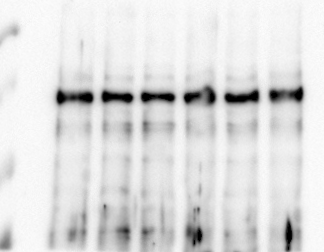

Supplement: Figure 1—source data 4. [file elife-82324-fig1-data4.zip › Figure 1-source data 4/Figure 1D Repeat2/Input-ATRIP.tif]

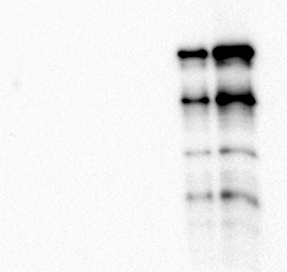

Supplement: Figure 1—source data 4. [file elife-82324-fig1-data4.zip › Figure 1-source data 4/Figure 1D Repeat2/Bead-bound-GST.tif]

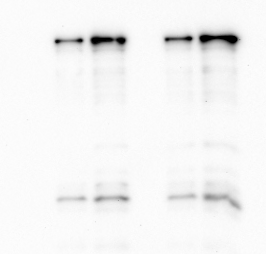

Supplement: Figure 1—source data 4. [file elife-82324-fig1-data4.zip › Figure 1-source data 4/Figure 1D Repeat2/Bead-bound-RPA.tif]

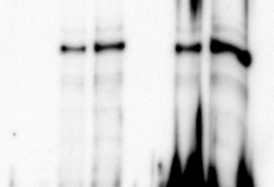

Supplement: Figure 1—source data 4. [file elife-82324-fig1-data4.zip › Figure 1-source data 4/Figure 1D Repeat2/Bead-bound-ATRIP.tif]

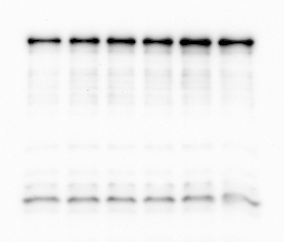

Supplement: Figure 1—source data 4. [file elife-82324-fig1-data4.zip › Figure 1-source data 4/Figure 1D Repeat2/Input-RPA.tif]

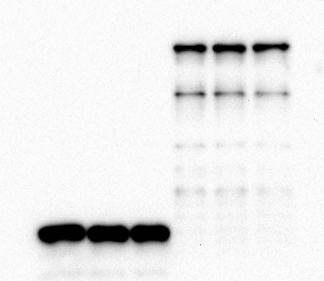

Supplement: Figure 1—source data 4. [file elife-82324-fig1-data4.zip › Figure 1-source data 4/Figure 1D Repeat2/Input-GST.tif]

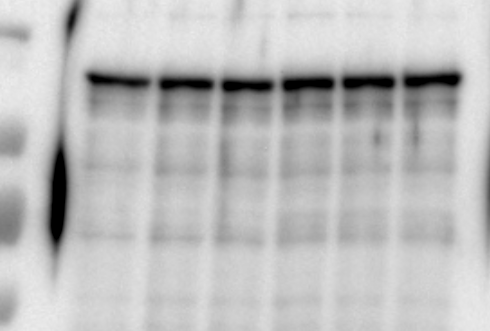

Supplement: Figure 1—source data 4. [file elife-82324-fig1-data4.zip › Figure 1-source data 4/Figure 1D Repeat1/Input-ATRIP.tif]

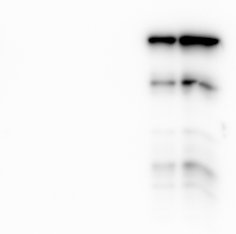

Supplement: Figure 1—source data 4. [file elife-82324-fig1-data4.zip › Figure 1-source data 4/Figure 1D Repeat1/Bead-bound-GST.tif]

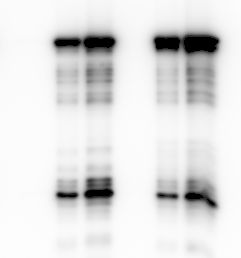

Supplement: Figure 1—source data 4. [file elife-82324-fig1-data4.zip › Figure 1-source data 4/Figure 1D Repeat1/Bead-bound-RPA.tif]

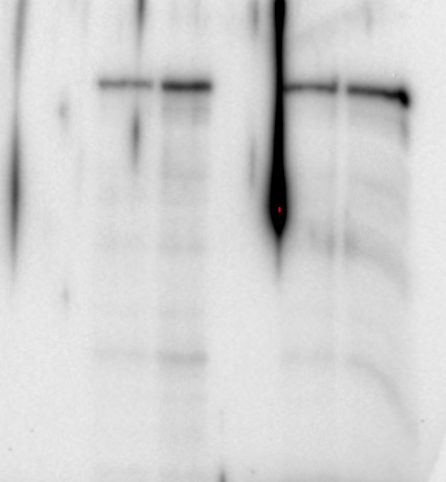

Supplement: Figure 1—source data 4. [file elife-82324-fig1-data4.zip › Figure 1-source data 4/Figure 1D Repeat1/Bead-bound-ATRIP.tif]

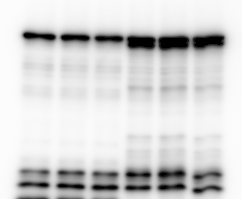

Supplement: Figure 1—source data 4. [file elife-82324-fig1-data4.zip › Figure 1-source data 4/Figure 1D Repeat1/Input-RPA.tif]

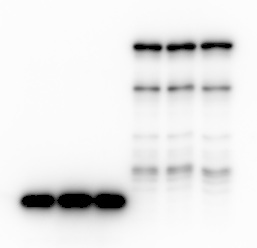

Supplement: Figure 1—source data 4. [file elife-82324-fig1-data4.zip › Figure 1-source data 4/Figure 1D Repeat1/Input-GST.tif]

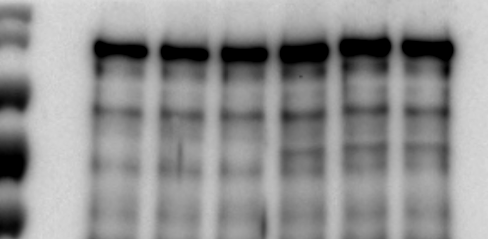

Supplement: Figure 1—source data 4. [file elife-82324-fig1-data4.zip › Figure 1-source data 4/Figure 1D intial trial/Input-ATRIP.tif]

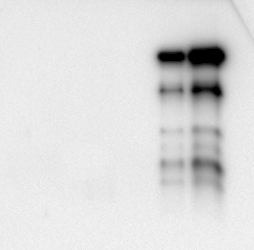

Supplement: Figure 1—source data 4. [file elife-82324-fig1-data4.zip › Figure 1-source data 4/Figure 1D intial trial/Bead-bound-GST.tif]

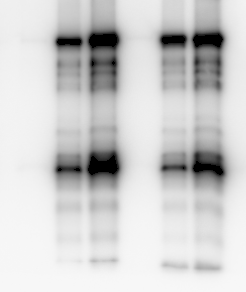

Supplement: Figure 1—source data 4. [file elife-82324-fig1-data4.zip › Figure 1-source data 4/Figure 1D intial trial/Bead-bound-RPA.tif]

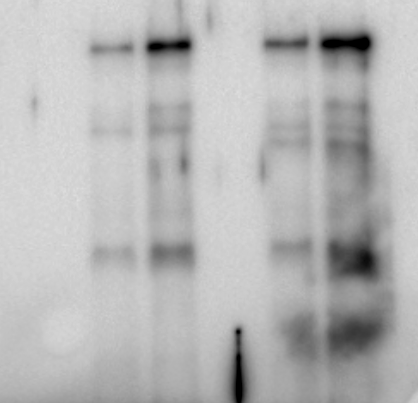

Supplement: Figure 1—source data 4. [file elife-82324-fig1-data4.zip › Figure 1-source data 4/Figure 1D intial trial/Bead-bound-ATRIP.tif]

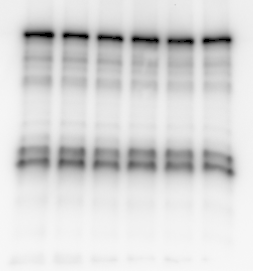

Supplement: Figure 1—source data 4. [file elife-82324-fig1-data4.zip › Figure 1-source data 4/Figure 1D intial trial/Input-RPA.tif]

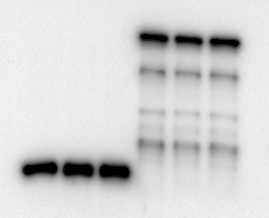

Supplement: Figure 1—source data 4. [file elife-82324-fig1-data4.zip › Figure 1-source data 4/Figure 1D intial trial/Input-GST.tif]

Figure 1-figure supplement 1A

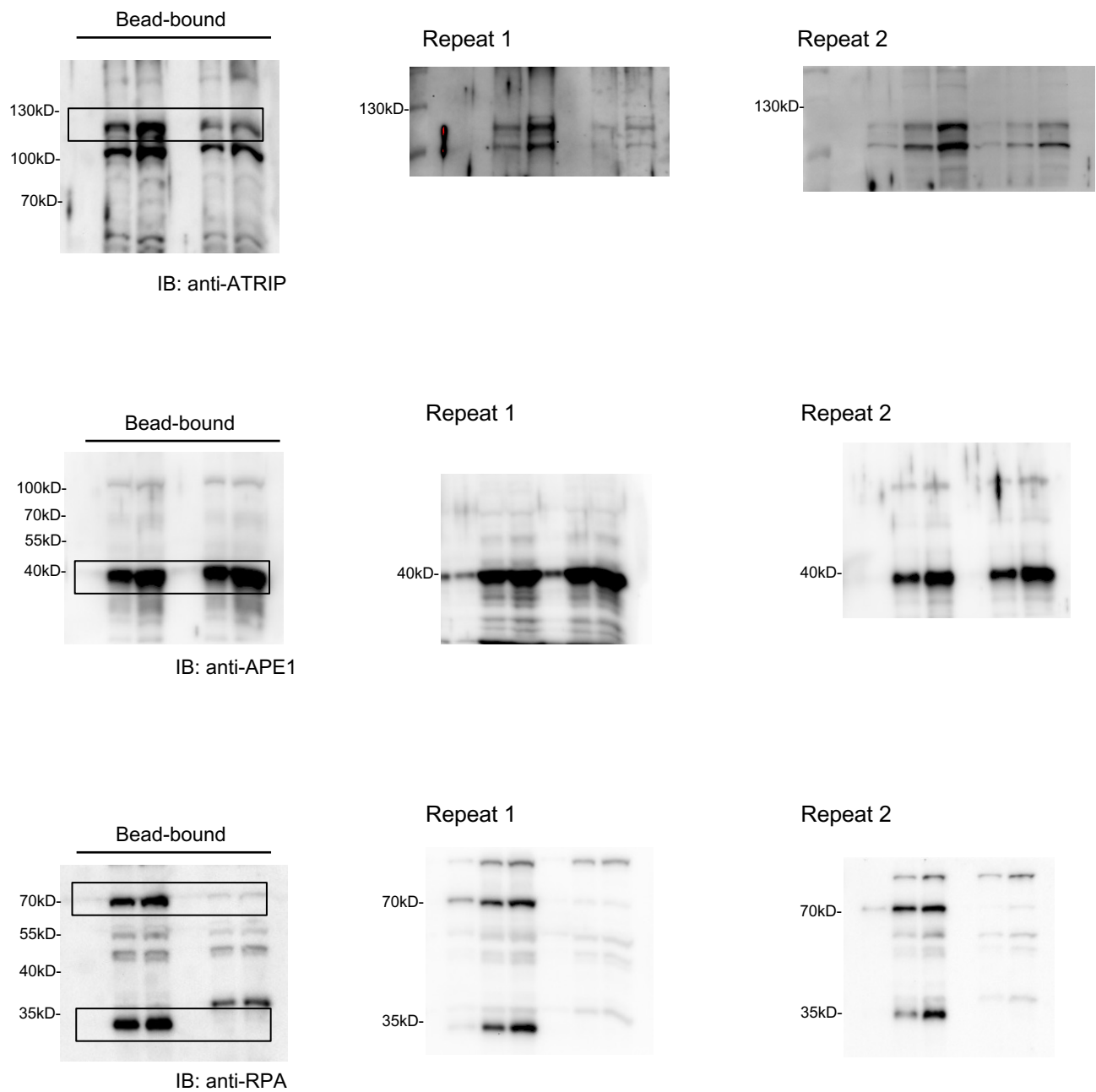

Figure 1-figure supplement 1A

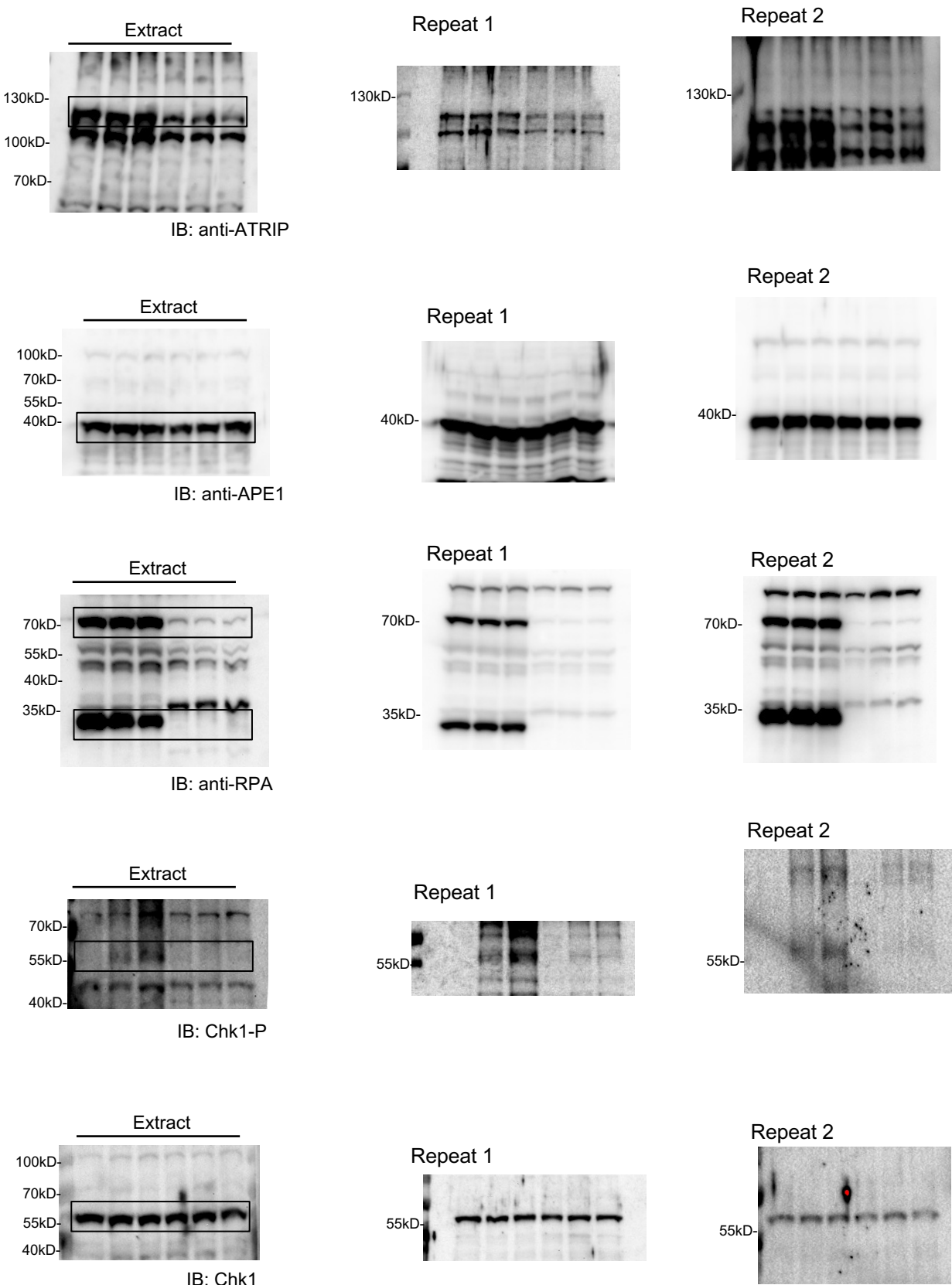

Supplement: Figure 1—figure supplement 1—source data 1. [file elife-82324-fig1-figsupp1-data1.zip › Figure 1-figure supplement 1-souce data 1/IB-data-Figure 1S1A.pdf]

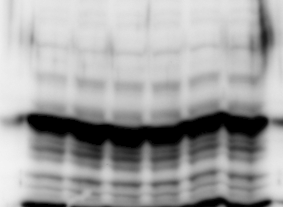

Supplement: Figure 1—figure supplement 1—source data 1. [file elife-82324-fig1-figsupp1-data1.zip › Figure 1-figure supplement 1-souce data 1/Figure 1S1A Repeat1/Extract-APE1.tif]

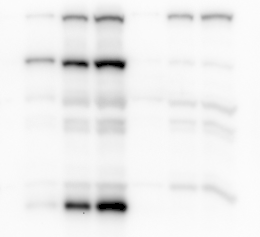

Supplement: Figure 1—figure supplement 1—source data 1. [file elife-82324-fig1-figsupp1-data1.zip › Figure 1-figure supplement 1-souce data 1/Figure 1S1A Repeat1/Bead-RPA.tif]

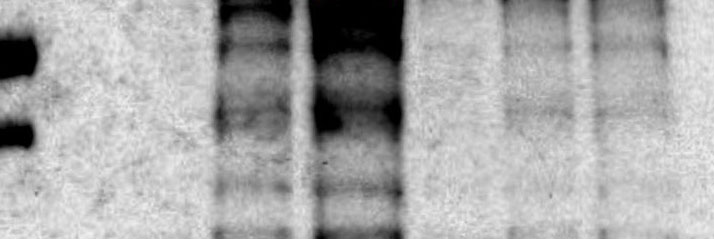

Supplement: Figure 1—figure supplement 1—source data 1. [file elife-82324-fig1-figsupp1-data1.zip › Figure 1-figure supplement 1-souce data 1/Figure 1S1A Repeat1/Extract-Chk1-P.jpg]

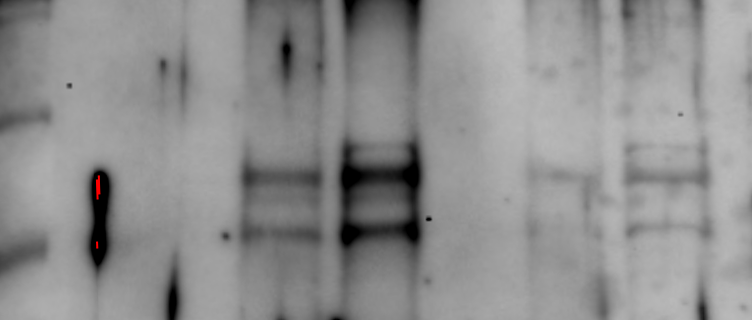

Supplement: Figure 1—figure supplement 1—source data 1. [file elife-82324-fig1-figsupp1-data1.zip › Figure 1-figure supplement 1-souce data 1/Figure 1S1A Repeat1/Bead-ATRIP.tif]

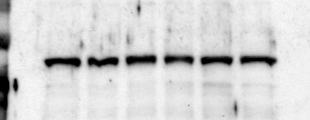

Supplement: Figure 1—figure supplement 1—source data 1. [file elife-82324-fig1-figsupp1-data1.zip › Figure 1-figure supplement 1-souce data 1/Figure 1S1A Repeat1/Extract-Chk1.tif]

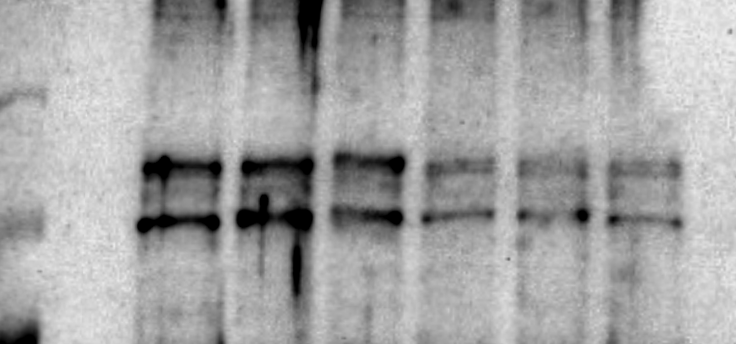

Supplement: Figure 1—figure supplement 1—source data 1. [file elife-82324-fig1-figsupp1-data1.zip › Figure 1-figure supplement 1-souce data 1/Figure 1S1A Repeat1/Extract-ATRIP.tif]

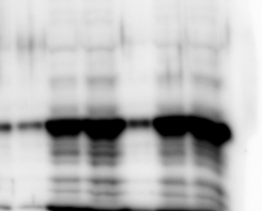

Supplement: Figure 1—figure supplement 1—source data 1. [file elife-82324-fig1-figsupp1-data1.zip › Figure 1-figure supplement 1-souce data 1/Figure 1S1A Repeat1/Bead-APE1.tif]

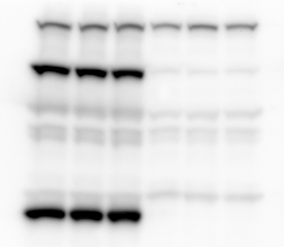

Supplement: Figure 1—figure supplement 1—source data 1. [file elife-82324-fig1-figsupp1-data1.zip › Figure 1-figure supplement 1-souce data 1/Figure 1S1A Repeat1/Extract-RPA.tif]

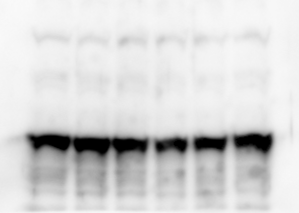

Supplement: Figure 1—figure supplement 1—source data 1. [file elife-82324-fig1-figsupp1-data1.zip › Figure 1-figure supplement 1-souce data 1/Figure 1S1A intial trial/Extract-APE1.tif]

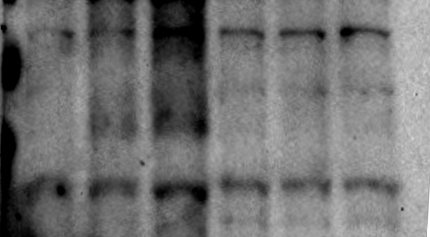

Supplement: Figure 1—figure supplement 1—source data 1. [file elife-82324-fig1-figsupp1-data1.zip › Figure 1-figure supplement 1-souce data 1/Figure 1S1A intial trial/Extract-Chk1-P.tif]

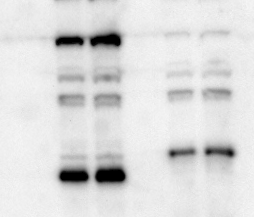

Supplement: Figure 1—figure supplement 1—source data 1. [file elife-82324-fig1-figsupp1-data1.zip › Figure 1-figure supplement 1-souce data 1/Figure 1S1A intial trial/Bead-RPA.tif]

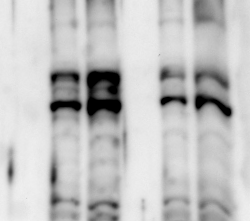

Supplement: Figure 1—figure supplement 1—source data 1. [file elife-82324-fig1-figsupp1-data1.zip › Figure 1-figure supplement 1-souce data 1/Figure 1S1A intial trial/Bead-ATRIP.tif]

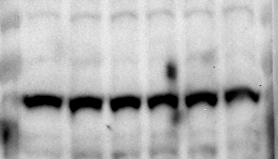

Supplement: Figure 1—figure supplement 1—source data 1. [file elife-82324-fig1-figsupp1-data1.zip › Figure 1-figure supplement 1-souce data 1/Figure 1S1A intial trial/Extract-Chk1.tif]

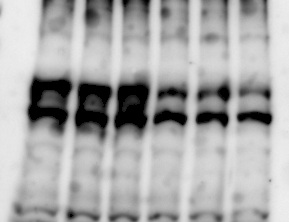

Supplement: Figure 1—figure supplement 1—source data 1. [file elife-82324-fig1-figsupp1-data1.zip › Figure 1-figure supplement 1-souce data 1/Figure 1S1A intial trial/Extract-ATRIP.tif]

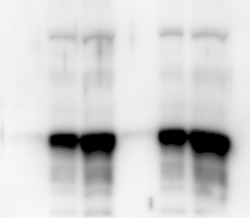

Supplement: Figure 1—figure supplement 1—source data 1. [file elife-82324-fig1-figsupp1-data1.zip › Figure 1-figure supplement 1-souce data 1/Figure 1S1A intial trial/Bead-APE1.tif]
