# Supplementary material for: APE1 recruits ATRIP to ssDNA in an RPA-dependent and -independent manner to promote the ATR DNA damage response
Source: eLife. 2023 May 22;12:e82324. doi: 10.7554/eLife.82324 (PMC10202453; doi:10.7554/eLife.82324)
Supplement: Figure 1—source data 4. [file elife-82324-fig1-data4.zip › Figure 1-source data 4/IB-data-Figure 1D.pdf]

Figure 1D

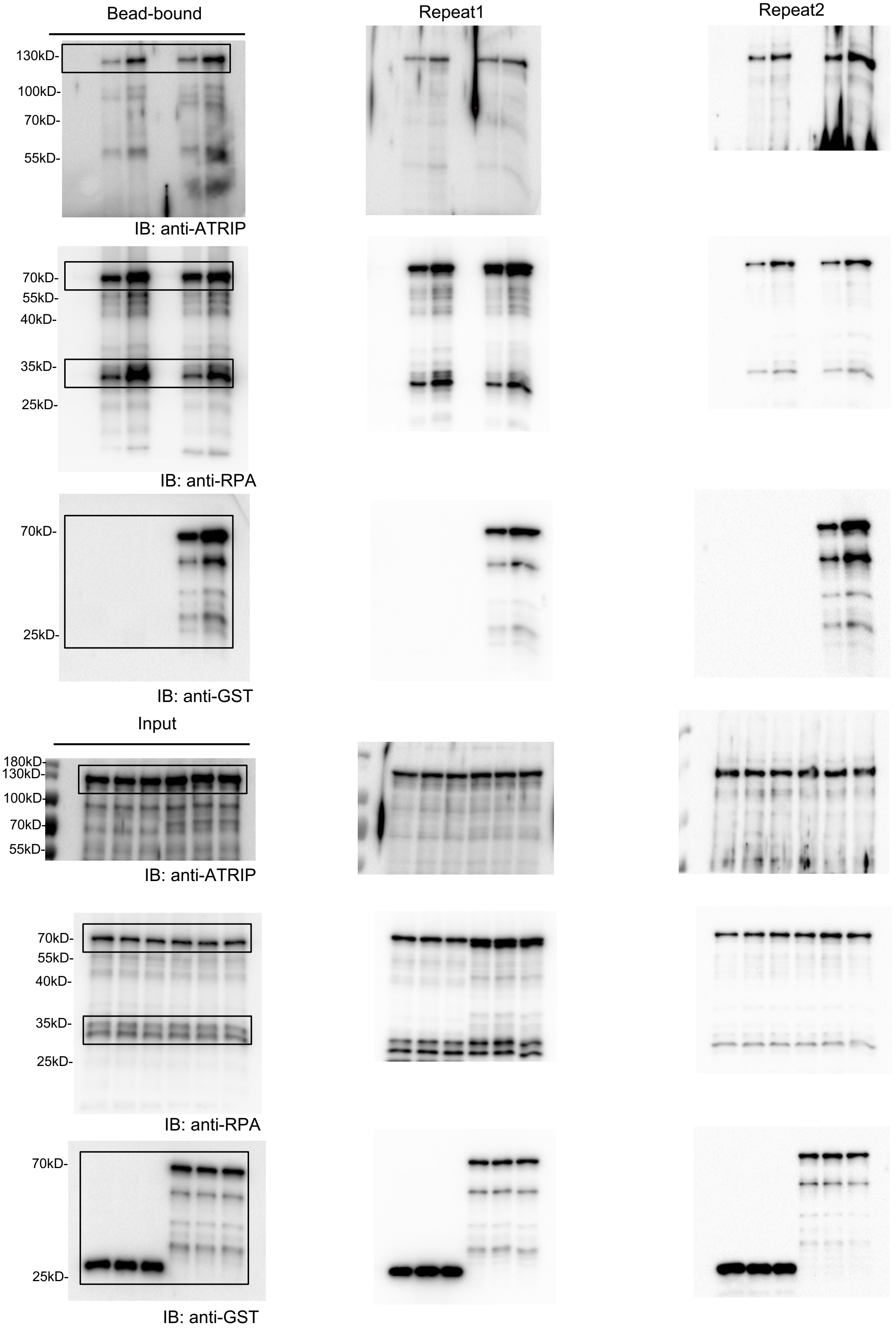
Bead-bound
Repeat1
Repeat2
130kD-
100kD-
70kD-
55kD-
IB: anti-ATRIP
70kD-
55kD-
40kD-
35kD-
25kD-
IB: anti-RPA
70kD-
25kD-
IB: anti-GST
Input
180kD-
130kD-
100kD-
70kD-
55kD-
IB: anti-ATRIP
70kD-
55kD-
40kD-
35kD-
25kD-
IB: anti-RPA
70kD-
25kD-
IB: anti-GST
