# Supplementary figures and images for: APE1 recruits ATRIP to ssDNA in an RPA-dependent and -independent manner to promote the ATR DNA damage response (part 2 of 4)
Source: eLife. 2023 May 22;12:e82324. doi: 10.7554/eLife.82324 (PMC10202453; doi:10.7554/eLife.82324)

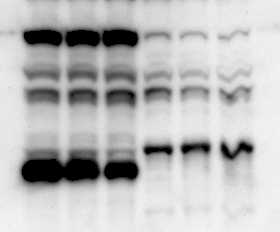

Supplement: Figure 1—figure supplement 1—source data 1. [file elife-82324-fig1-figsupp1-data1.zip › Figure 1-figure supplement 1-souce data 1/Figure 1S1A intial trial/Extract-RPA.tif]

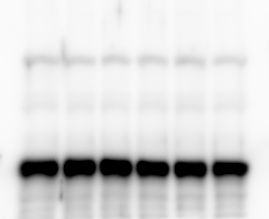

Supplement: Figure 1—figure supplement 1—source data 1. [file elife-82324-fig1-figsupp1-data1.zip › Figure 1-figure supplement 1-souce data 1/Figure 1S1A Repeat2/Extract-APE1.tif]

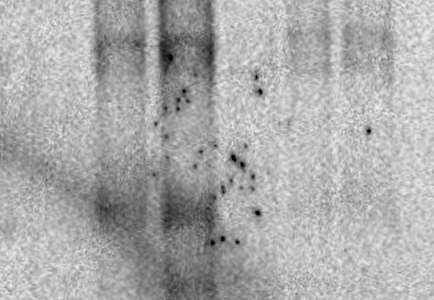

Supplement: Figure 1—figure supplement 1—source data 1. [file elife-82324-fig1-figsupp1-data1.zip › Figure 1-figure supplement 1-souce data 1/Figure 1S1A Repeat2/Extract-Chk1-P.tif]

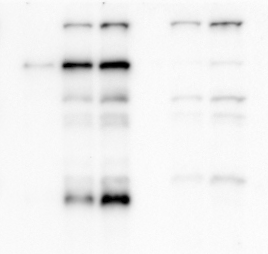

Supplement: Figure 1—figure supplement 1—source data 1. [file elife-82324-fig1-figsupp1-data1.zip › Figure 1-figure supplement 1-souce data 1/Figure 1S1A Repeat2/Bead-RPA.tif]

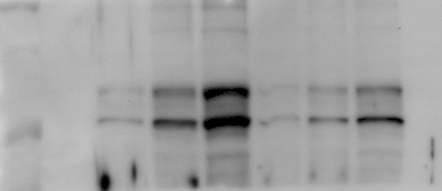

Supplement: Figure 1—figure supplement 1—source data 1. [file elife-82324-fig1-figsupp1-data1.zip › Figure 1-figure supplement 1-souce data 1/Figure 1S1A Repeat2/Bead-ATRIP.tif]

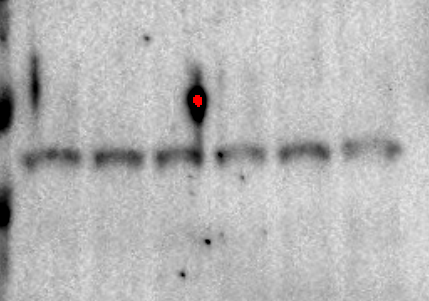

Supplement: Figure 1—figure supplement 1—source data 1. [file elife-82324-fig1-figsupp1-data1.zip › Figure 1-figure supplement 1-souce data 1/Figure 1S1A Repeat2/Extract-Chk1.tif]

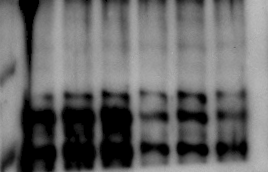

Supplement: Figure 1—figure supplement 1—source data 1. [file elife-82324-fig1-figsupp1-data1.zip › Figure 1-figure supplement 1-souce data 1/Figure 1S1A Repeat2/Extract-ATRIP.tif]

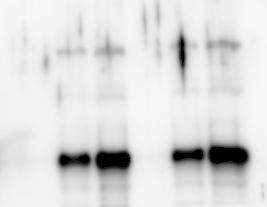

Supplement: Figure 1—figure supplement 1—source data 1. [file elife-82324-fig1-figsupp1-data1.zip › Figure 1-figure supplement 1-souce data 1/Figure 1S1A Repeat2/Bead-APE1.tif]

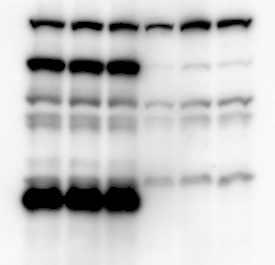

Supplement: Figure 1—figure supplement 1—source data 1. [file elife-82324-fig1-figsupp1-data1.zip › Figure 1-figure supplement 1-souce data 1/Figure 1S1A Repeat2/Extract-RPA.tif]

Figure 1-figure supplement 1B

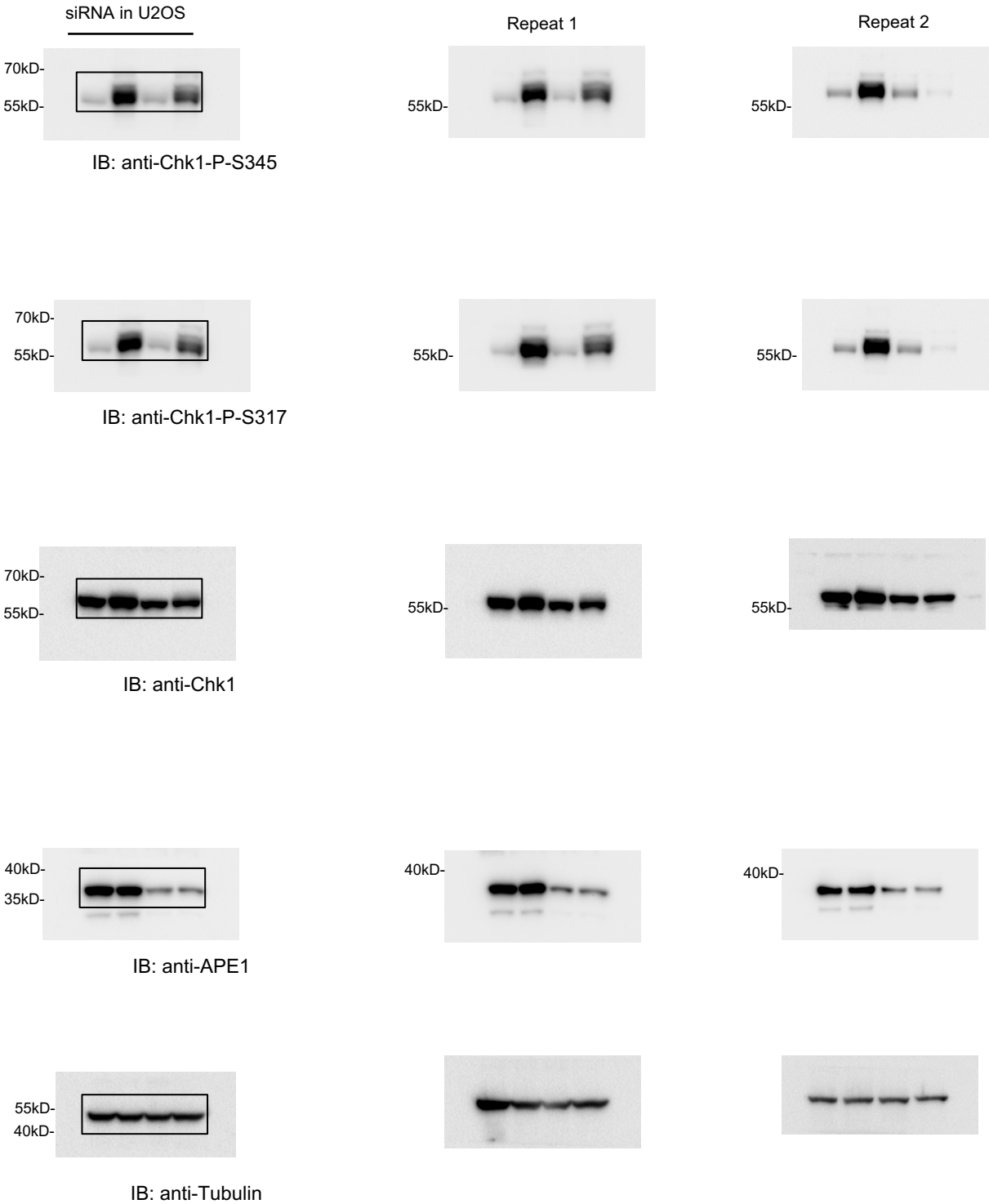

Supplement: Figure 1—figure supplement 1—source data 2. [file elife-82324-fig1-figsupp1-data2.zip › Figure 1-figure supplement 1-souce data 2/IB-data-Figure 1S1B.pdf]

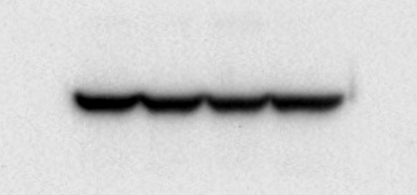

Supplement: Figure 1—figure supplement 1—source data 2. [file elife-82324-fig1-figsupp1-data2.zip › Figure 1-figure supplement 1-souce data 2/Figure 1S1B initial trial/Tubulin.tif]

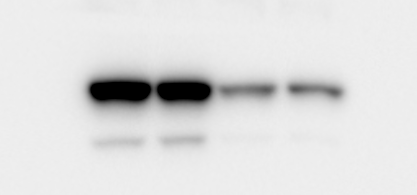

Supplement: Figure 1—figure supplement 1—source data 2. [file elife-82324-fig1-figsupp1-data2.zip › Figure 1-figure supplement 1-souce data 2/Figure 1S1B initial trial/APE1.tif]

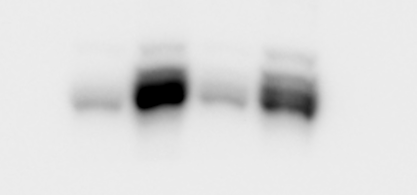

Supplement: Figure 1—figure supplement 1—source data 2. [file elife-82324-fig1-figsupp1-data2.zip › Figure 1-figure supplement 1-souce data 2/Figure 1S1B initial trial/Chk1-P-S317.tif]

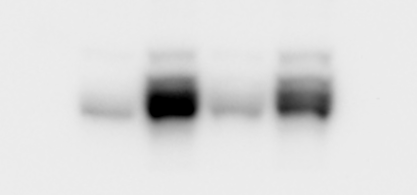

Supplement: Figure 1—figure supplement 1—source data 2. [file elife-82324-fig1-figsupp1-data2.zip › Figure 1-figure supplement 1-souce data 2/Figure 1S1B initial trial/Chk1-P-S345.tif]

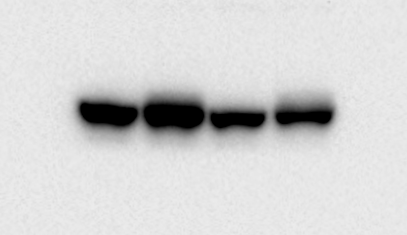

Supplement: Figure 1—figure supplement 1—source data 2. [file elife-82324-fig1-figsupp1-data2.zip › Figure 1-figure supplement 1-souce data 2/Figure 1S1B initial trial/Chk1.tif]

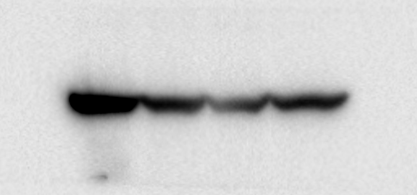

Supplement: Figure 1—figure supplement 1—source data 2. [file elife-82324-fig1-figsupp1-data2.zip › Figure 1-figure supplement 1-souce data 2/Figure 1S1B Repeat1/Tubulin.tif]

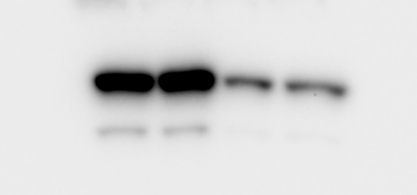

Supplement: Figure 1—figure supplement 1—source data 2. [file elife-82324-fig1-figsupp1-data2.zip › Figure 1-figure supplement 1-souce data 2/Figure 1S1B Repeat1/APE1.tif]

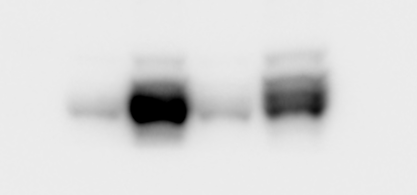

Supplement: Figure 1—figure supplement 1—source data 2. [file elife-82324-fig1-figsupp1-data2.zip › Figure 1-figure supplement 1-souce data 2/Figure 1S1B Repeat1/Chk1-P-S317.tif]

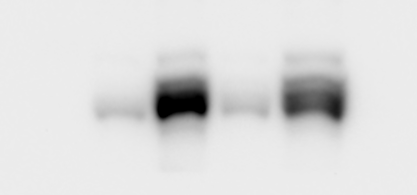

Supplement: Figure 1—figure supplement 1—source data 2. [file elife-82324-fig1-figsupp1-data2.zip › Figure 1-figure supplement 1-souce data 2/Figure 1S1B Repeat1/Chk1-P-S345.tif]

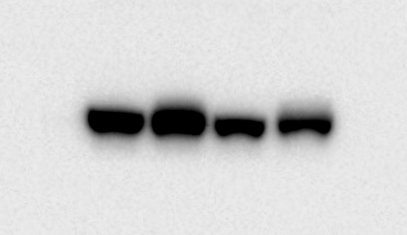

Supplement: Figure 1—figure supplement 1—source data 2. [file elife-82324-fig1-figsupp1-data2.zip › Figure 1-figure supplement 1-souce data 2/Figure 1S1B Repeat1/Chk1.tif]

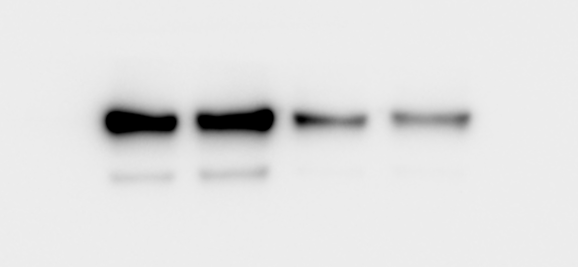

Supplement: Figure 1—figure supplement 1—source data 2. [file elife-82324-fig1-figsupp1-data2.zip › Figure 1-figure supplement 1-souce data 2/Figure 1S1B Repeat2/APE1.tif]

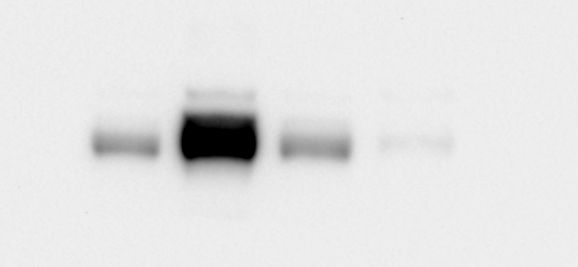

Supplement: Figure 1—figure supplement 1—source data 2. [file elife-82324-fig1-figsupp1-data2.zip › Figure 1-figure supplement 1-souce data 2/Figure 1S1B Repeat2/Chk1-P-S317.tif]

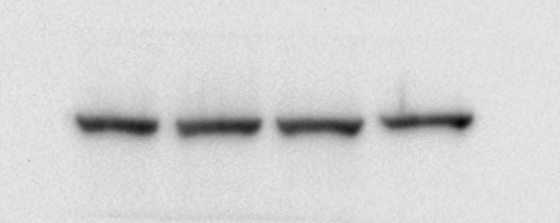

Supplement: Figure 1—figure supplement 1—source data 2. [file elife-82324-fig1-figsupp1-data2.zip › Figure 1-figure supplement 1-souce data 2/Figure 1S1B Repeat2/Tubulin-3st.tif]

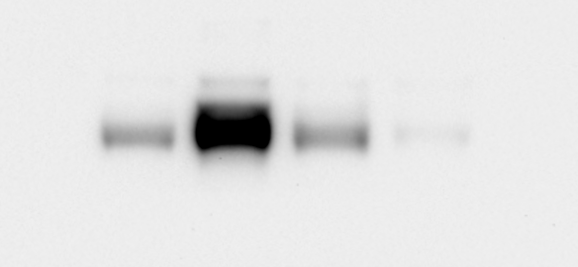

Supplement: Figure 1—figure supplement 1—source data 2. [file elife-82324-fig1-figsupp1-data2.zip › Figure 1-figure supplement 1-souce data 2/Figure 1S1B Repeat2/Chk1-P-S345.tif]

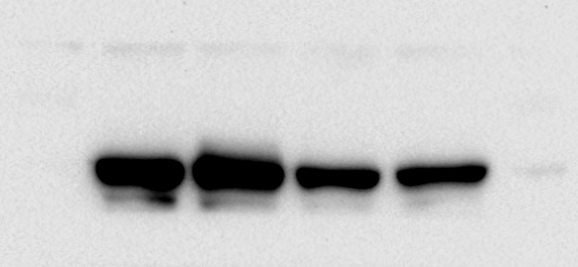

Supplement: Figure 1—figure supplement 1—source data 2. [file elife-82324-fig1-figsupp1-data2.zip › Figure 1-figure supplement 1-souce data 2/Figure 1S1B Repeat2/Chk1.tif]

Figure 1-figure supplement 1C

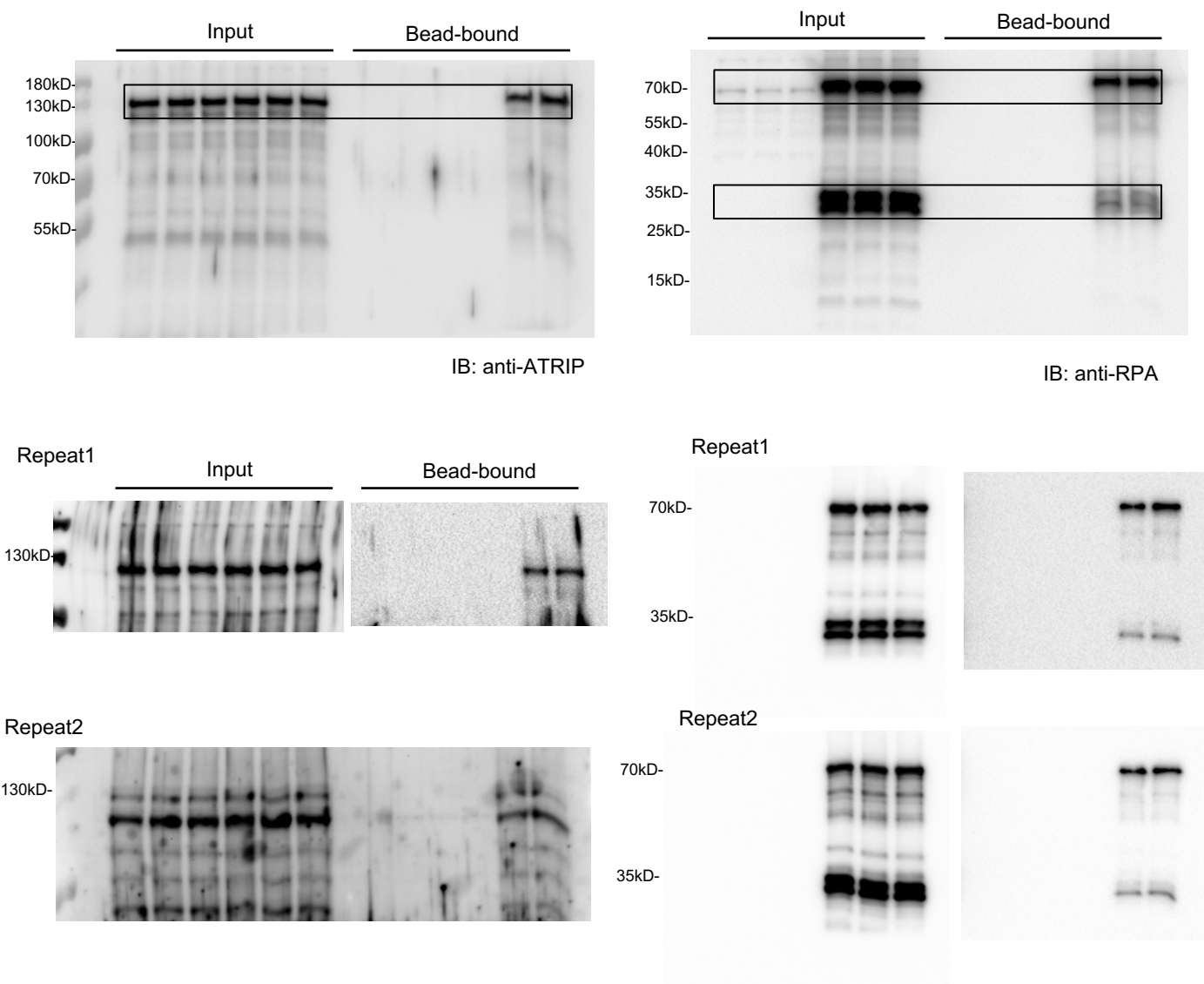

Supplement: Figure 1—figure supplement 1—source data 3. [file elife-82324-fig1-figsupp1-data3.zip › Figure 1-figure supplement 1-souce data 3/IB-data-Figure 1S1C.pdf]

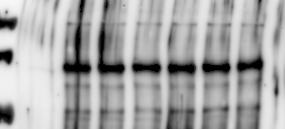

Supplement: Figure 1—figure supplement 1—source data 3. [file elife-82324-fig1-figsupp1-data3.zip › Figure 1-figure supplement 1-souce data 3/Figure 1S1C Repeat1/Input-ATRIP.tif]

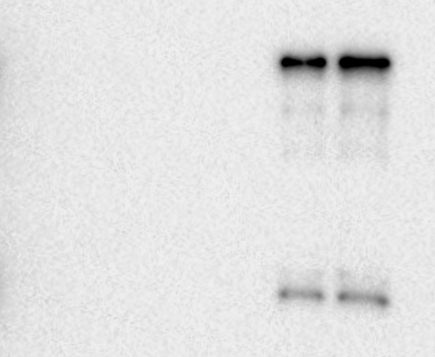

Supplement: Figure 1—figure supplement 1—source data 3. [file elife-82324-fig1-figsupp1-data3.zip › Figure 1-figure supplement 1-souce data 3/Figure 1S1C Repeat1/Bead-bound-RPA.tif]

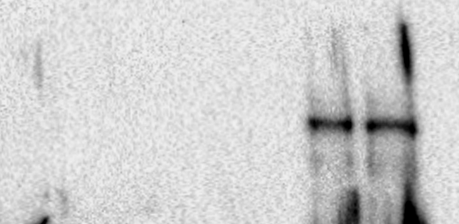

Supplement: Figure 1—figure supplement 1—source data 3. [file elife-82324-fig1-figsupp1-data3.zip › Figure 1-figure supplement 1-souce data 3/Figure 1S1C Repeat1/Bead-bound-ATRIP.tif]

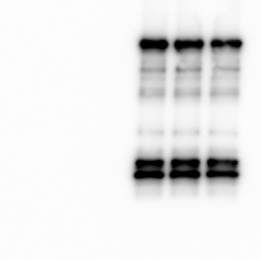

Supplement: Figure 1—figure supplement 1—source data 3. [file elife-82324-fig1-figsupp1-data3.zip › Figure 1-figure supplement 1-souce data 3/Figure 1S1C Repeat1/Input-RPA.tif]

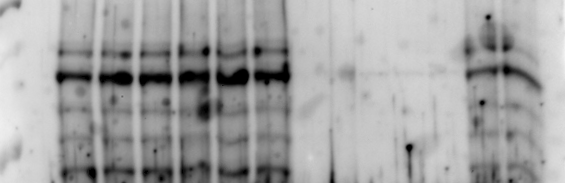

Supplement: Figure 1—figure supplement 1—source data 3. [file elife-82324-fig1-figsupp1-data3.zip › Figure 1-figure supplement 1-souce data 3/Figure 1S1C Repeat2/Input&Bead-bound-ATRIP.tif]

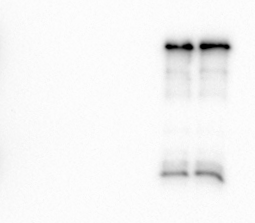

Supplement: Figure 1—figure supplement 1—source data 3. [file elife-82324-fig1-figsupp1-data3.zip › Figure 1-figure supplement 1-souce data 3/Figure 1S1C Repeat2/Bead-bound-RPA.tif]

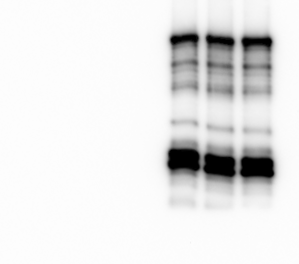

Supplement: Figure 1—figure supplement 1—source data 3. [file elife-82324-fig1-figsupp1-data3.zip › Figure 1-figure supplement 1-souce data 3/Figure 1S1C Repeat2/Input-RPA.tif]

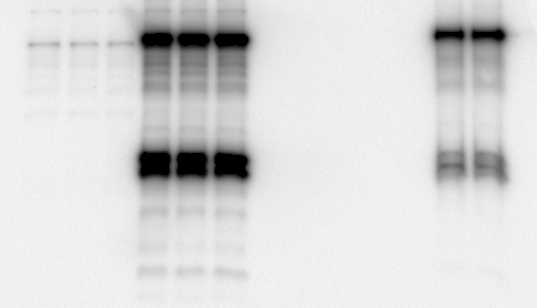

Supplement: Figure 1—figure supplement 1—source data 3. [file elife-82324-fig1-figsupp1-data3.zip › Figure 1-figure supplement 1-souce data 3/Figure 1S1C initial trial/Input&Bead-bound-RPA.tif]

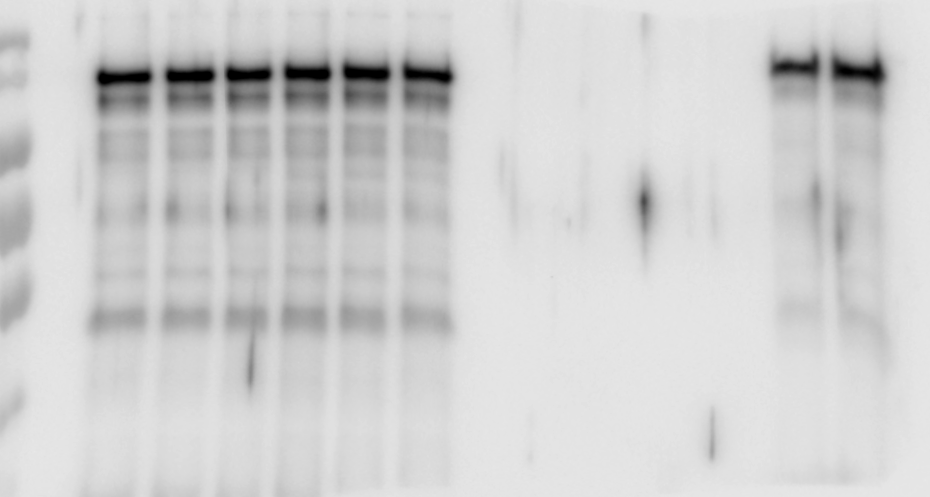

Supplement: Figure 1—figure supplement 1—source data 3. [file elife-82324-fig1-figsupp1-data3.zip › Figure 1-figure supplement 1-souce data 3/Figure 1S1C initial trial/Input&Bead-bound-ATRIP.tif]

Figure 1-figure supplement 1D

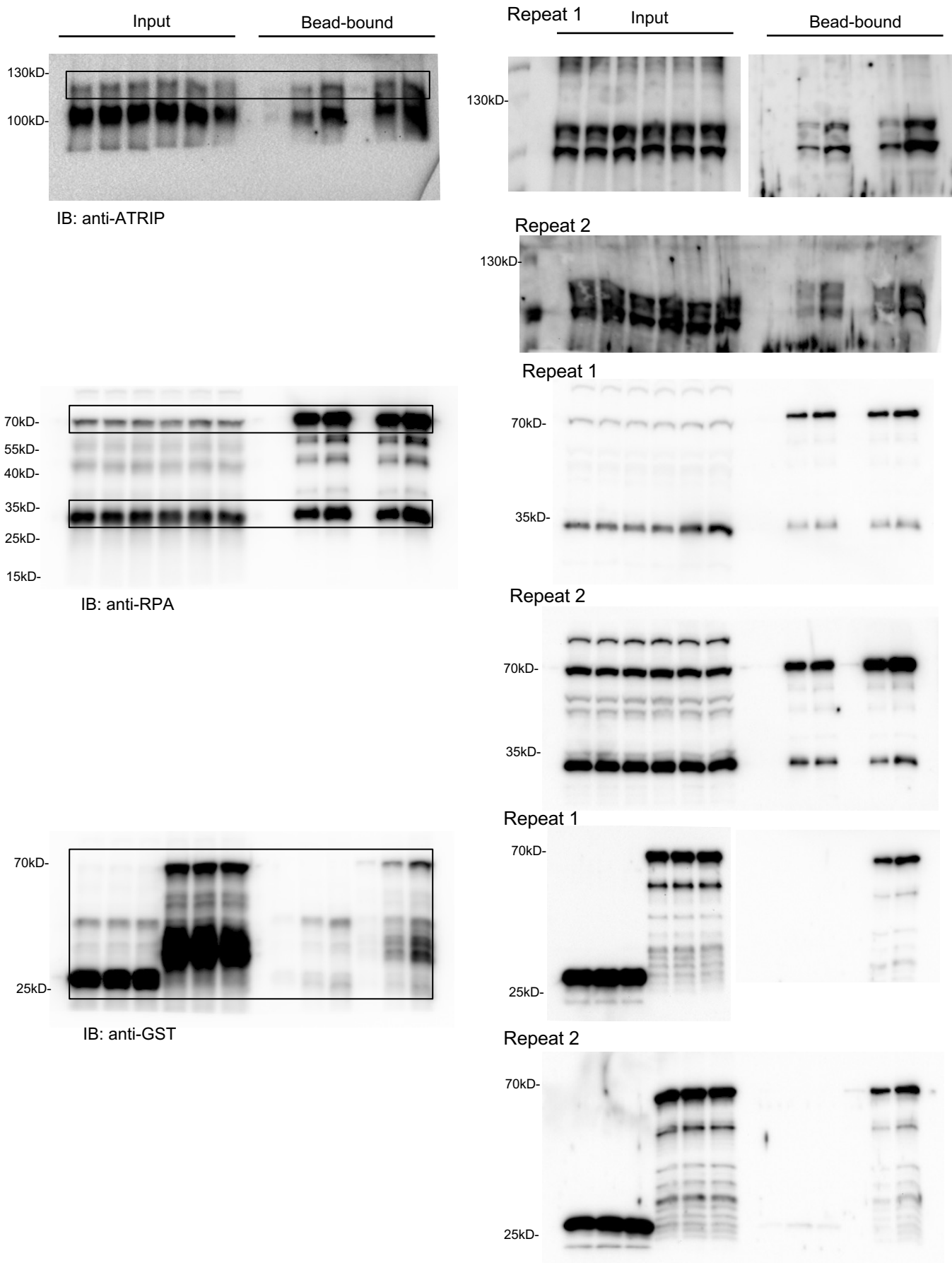

Supplement: Figure 1—figure supplement 1—source data 4. [file elife-82324-fig1-figsupp1-data4.zip › Figure 1-figure supplement 1-souce data 4/IB-data-Figure 1S1D.pdf]

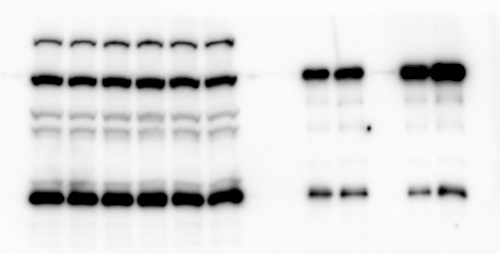

Supplement: Figure 1—figure supplement 1—source data 4. [file elife-82324-fig1-figsupp1-data4.zip › Figure 1-figure supplement 1-souce data 4/Figure 1S1D Repeat2/input and bead-bound-RPA.tif]

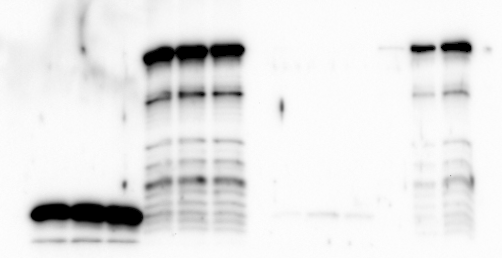

Supplement: Figure 1—figure supplement 1—source data 4. [file elife-82324-fig1-figsupp1-data4.zip › Figure 1-figure supplement 1-souce data 4/Figure 1S1D Repeat2/input and bead-bound-GST.tif]

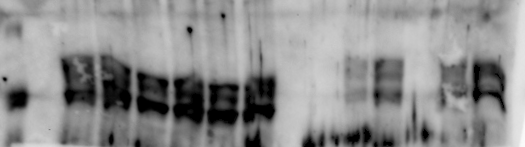

Supplement: Figure 1—figure supplement 1—source data 4. [file elife-82324-fig1-figsupp1-data4.zip › Figure 1-figure supplement 1-souce data 4/Figure 1S1D Repeat2/input and bead-bound-ATRIP.tif]

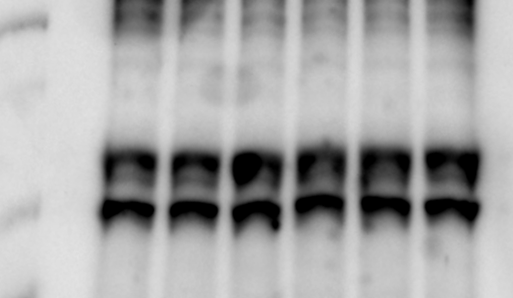

Supplement: Figure 1—figure supplement 1—source data 4. [file elife-82324-fig1-figsupp1-data4.zip › Figure 1-figure supplement 1-souce data 4/Figure 1S1D Repeat1/Inpu-ATRIP.tif]

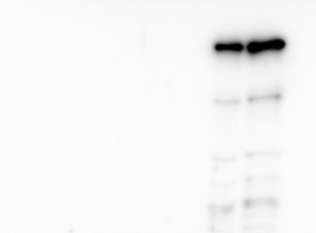

Supplement: Figure 1—figure supplement 1—source data 4. [file elife-82324-fig1-figsupp1-data4.zip › Figure 1-figure supplement 1-souce data 4/Figure 1S1D Repeat1/Bead-bound-GST.tif]

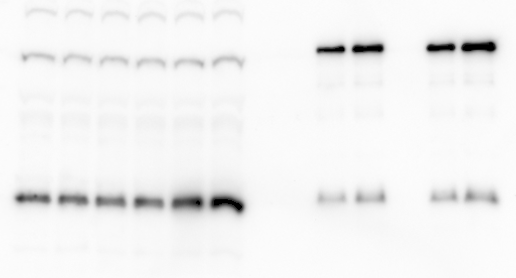

Supplement: Figure 1—figure supplement 1—source data 4. [file elife-82324-fig1-figsupp1-data4.zip › Figure 1-figure supplement 1-souce data 4/Figure 1S1D Repeat1/Input and bead-bound-RPA.tif]

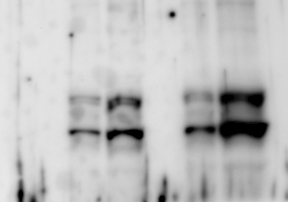

Supplement: Figure 1—figure supplement 1—source data 4. [file elife-82324-fig1-figsupp1-data4.zip › Figure 1-figure supplement 1-souce data 4/Figure 1S1D Repeat1/Bead-bound-ATRIP.tif]

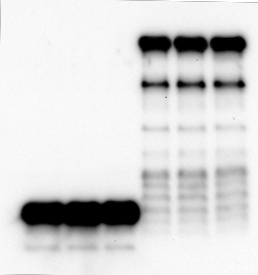

Supplement: Figure 1—figure supplement 1—source data 4. [file elife-82324-fig1-figsupp1-data4.zip › Figure 1-figure supplement 1-souce data 4/Figure 1S1D Repeat1/Input-GST.tif]

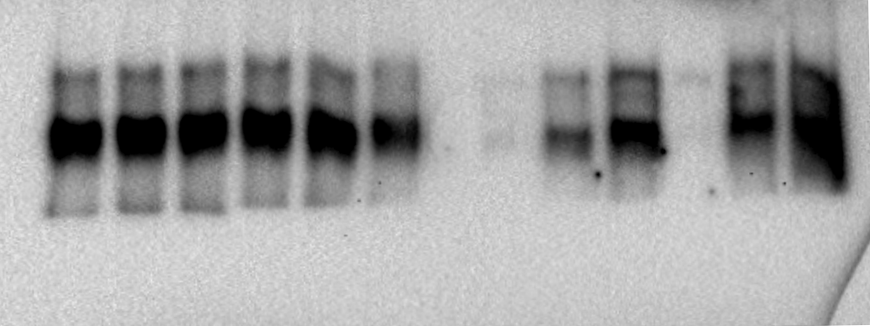

Supplement: Figure 1—figure supplement 1—source data 4. [file elife-82324-fig1-figsupp1-data4.zip › Figure 1-figure supplement 1-souce data 4/Figure 1S1D initial trial/ATRIP.tif]

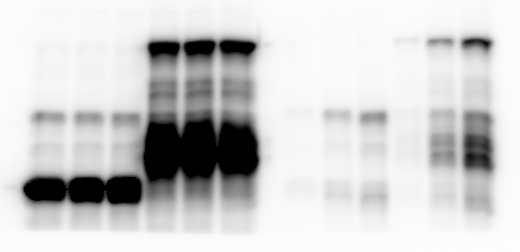

Supplement: Figure 1—figure supplement 1—source data 4. [file elife-82324-fig1-figsupp1-data4.zip › Figure 1-figure supplement 1-souce data 4/Figure 1S1D initial trial/GST.tif]

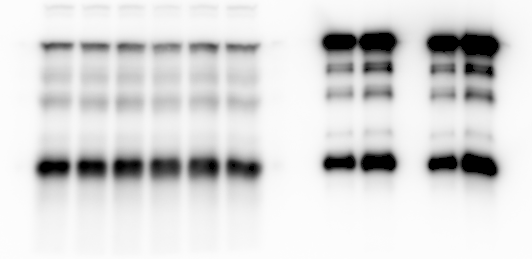

Supplement: Figure 1—figure supplement 1—source data 4. [file elife-82324-fig1-figsupp1-data4.zip › Figure 1-figure supplement 1-souce data 4/Figure 1S1D initial trial/RPA.tif]

Figure 2B

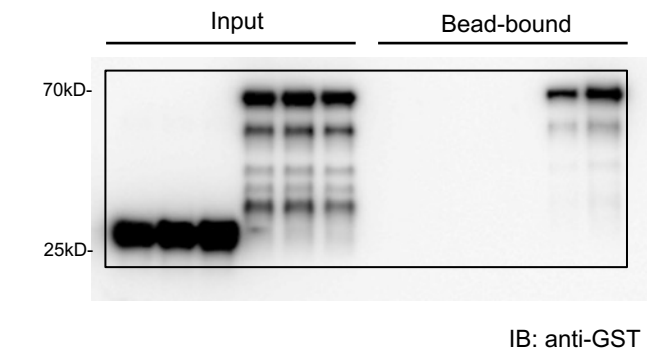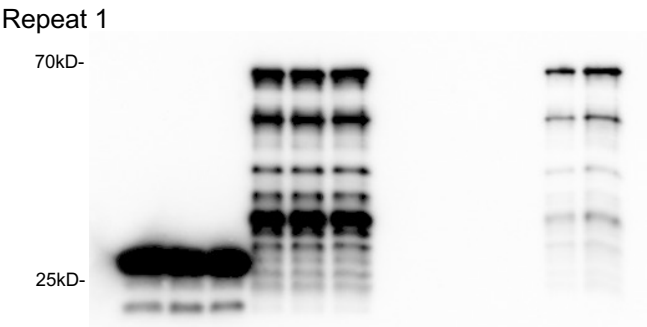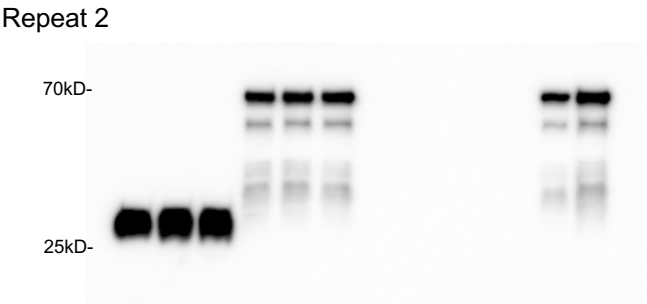

Supplement: Figure 2—source data 1. [file elife-82324-fig2-data1.zip › Figure 2-source data 1/IB-data-Figure 2B.pdf]

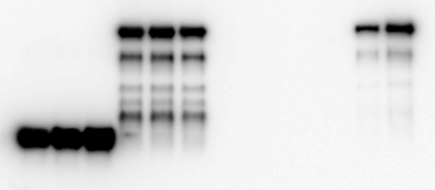

Supplement: Figure 2—source data 1. [file elife-82324-fig2-data1.zip › Figure 2-source data 1/Figure 2B initial trial/Input and Bead-bound-GST.tif]

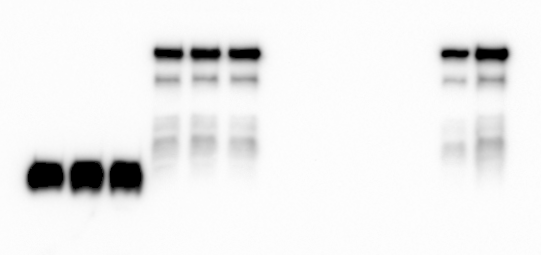

Supplement: Figure 2—source data 1. [file elife-82324-fig2-data1.zip › Figure 2-source data 1/Figure 2B Repeat2/Input and Bead-bound-GST.tif]

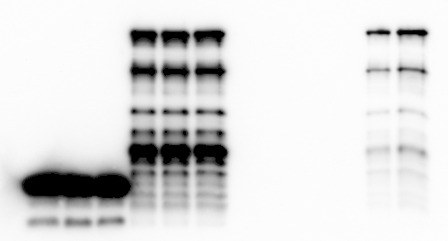

Supplement: Figure 2—source data 1. [file elife-82324-fig2-data1.zip › Figure 2-source data 1/Figure 2B Repeat1/Input and Bead-bound-GST.tif]

Figure 2C

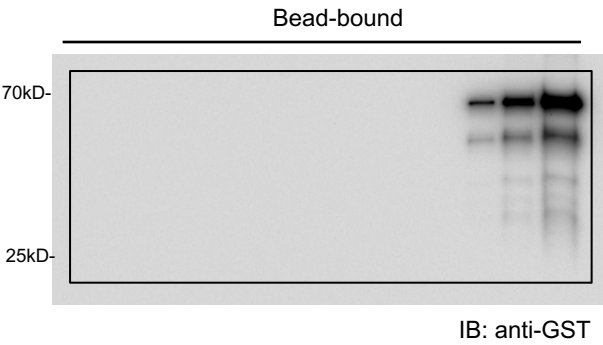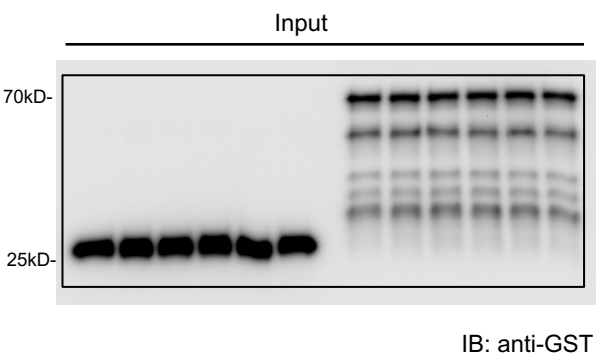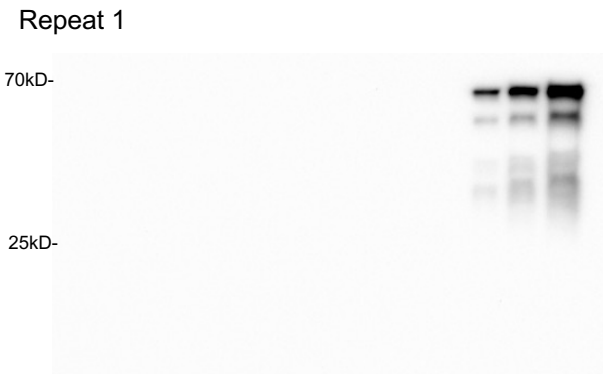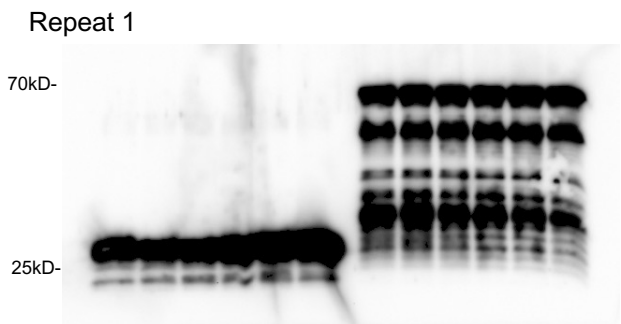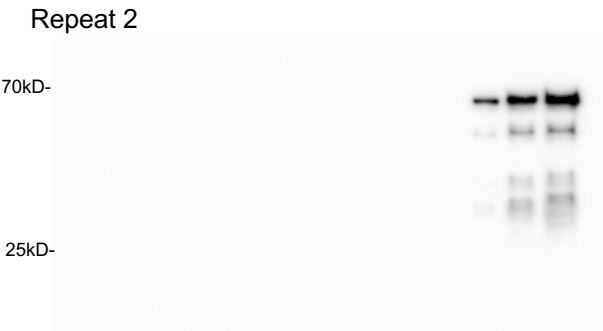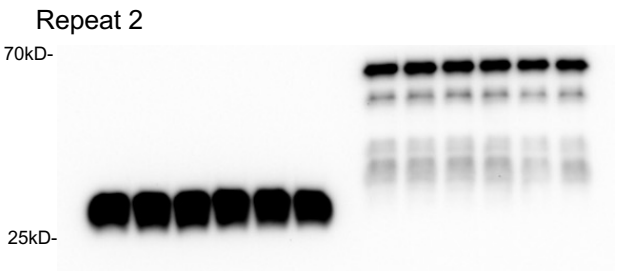

Supplement: Figure 2—source data 2. [file elife-82324-fig2-data2.zip › Figure 2-source data 2/IB-data-Figure 2C.pdf]

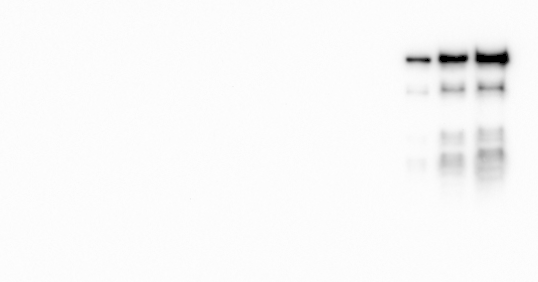

Supplement: Figure 2—source data 2. [file elife-82324-fig2-data2.zip › Figure 2-source data 2/Figure 2C Repeat2/Bead-bound-GST.tif]

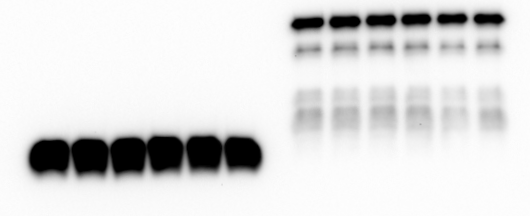

Supplement: Figure 2—source data 2. [file elife-82324-fig2-data2.zip › Figure 2-source data 2/Figure 2C Repeat2/Input-GST.tif]

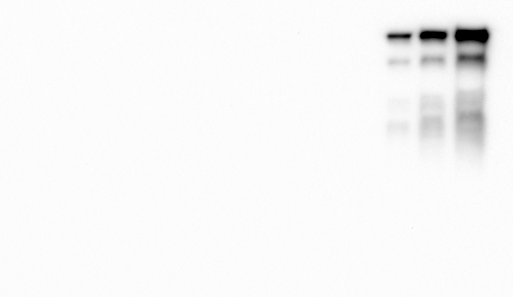

Supplement: Figure 2—source data 2. [file elife-82324-fig2-data2.zip › Figure 2-source data 2/Figure 2C Repeat1/Bead-bound-GST.tif]

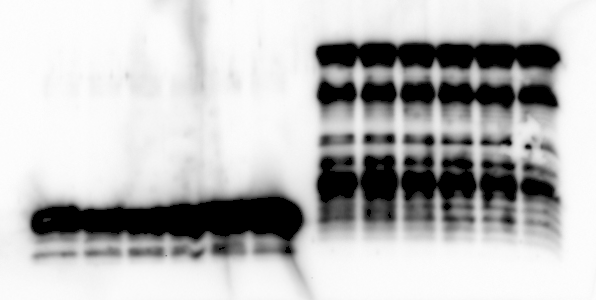

Supplement: Figure 2—source data 2. [file elife-82324-fig2-data2.zip › Figure 2-source data 2/Figure 2C Repeat1/Input-GST.tif]

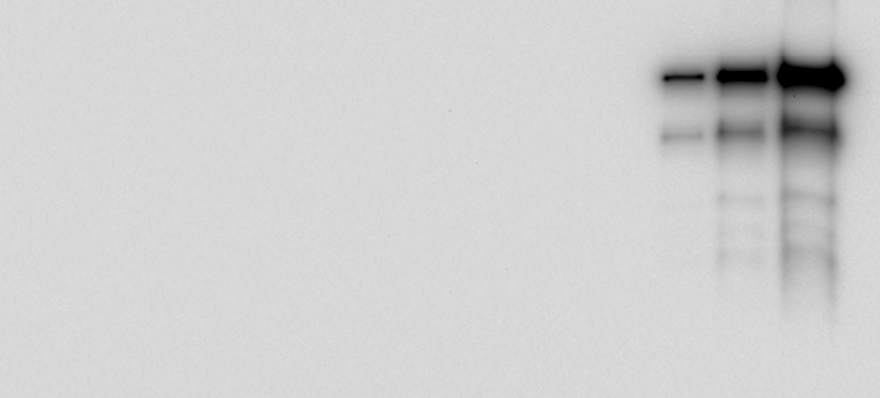

Supplement: Figure 2—source data 2. [file elife-82324-fig2-data2.zip › Figure 2-source data 2/Figure 2C initial trial/Bead-bound-GST.tif]

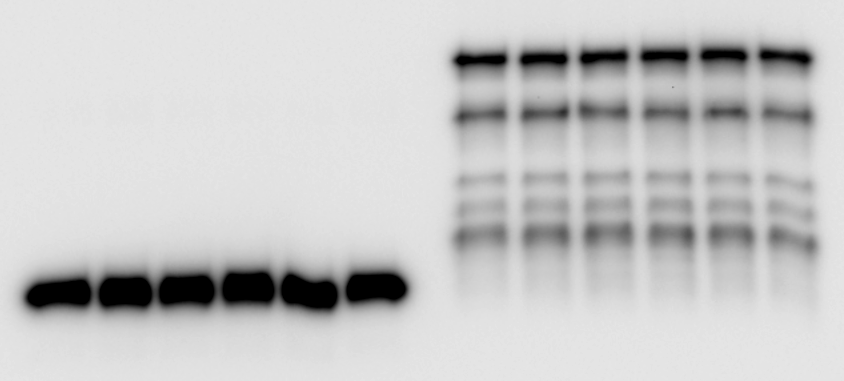

Supplement: Figure 2—source data 2. [file elife-82324-fig2-data2.zip › Figure 2-source data 2/Figure 2C initial trial/Input-GST.tif]

Figure 2D

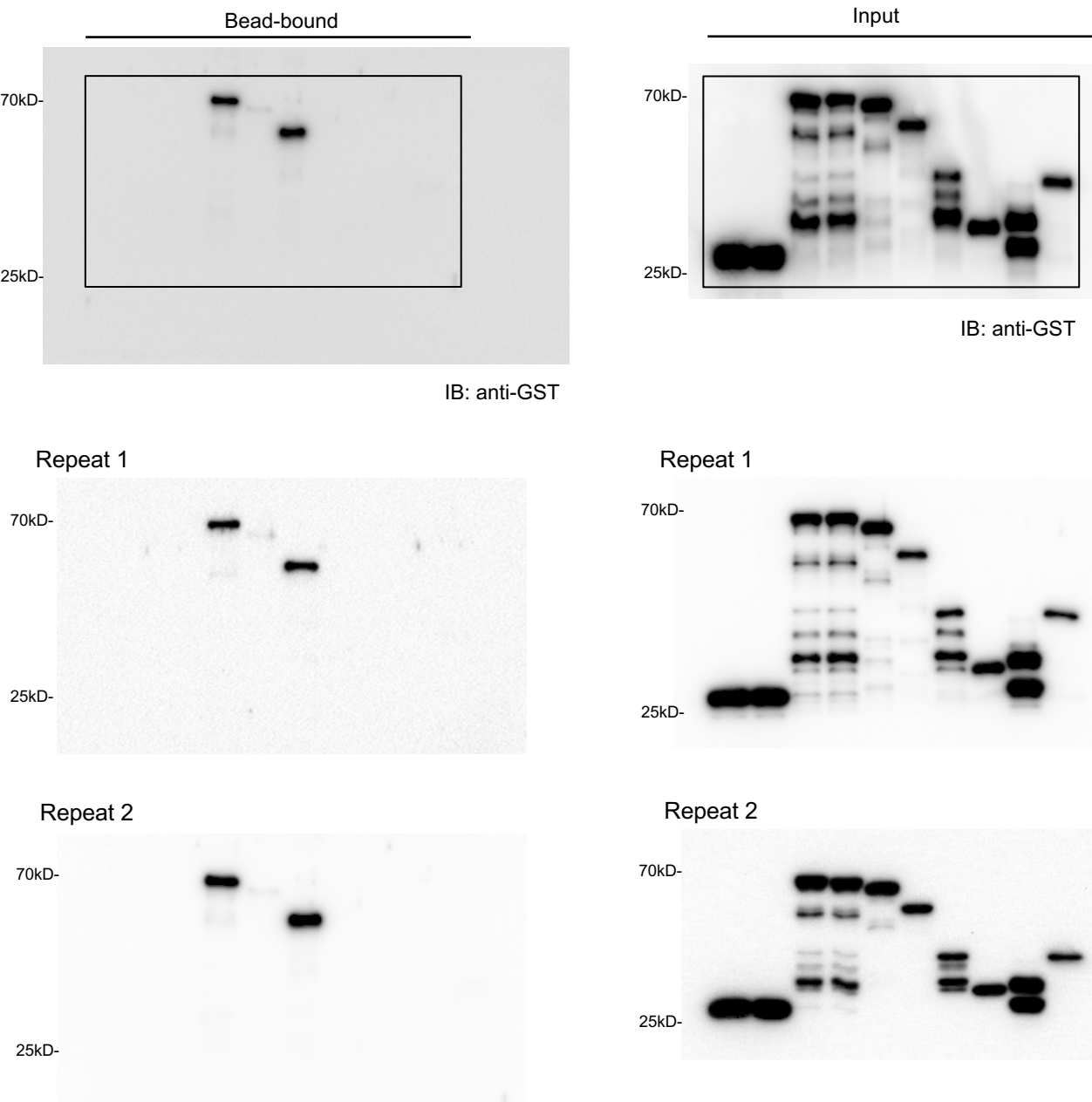

Supplement: Figure 2—source data 3. [file elife-82324-fig2-data3.zip › Figure 2-source data 3/IB-data-Figure 2D.pdf]

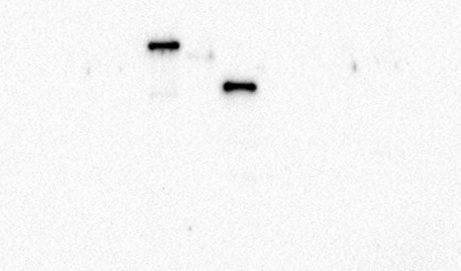

Supplement: Figure 2—source data 3. [file elife-82324-fig2-data3.zip › Figure 2-source data 3/Figure 2D Repeat1/Bead-bound-GST.tif]

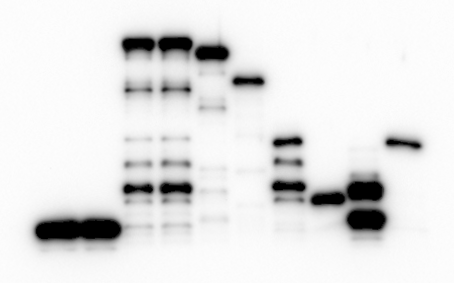

Supplement: Figure 2—source data 3. [file elife-82324-fig2-data3.zip › Figure 2-source data 3/Figure 2D Repeat1/Input-GST.tif]

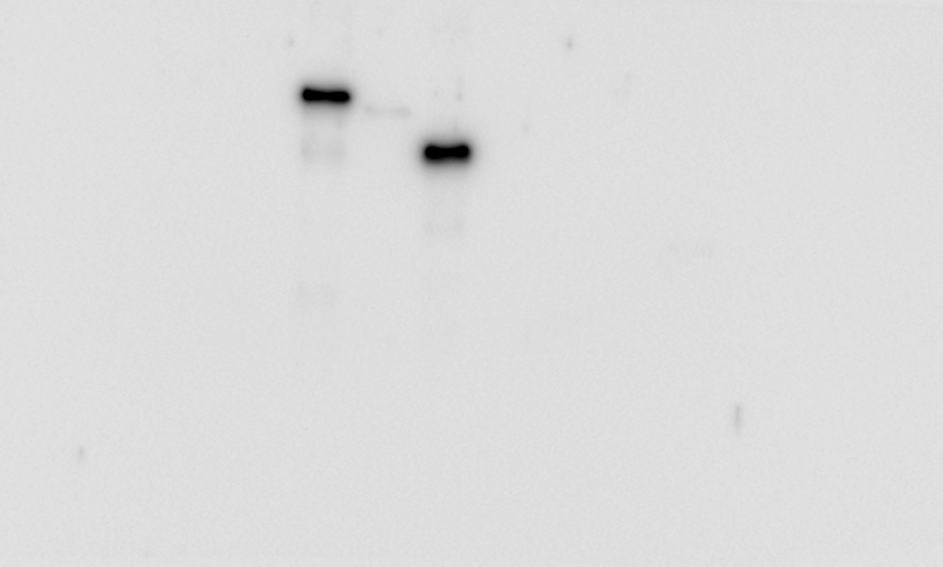

Supplement: Figure 2—source data 3. [file elife-82324-fig2-data3.zip › Figure 2-source data 3/Figure 2D initial trial/Bed-bound-GST.tif]

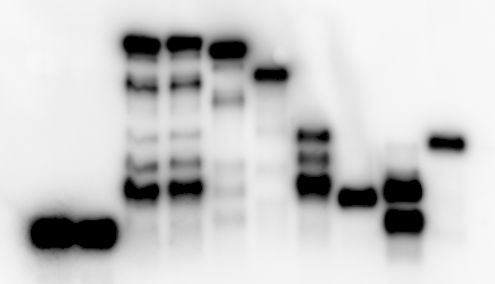

Supplement: Figure 2—source data 3. [file elife-82324-fig2-data3.zip › Figure 2-source data 3/Figure 2D initial trial/Input-GST.tif]

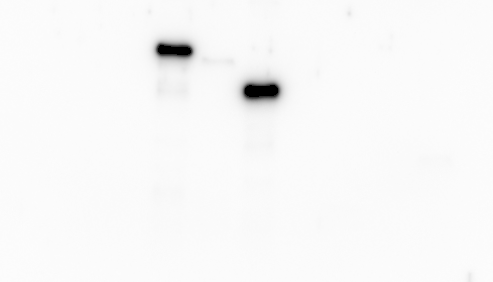

Supplement: Figure 2—source data 3. [file elife-82324-fig2-data3.zip › Figure 2-source data 3/Figure 2D Repeat2/Bead-bound-GST.tif]

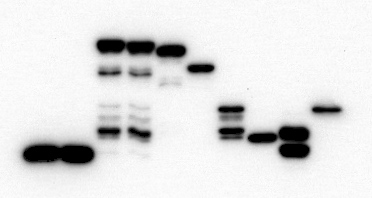

Supplement: Figure 2—source data 3. [file elife-82324-fig2-data3.zip › Figure 2-source data 3/Figure 2D Repeat2/Input-GST.tif]

Figure 2-figure supplement 1

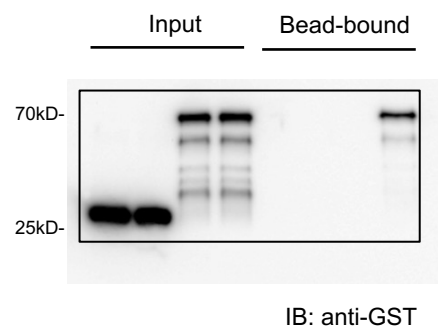

Repeat 1

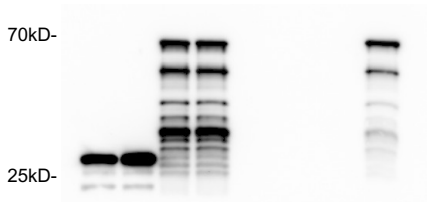

Repeat 2

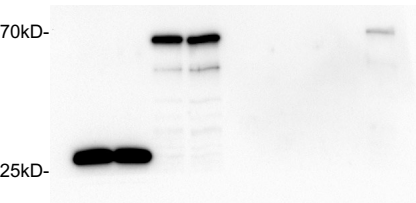

Supplement: Figure 2—figure supplement 1—source data 1. [file elife-82324-fig2-figsupp1-data1.zip › Figure 2-figure supplement 1-souce data 1/IB-data-Figure 2S1.pdf]

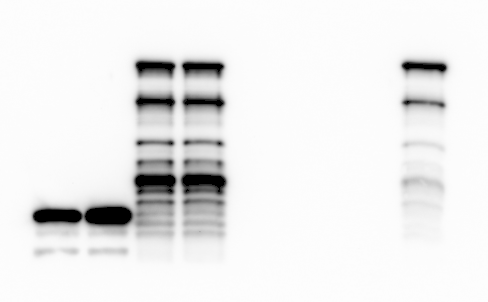

Supplement: Figure 2—figure supplement 1—source data 1. [file elife-82324-fig2-figsupp1-data1.zip › Figure 2-figure supplement 1-souce data 1/Figure 2S1 Repeat1/input-bead-GST.tif]

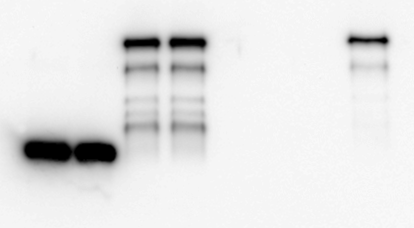

Supplement: Figure 2—figure supplement 1—source data 1. [file elife-82324-fig2-figsupp1-data1.zip › Figure 2-figure supplement 1-souce data 1/Figure 2S1 initial trial/input-bead-GST.tif]

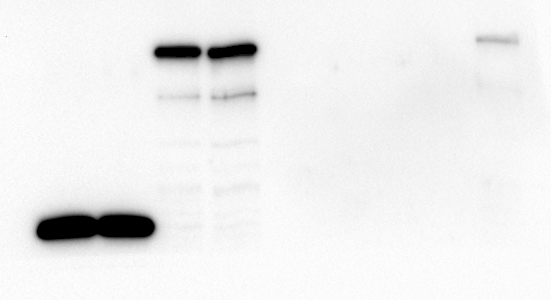

Supplement: Figure 2—figure supplement 1—source data 1. [file elife-82324-fig2-figsupp1-data1.zip › Figure 2-figure supplement 1-souce data 1/Figure 2S1 Repeat2/Input-bead-GST.tif]

Figure 3A

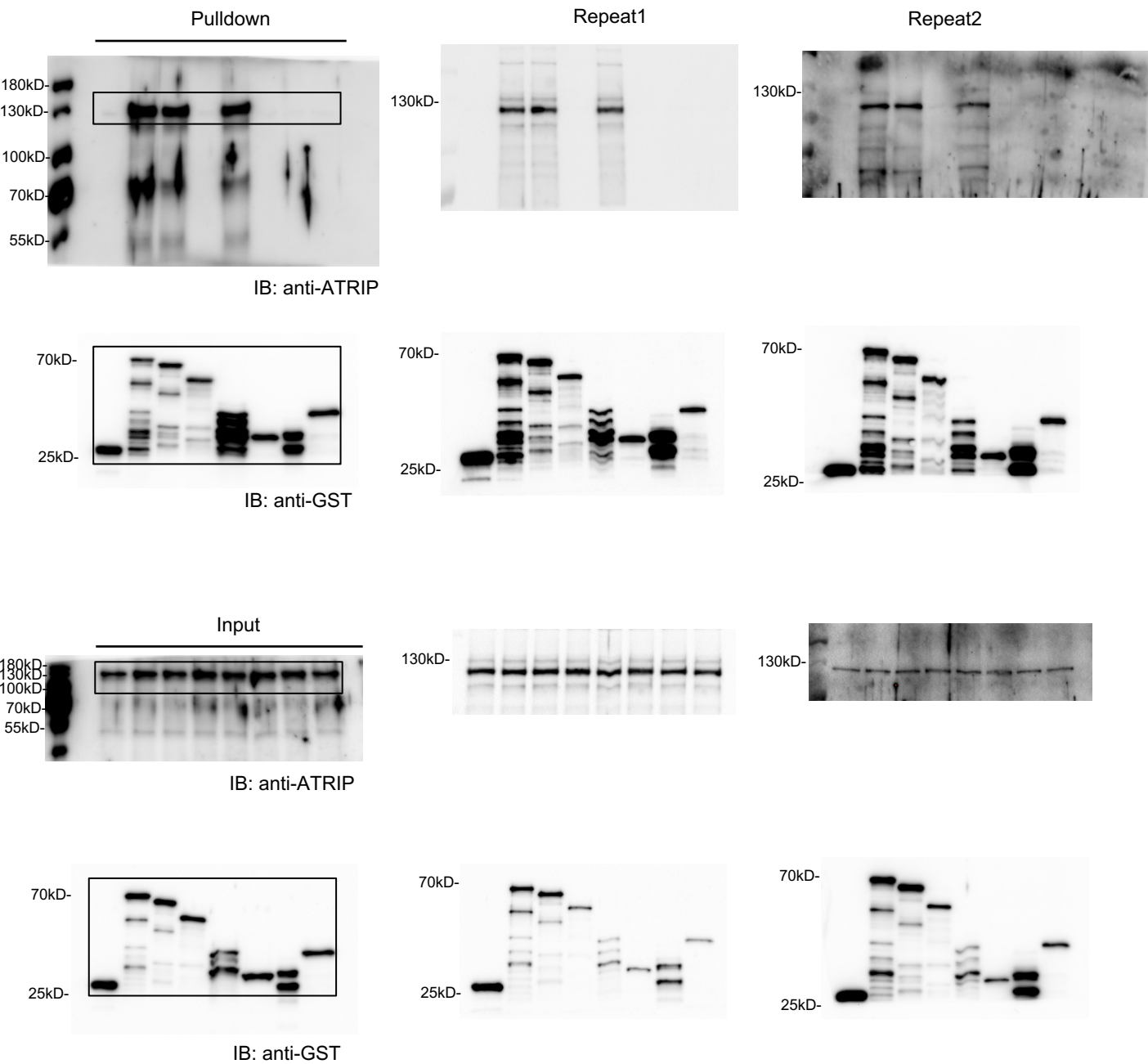

Supplement: Figure 3—source data 1. [file elife-82324-fig3-data1.zip › Figure 3-source data 1/IB-data-Figure 3A.pdf]

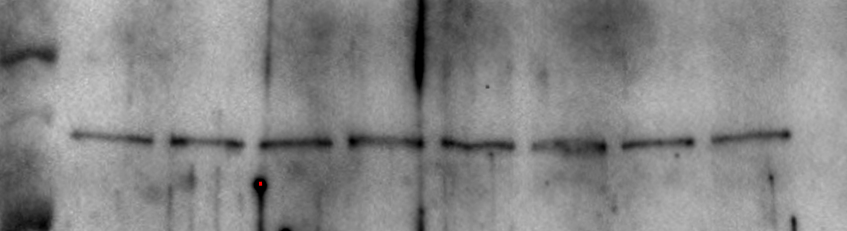

Supplement: Figure 3—source data 1. [file elife-82324-fig3-data1.zip › Figure 3-source data 1/Figure 3A Repeat2/Input-ATRIP.tif]

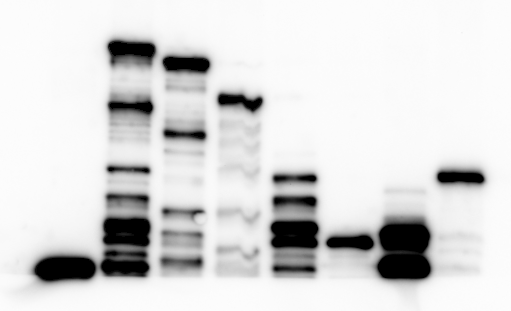

Supplement: Figure 3—source data 1. [file elife-82324-fig3-data1.zip › Figure 3-source data 1/Figure 3A Repeat2/Pulldown-GST.tif]

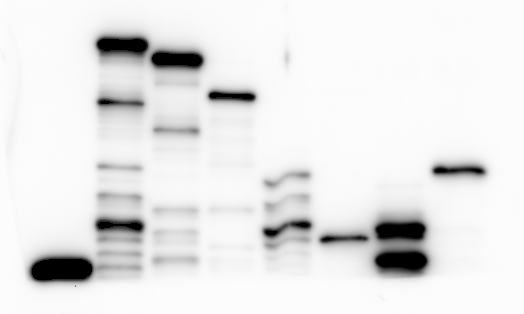

Supplement: Figure 3—source data 1. [file elife-82324-fig3-data1.zip › Figure 3-source data 1/Figure 3A Repeat2/Input-GST.tif]

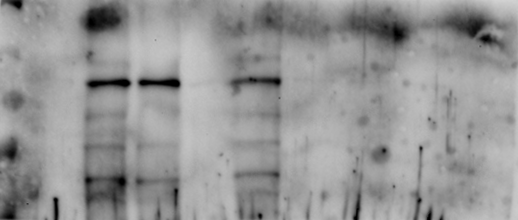

Supplement: Figure 3—source data 1. [file elife-82324-fig3-data1.zip › Figure 3-source data 1/Figure 3A Repeat2/Pulldown-ATRIP.tif]

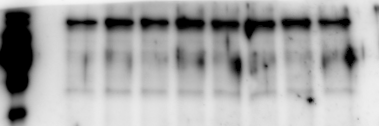

Supplement: Figure 3—source data 1. [file elife-82324-fig3-data1.zip › Figure 3-source data 1/Figure 3A initial trial/Input-ATRIP.tif]

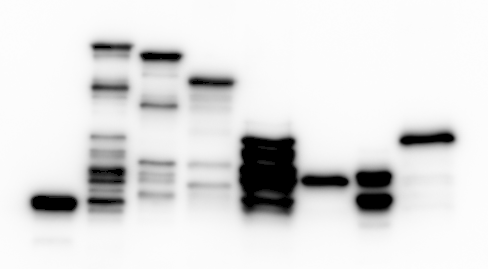

Supplement: Figure 3—source data 1. [file elife-82324-fig3-data1.zip › Figure 3-source data 1/Figure 3A initial trial/Pulldown-GST.tif]

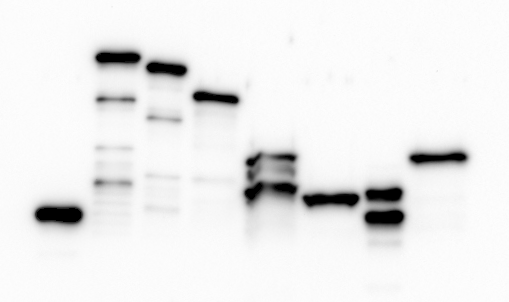

Supplement: Figure 3—source data 1. [file elife-82324-fig3-data1.zip › Figure 3-source data 1/Figure 3A initial trial/Input-GST.tif]

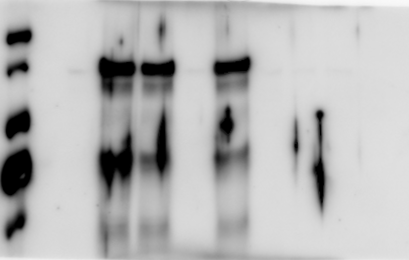

Supplement: Figure 3—source data 1. [file elife-82324-fig3-data1.zip › Figure 3-source data 1/Figure 3A initial trial/Pulldown-ATRIP.tif]

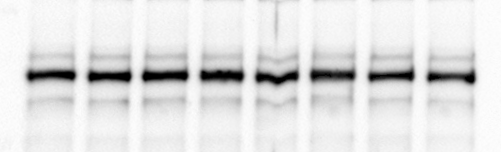

Supplement: Figure 3—source data 1. [file elife-82324-fig3-data1.zip › Figure 3-source data 1/Figure 3A Repeat1/Input-ATRIP.tif]

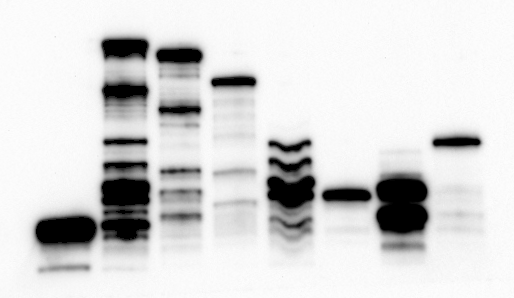

Supplement: Figure 3—source data 1. [file elife-82324-fig3-data1.zip › Figure 3-source data 1/Figure 3A Repeat1/Pulldown-GST.tif]

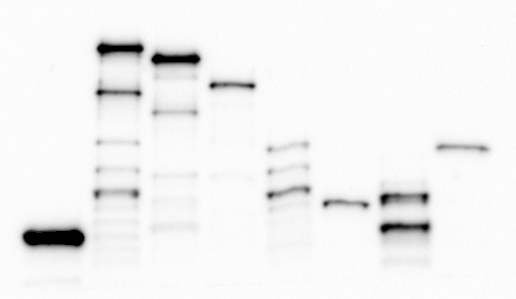

Supplement: Figure 3—source data 1. [file elife-82324-fig3-data1.zip › Figure 3-source data 1/Figure 3A Repeat1/Input-GST.tif]

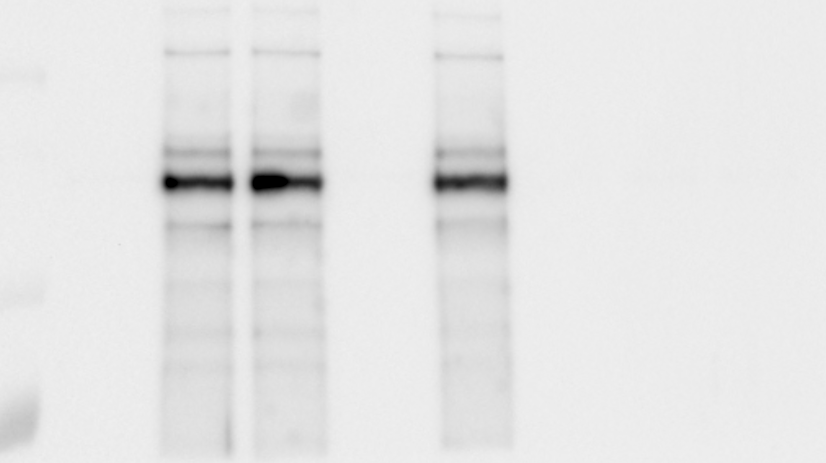

Supplement: Figure 3—source data 1. [file elife-82324-fig3-data1.zip › Figure 3-source data 1/Figure 3A Repeat1/Pulldown-ATRIP.tif]

Figure 3B

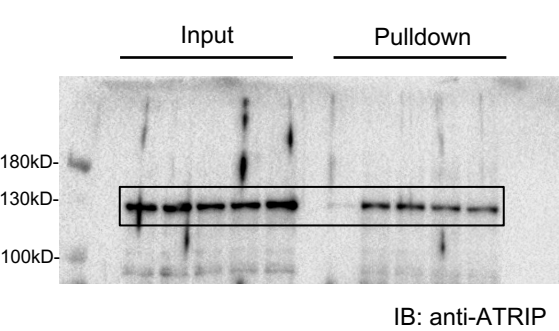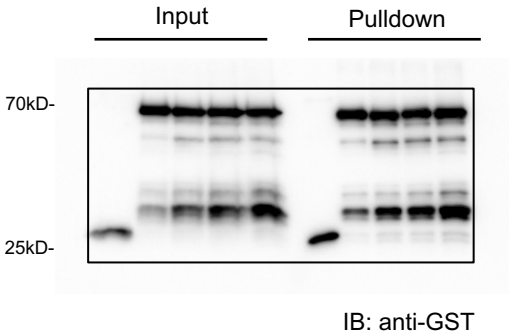

Repeat 1

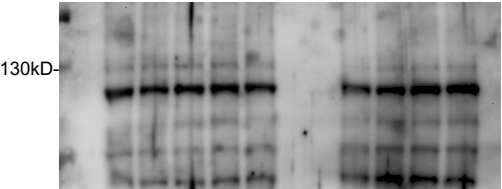

Repeat 1

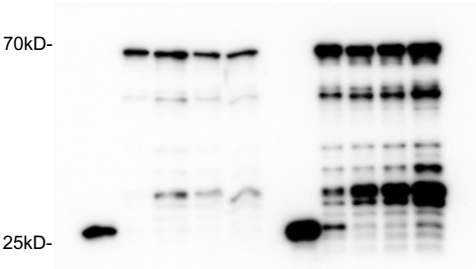

Repeat 2

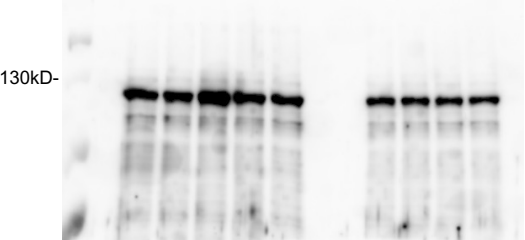

Repeat 2

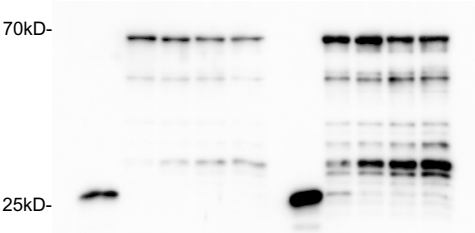

Supplement: Figure 3—source data 2. [file elife-82324-fig3-data2.zip › Figure 3-source data 2/IB-data-Figure 3B.pdf]

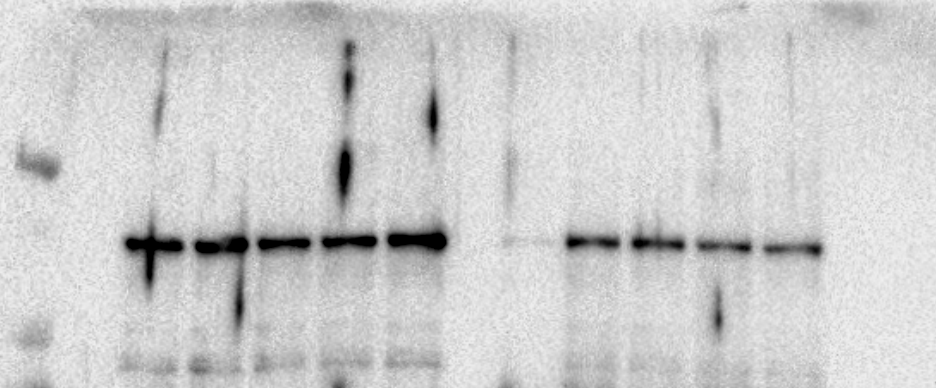

Supplement: Figure 3—source data 2. [file elife-82324-fig3-data2.zip › Figure 3-source data 2/Figure 3B initial trial/ATRIP.tif]

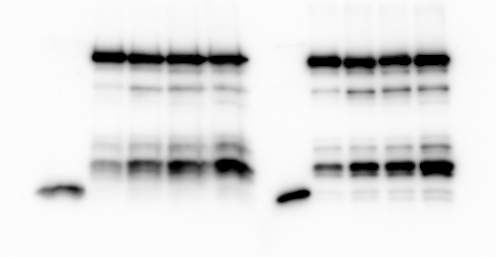

Supplement: Figure 3—source data 2. [file elife-82324-fig3-data2.zip › Figure 3-source data 2/Figure 3B initial trial/GST.tif]

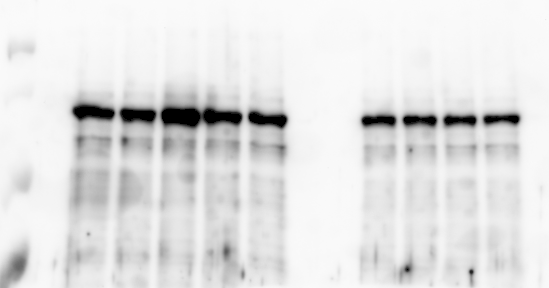

Supplement: Figure 3—source data 2. [file elife-82324-fig3-data2.zip › Figure 3-source data 2/Figure 3B Repeat2/ATRIP.tif]

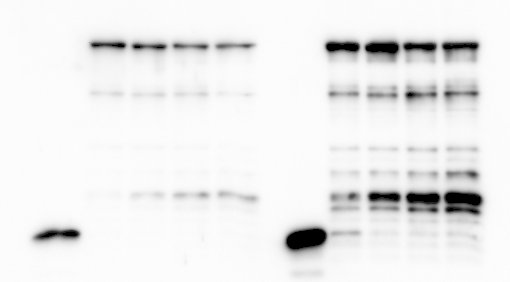

Supplement: Figure 3—source data 2. [file elife-82324-fig3-data2.zip › Figure 3-source data 2/Figure 3B Repeat2/GST.tif]

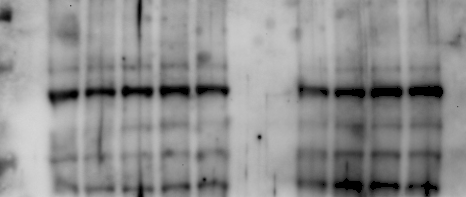

Supplement: Figure 3—source data 2. [file elife-82324-fig3-data2.zip › Figure 3-source data 2/Figure 3B Repeat1/ATRIP.tif]

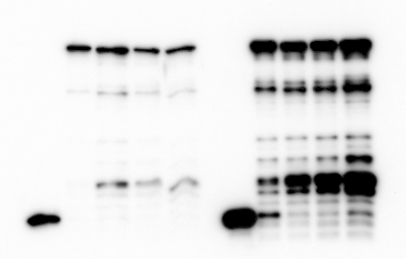

Supplement: Figure 3—source data 2. [file elife-82324-fig3-data2.zip › Figure 3-source data 2/Figure 3B Repeat1/GST.tif]

Figure 3C

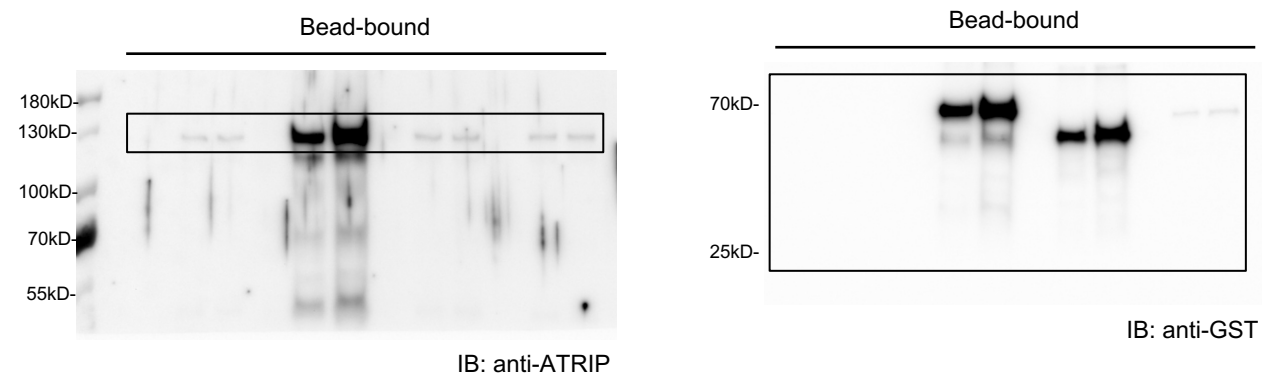

Repeat 1

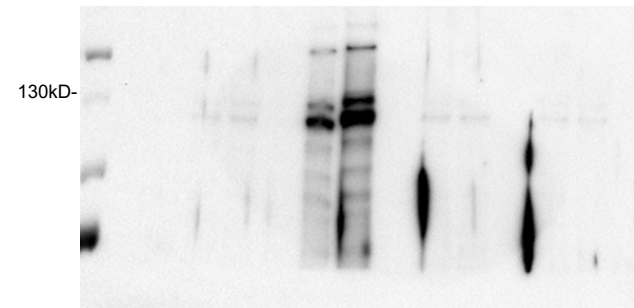

Repeat 1

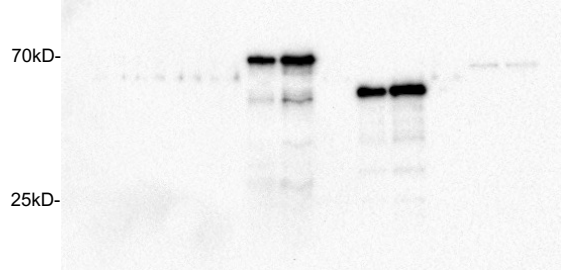

Repeat 2

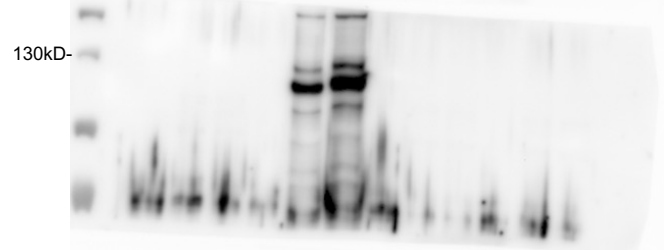

Repeat 2

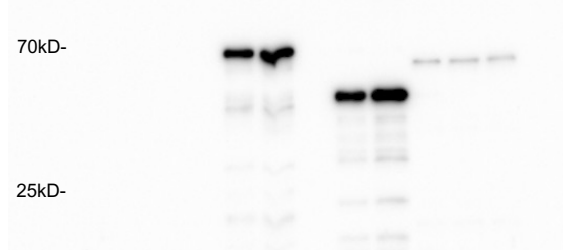

Figure 3C

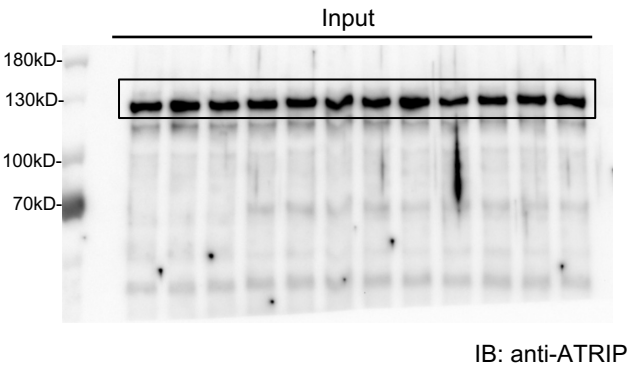

Repeat 1

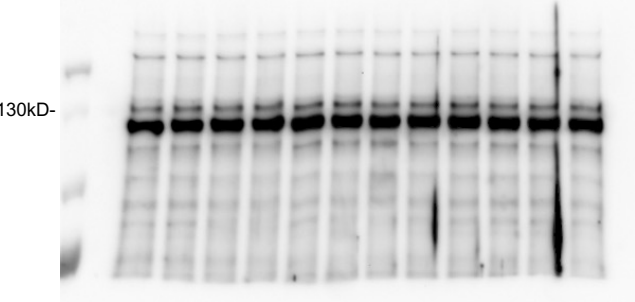

Repeat 2

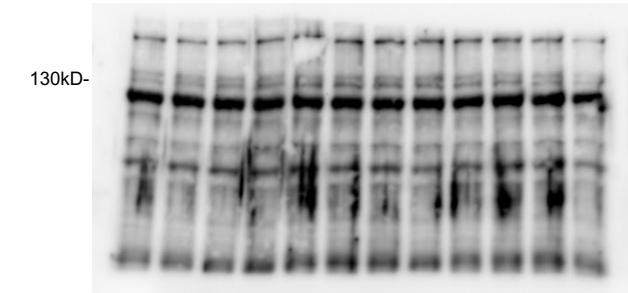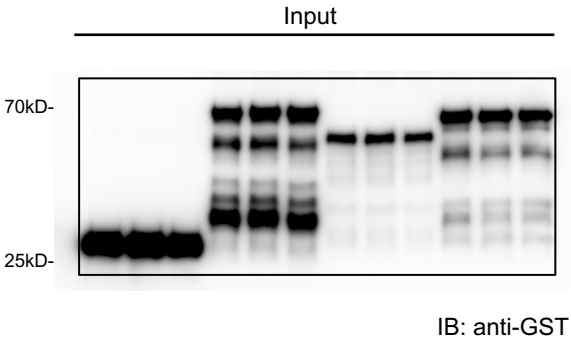

Repeat 1

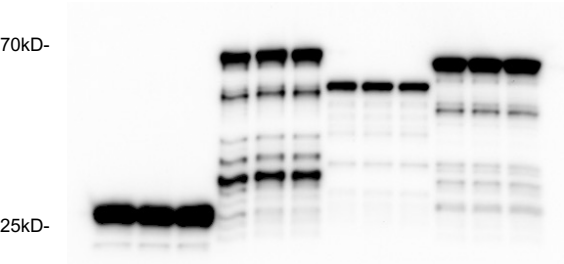

Repeat 2

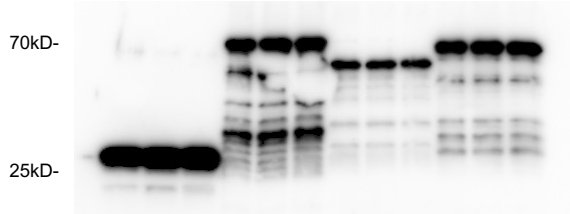

Supplement: Figure 3—source data 3. [file elife-82324-fig3-data3.zip › Figure 3-source data 3/IB-data-Figure 3C.pdf]

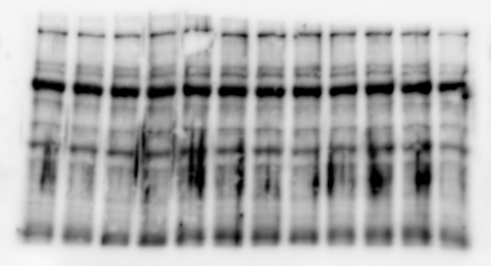

Supplement: Figure 3—source data 3. [file elife-82324-fig3-data3.zip › Figure 3-source data 3/Figure 3C Repeat2/Input-ATRIP.tif]

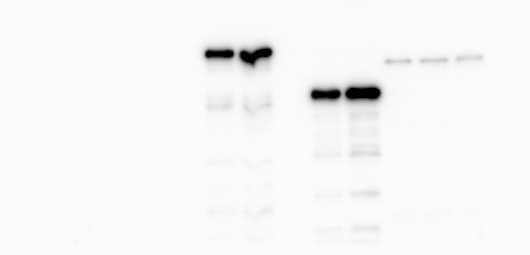

Supplement: Figure 3—source data 3. [file elife-82324-fig3-data3.zip › Figure 3-source data 3/Figure 3C Repeat2/Bead-GST.tif]

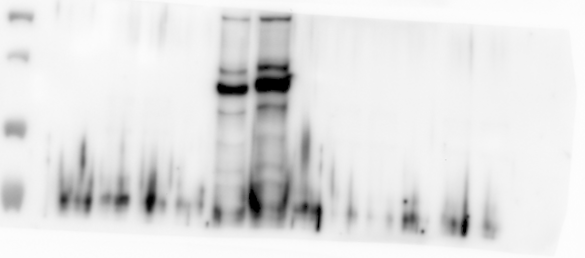

Supplement: Figure 3—source data 3. [file elife-82324-fig3-data3.zip › Figure 3-source data 3/Figure 3C Repeat2/Bead-ATRIP.tif]

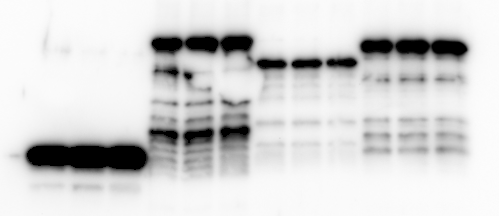

Supplement: Figure 3—source data 3. [file elife-82324-fig3-data3.zip › Figure 3-source data 3/Figure 3C Repeat2/Input-GST.tif]

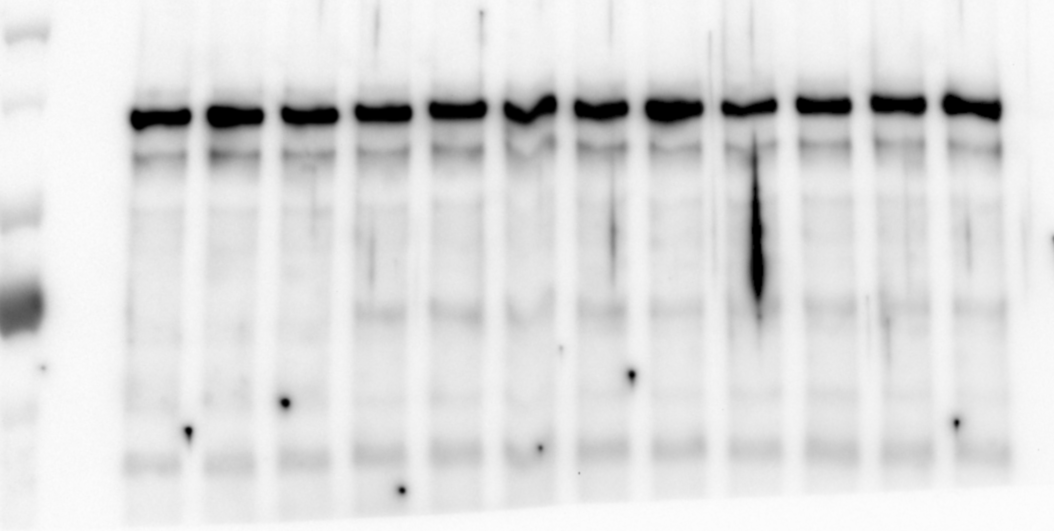

Supplement: Figure 3—source data 3. [file elife-82324-fig3-data3.zip › Figure 3-source data 3/Figure 3C initial trial/Input-ATRIP.tif]

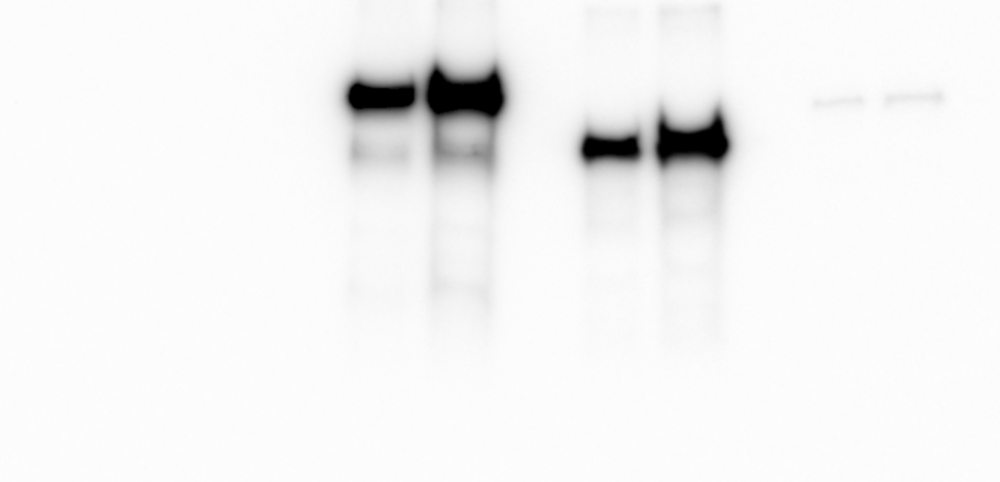

Supplement: Figure 3—source data 3. [file elife-82324-fig3-data3.zip › Figure 3-source data 3/Figure 3C initial trial/Bead-bound-GST.tif]

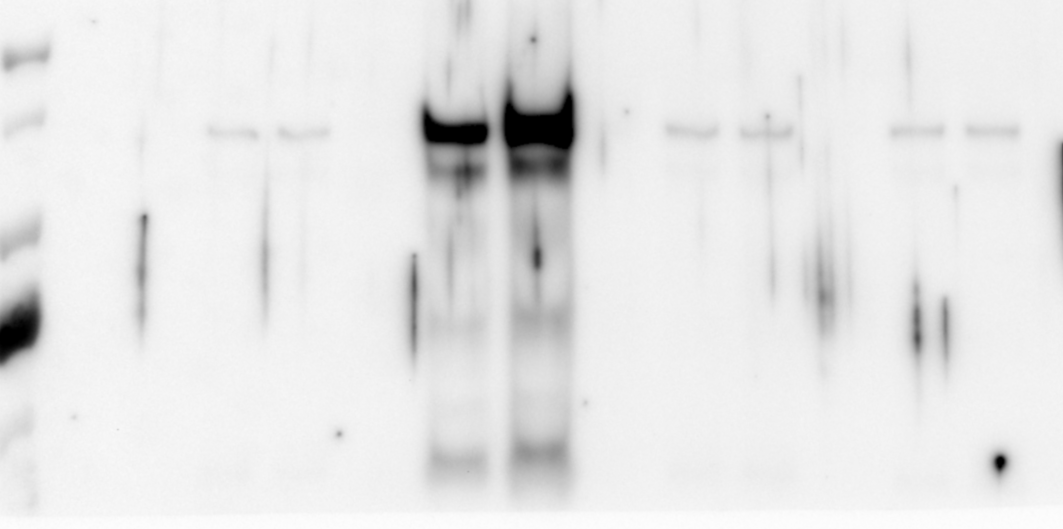

Supplement: Figure 3—source data 3. [file elife-82324-fig3-data3.zip › Figure 3-source data 3/Figure 3C initial trial/Bead-bound-ATRIP.tif]

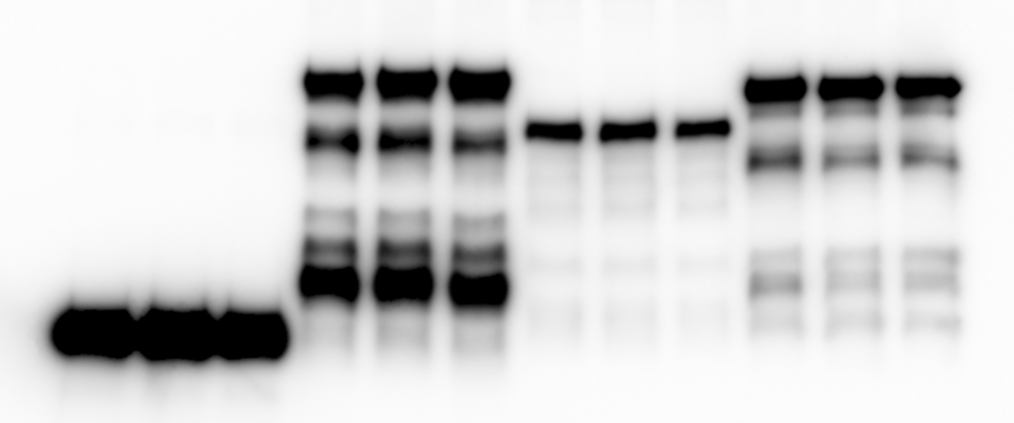

Supplement: Figure 3—source data 3. [file elife-82324-fig3-data3.zip › Figure 3-source data 3/Figure 3C initial trial/Input-GST.tif]

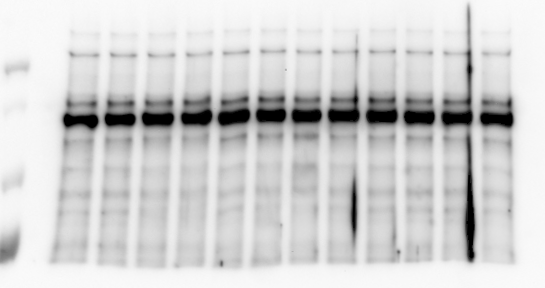

Supplement: Figure 3—source data 3. [file elife-82324-fig3-data3.zip › Figure 3-source data 3/Figure 3C Repeat1/Input-ATRIP.tif]

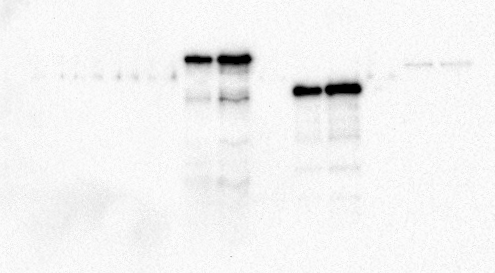

Supplement: Figure 3—source data 3. [file elife-82324-fig3-data3.zip › Figure 3-source data 3/Figure 3C Repeat1/Bead-bound-GST.tif]
