# Supplementary figures and images for: APE1 recruits ATRIP to ssDNA in an RPA-dependent and -independent manner to promote the ATR DNA damage response (part 3 of 4)
Source: eLife. 2023 May 22;12:e82324. doi: 10.7554/eLife.82324 (PMC10202453; doi:10.7554/eLife.82324)

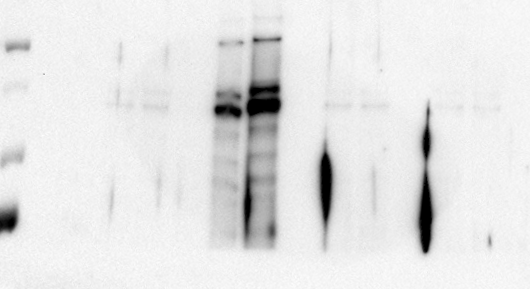

Supplement: Figure 3—source data 3. [file elife-82324-fig3-data3.zip › Figure 3-source data 3/Figure 3C Repeat1/Bead-bound-ATRIP.tif]

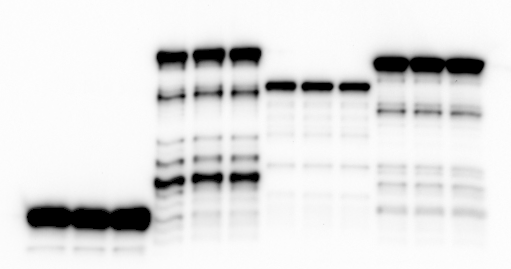

Supplement: Figure 3—source data 3. [file elife-82324-fig3-data3.zip › Figure 3-source data 3/Figure 3C Repeat1/Input-GST.tif]

Figure 3D

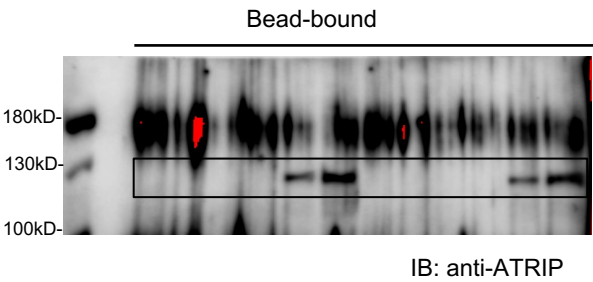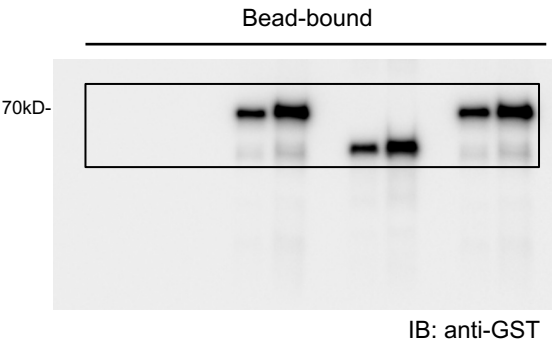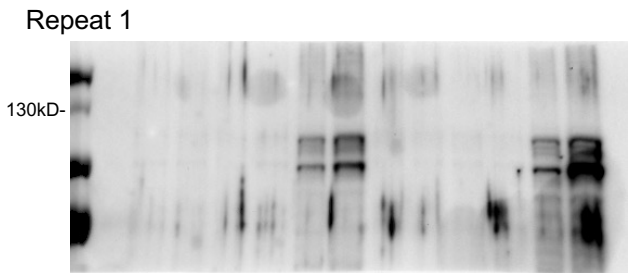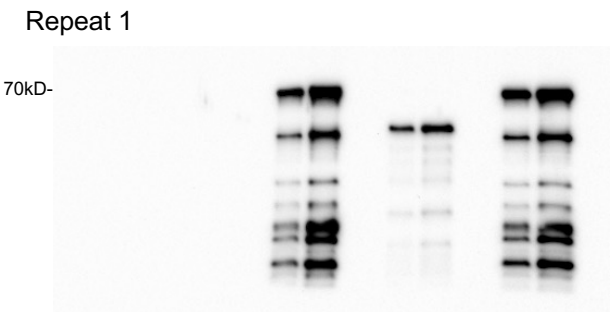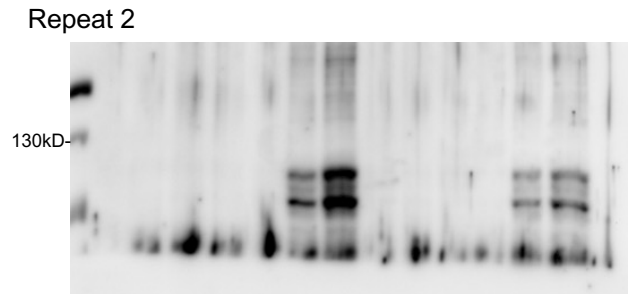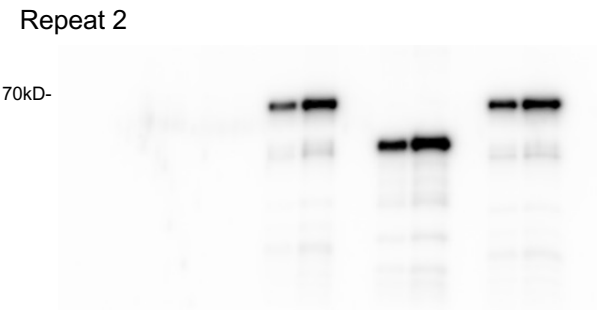

Figure 3D

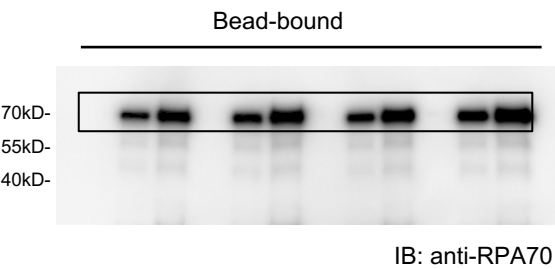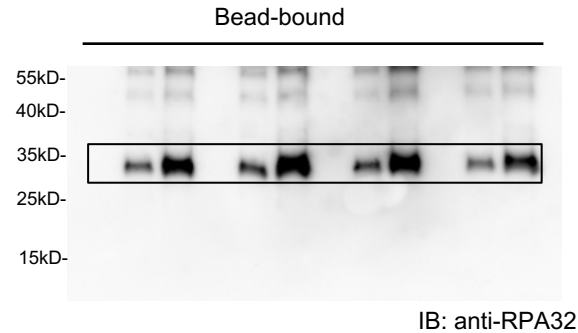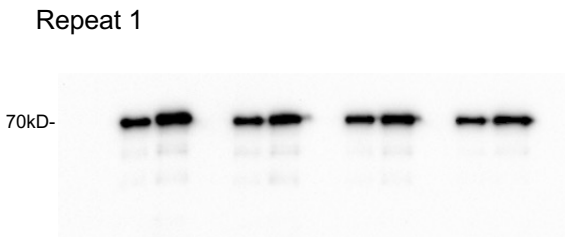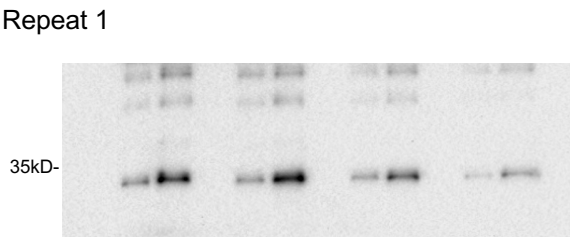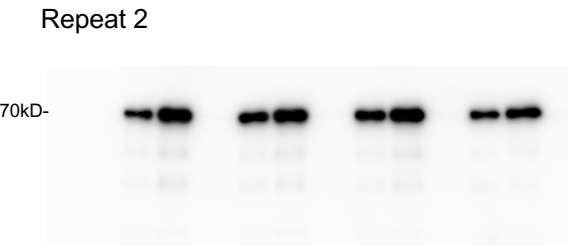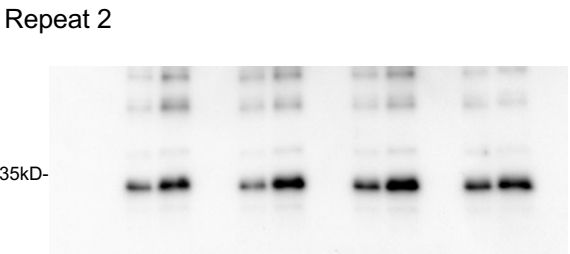

Figure 3D

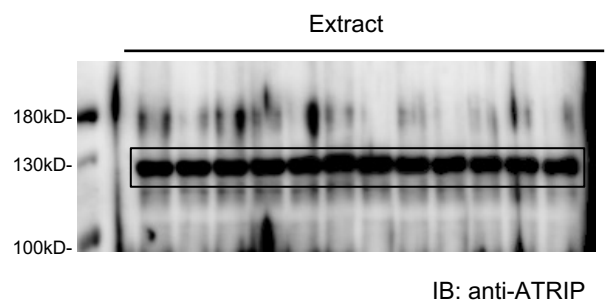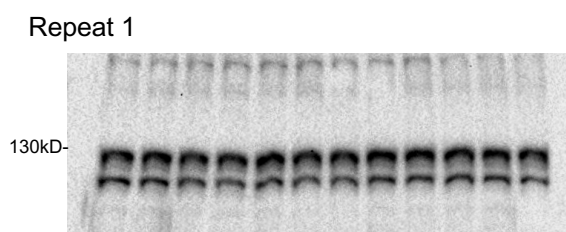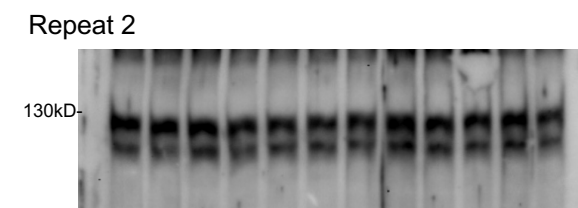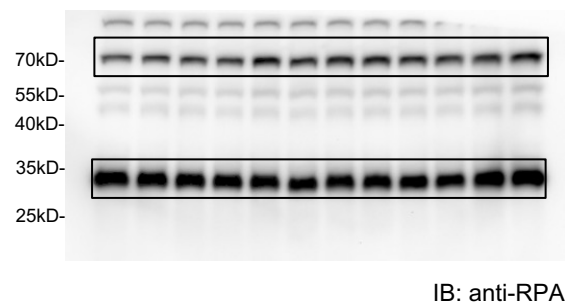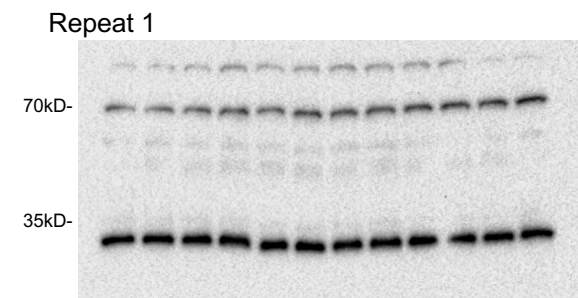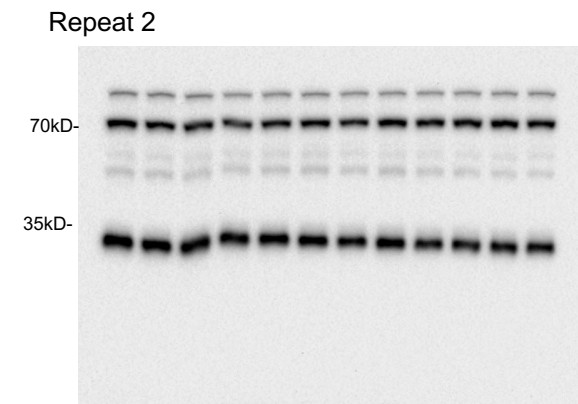

Figure 3D

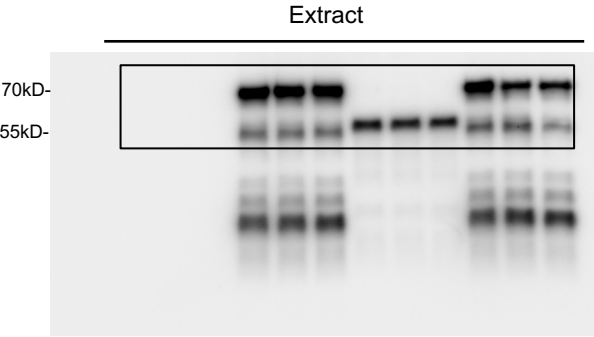

IB: anti-GST

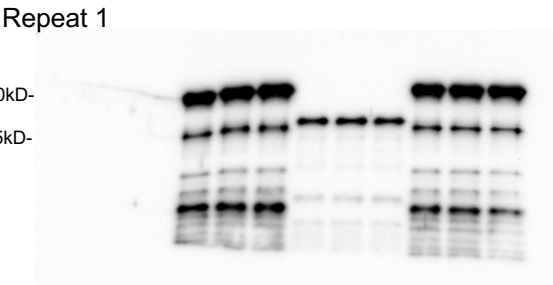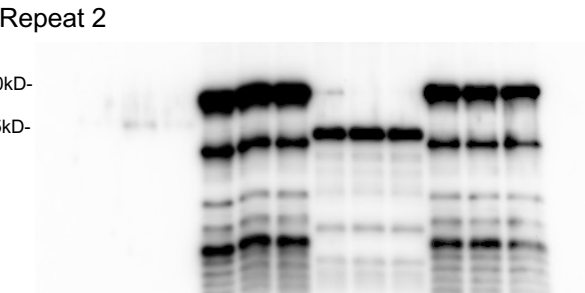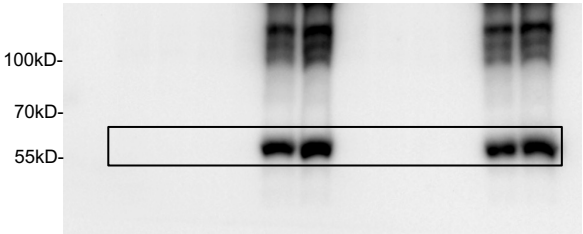

IB: Chk1-P

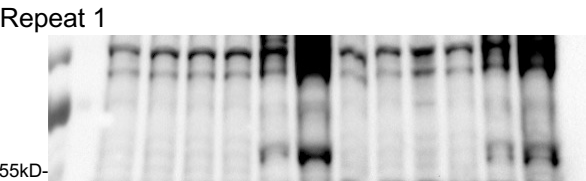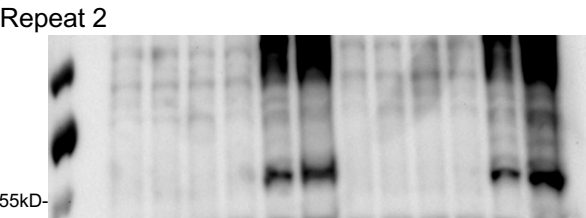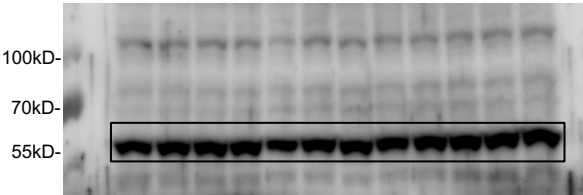

IB: Chk1

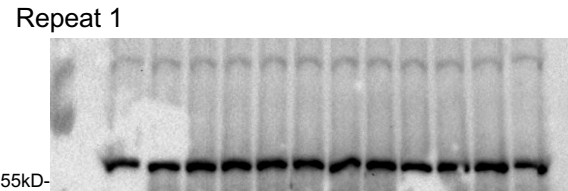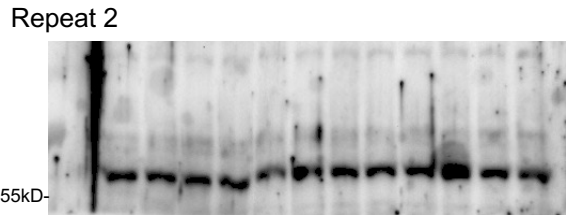

Supplement: Figure 3—source data 4. [file elife-82324-fig3-data4.zip › Figure 3-source data 4/IB-data-Figure 3D.pdf]

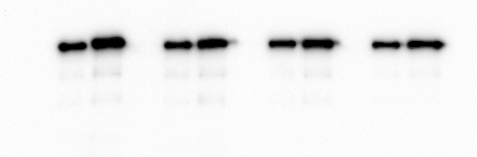

Supplement: Figure 3—source data 4. [file elife-82324-fig3-data4.zip › Figure 3-source data 4/Figure 3D Repeat1/Bead-bound-RPA70.tif]

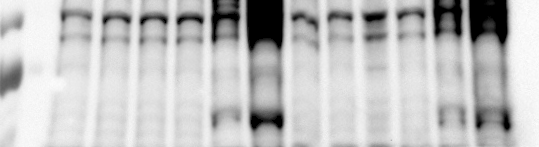

Supplement: Figure 3—source data 4. [file elife-82324-fig3-data4.zip › Figure 3-source data 4/Figure 3D Repeat1/Extract-CHK1-P.tif]

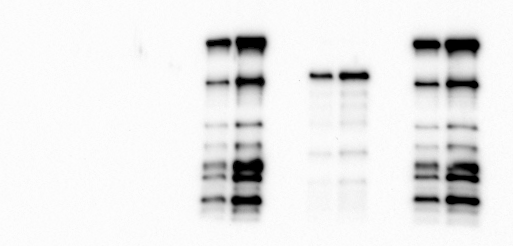

Supplement: Figure 3—source data 4. [file elife-82324-fig3-data4.zip › Figure 3-source data 4/Figure 3D Repeat1/Bead-bound-GST.tif]

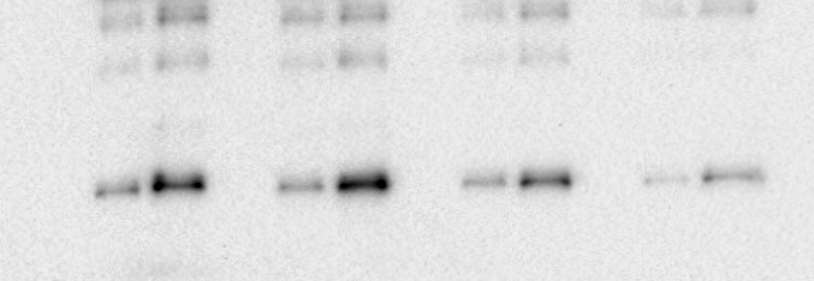

Supplement: Figure 3—source data 4. [file elife-82324-fig3-data4.zip › Figure 3-source data 4/Figure 3D Repeat1/Bead-bound-RPA32.tif]

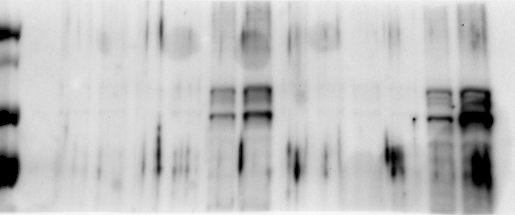

Supplement: Figure 3—source data 4. [file elife-82324-fig3-data4.zip › Figure 3-source data 4/Figure 3D Repeat1/Bead-bound-ATRIP.tif]

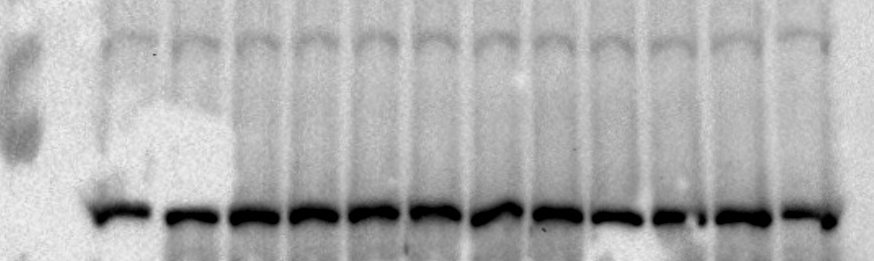

Supplement: Figure 3—source data 4. [file elife-82324-fig3-data4.zip › Figure 3-source data 4/Figure 3D Repeat1/Extract-CHK1.jpg]

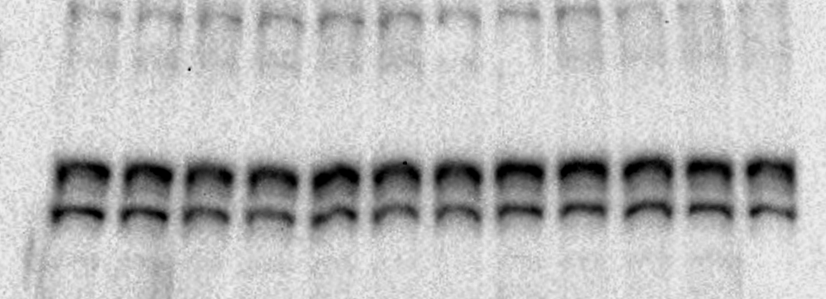

Supplement: Figure 3—source data 4. [file elife-82324-fig3-data4.zip › Figure 3-source data 4/Figure 3D Repeat1/Extract-ATRIP.tif]

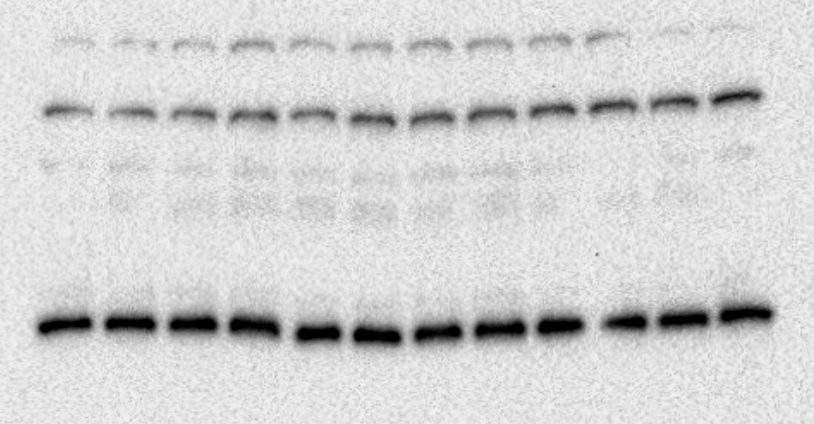

Supplement: Figure 3—source data 4. [file elife-82324-fig3-data4.zip › Figure 3-source data 4/Figure 3D Repeat1/Extract-RPA.tif]

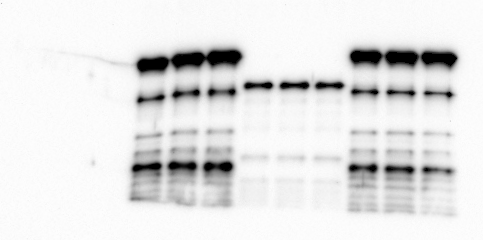

Supplement: Figure 3—source data 4. [file elife-82324-fig3-data4.zip › Figure 3-source data 4/Figure 3D Repeat1/Extract-GST.tif]

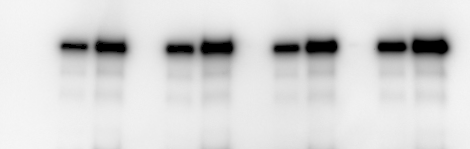

Supplement: Figure 3—source data 4. [file elife-82324-fig3-data4.zip › Figure 3-source data 4/Figure 3D initial trial/Bead-bound-RPA70.tif]

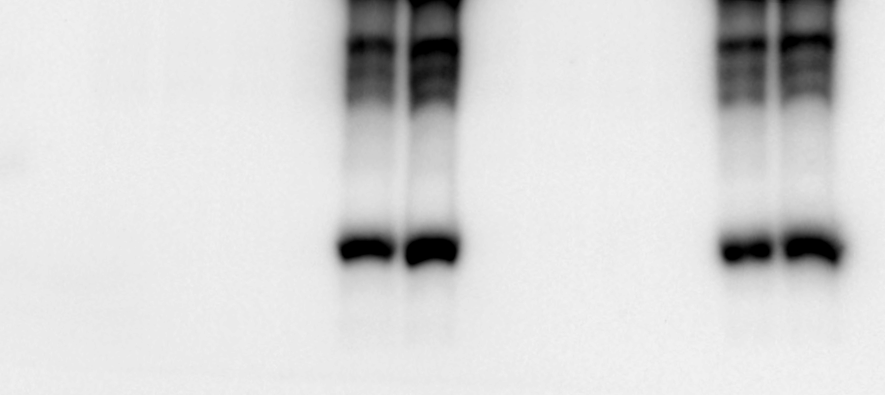

Supplement: Figure 3—source data 4. [file elife-82324-fig3-data4.zip › Figure 3-source data 4/Figure 3D initial trial/Extract-Chk1-P.tif]

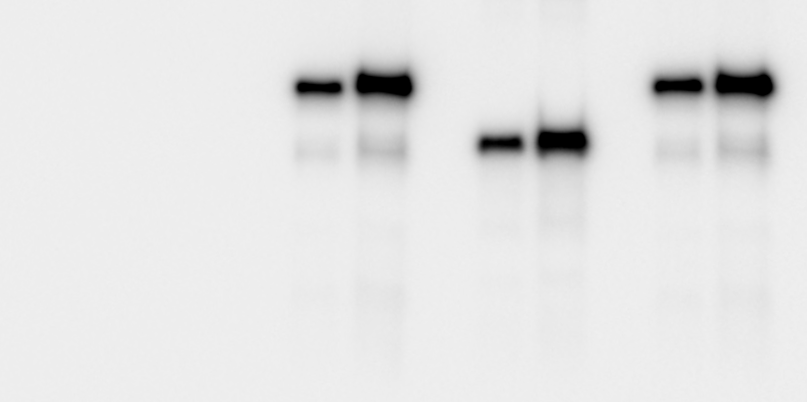

Supplement: Figure 3—source data 4. [file elife-82324-fig3-data4.zip › Figure 3-source data 4/Figure 3D initial trial/Bead-bound-GST.tif]

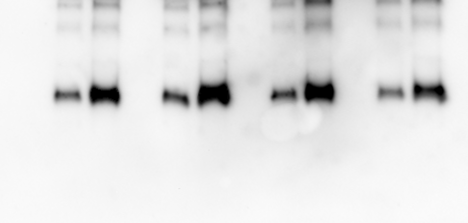

Supplement: Figure 3—source data 4. [file elife-82324-fig3-data4.zip › Figure 3-source data 4/Figure 3D initial trial/Bead-bound-RPA32.tif]

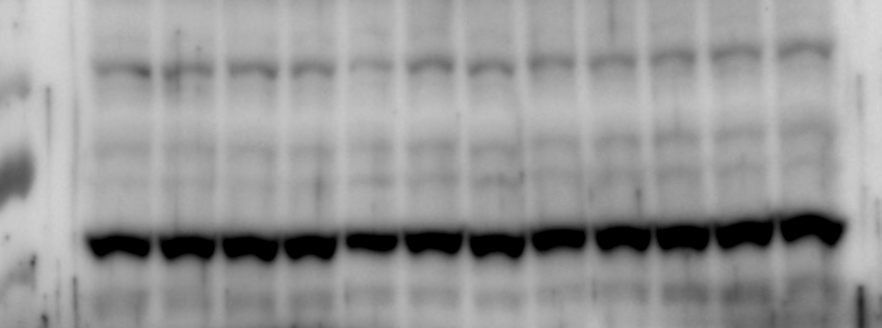

Supplement: Figure 3—source data 4. [file elife-82324-fig3-data4.zip › Figure 3-source data 4/Figure 3D initial trial/Extract-Chk1.tif]

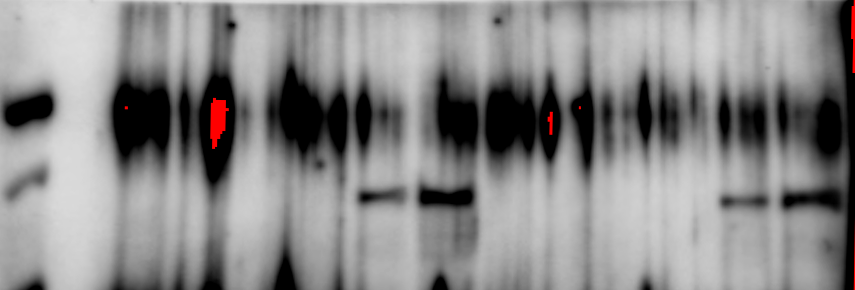

Supplement: Figure 3—source data 4. [file elife-82324-fig3-data4.zip › Figure 3-source data 4/Figure 3D initial trial/Bead-bound-ATRIP.tif]

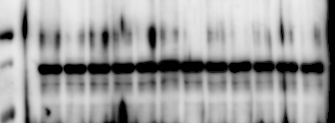

Supplement: Figure 3—source data 4. [file elife-82324-fig3-data4.zip › Figure 3-source data 4/Figure 3D initial trial/Extract-ATRIP.tif]

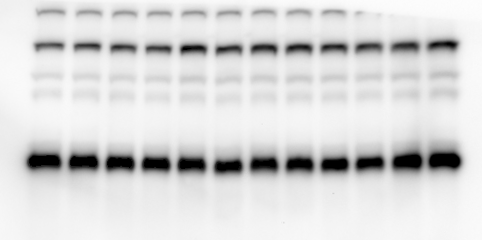

Supplement: Figure 3—source data 4. [file elife-82324-fig3-data4.zip › Figure 3-source data 4/Figure 3D initial trial/Extract-RPA.tif]

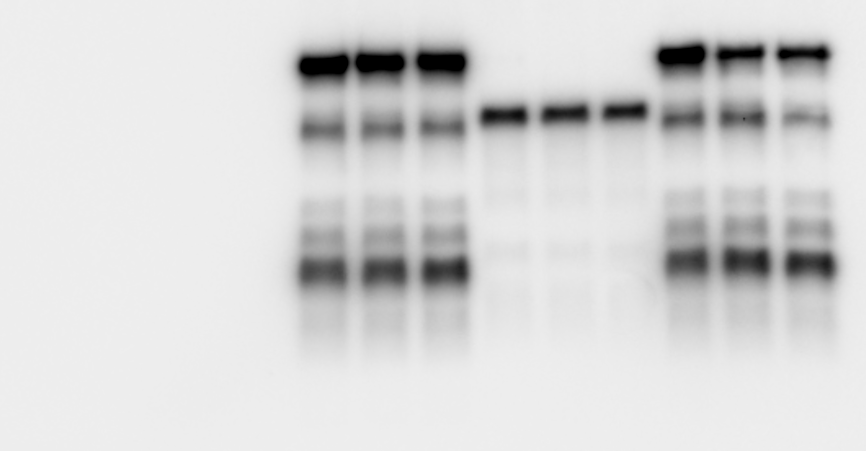

Supplement: Figure 3—source data 4. [file elife-82324-fig3-data4.zip › Figure 3-source data 4/Figure 3D initial trial/Extract-GST.tif]

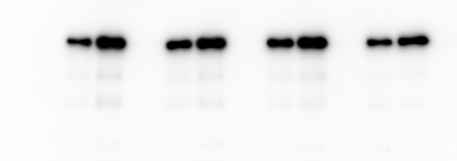

Supplement: Figure 3—source data 4. [file elife-82324-fig3-data4.zip › Figure 3-source data 4/Figure 3D Repeat2/Bead-bound--RPA70.tif]

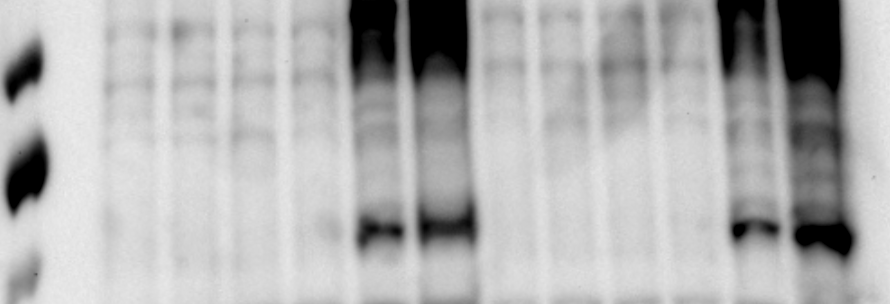

Supplement: Figure 3—source data 4. [file elife-82324-fig3-data4.zip › Figure 3-source data 4/Figure 3D Repeat2/Extract-Chk1-P.tif]

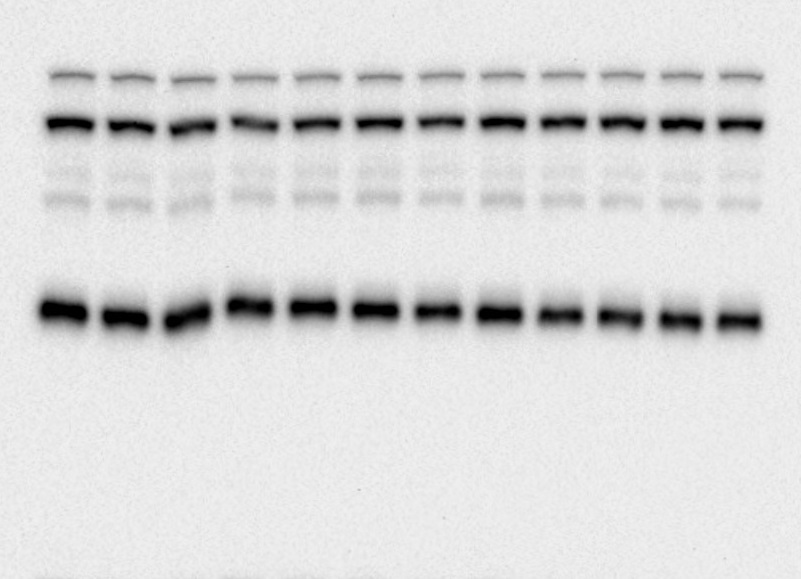

Supplement: Figure 3—source data 4. [file elife-82324-fig3-data4.zip › Figure 3-source data 4/Figure 3D Repeat2/Extract-RPA.jpg]

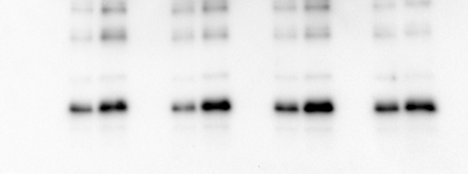

Supplement: Figure 3—source data 4. [file elife-82324-fig3-data4.zip › Figure 3-source data 4/Figure 3D Repeat2/Bead-bound--RPA32.tif]

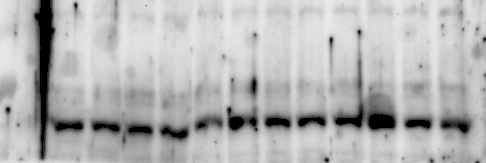

Supplement: Figure 3—source data 4. [file elife-82324-fig3-data4.zip › Figure 3-source data 4/Figure 3D Repeat2/Extract-Chk1.tif]

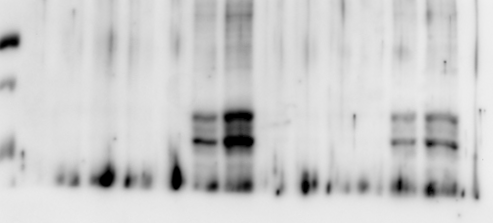

Supplement: Figure 3—source data 4. [file elife-82324-fig3-data4.zip › Figure 3-source data 4/Figure 3D Repeat2/Bead-bound-ATRIP.tif]

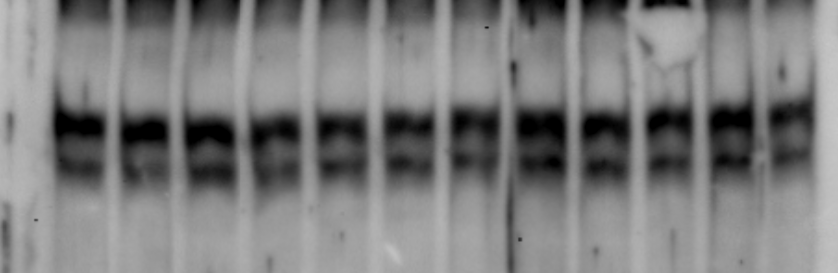

Supplement: Figure 3—source data 4. [file elife-82324-fig3-data4.zip › Figure 3-source data 4/Figure 3D Repeat2/Extract-ATRIP.tif]

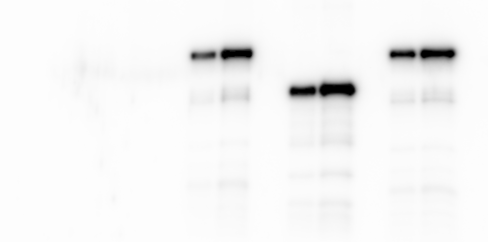

Supplement: Figure 3—source data 4. [file elife-82324-fig3-data4.zip › Figure 3-source data 4/Figure 3D Repeat2/Bead-bound--GST.tif]

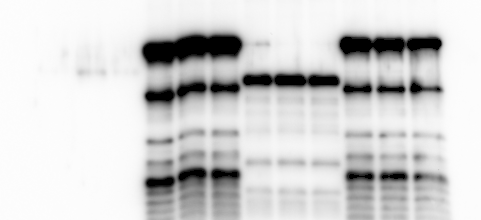

Supplement: Figure 3—source data 4. [file elife-82324-fig3-data4.zip › Figure 3-source data 4/Figure 3D Repeat2/Extract-GST.tif]

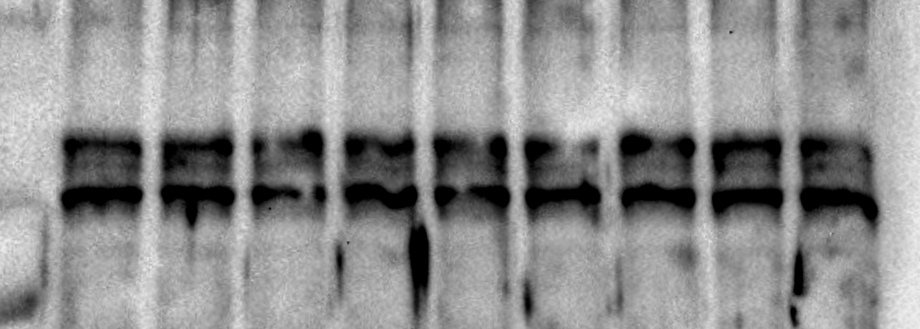

Supplement: Figure 3—figure supplement 1—source data 1. [file elife-82324-fig3-figsupp1-data1.zip › Figure 3-figure supplement 1-souce data 1/Figure 3S1 initial trial/Extract--ATRIP.tif]

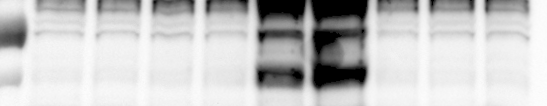

Supplement: Figure 3—figure supplement 1—source data 1. [file elife-82324-fig3-figsupp1-data1.zip › Figure 3-figure supplement 1-souce data 1/Figure 3S1 initial trial/Extract-Chk1-P.tif]

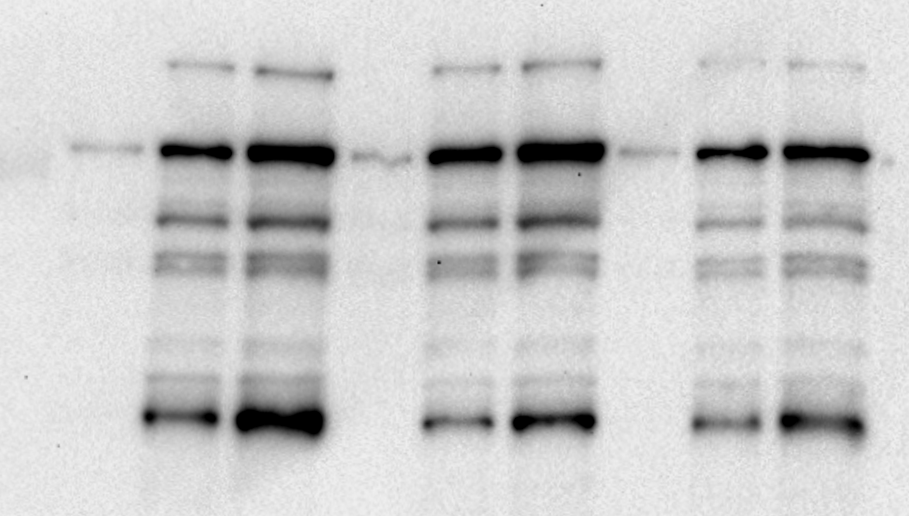

Supplement: Figure 3—figure supplement 1—source data 1. [file elife-82324-fig3-figsupp1-data1.zip › Figure 3-figure supplement 1-souce data 1/Figure 3S1 initial trial/Bead-RPA.tif]

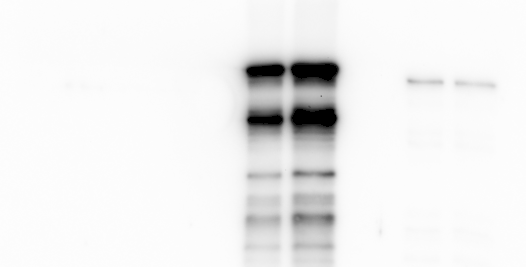

Supplement: Figure 3—figure supplement 1—source data 1. [file elife-82324-fig3-figsupp1-data1.zip › Figure 3-figure supplement 1-souce data 1/Figure 3S1 initial trial/Bead-GST.tif]

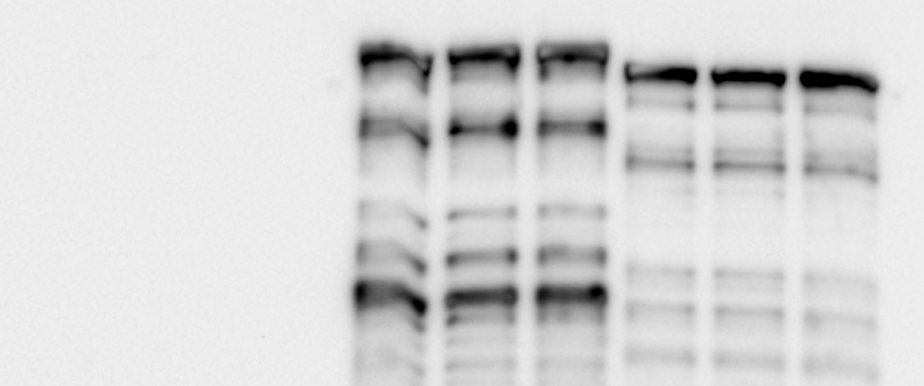

Supplement: Figure 3—figure supplement 1—source data 1. [file elife-82324-fig3-figsupp1-data1.zip › Figure 3-figure supplement 1-souce data 1/Figure 3S1 initial trial/Extract--GST.tif]

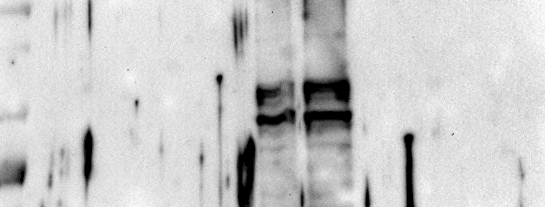

Supplement: Figure 3—figure supplement 1—source data 1. [file elife-82324-fig3-figsupp1-data1.zip › Figure 3-figure supplement 1-souce data 1/Figure 3S1 initial trial/Bead-ATRIP.tif]

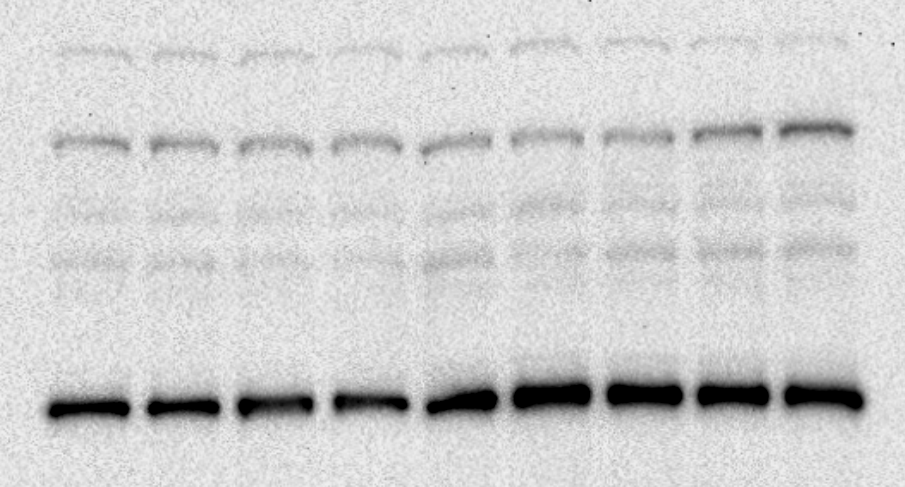

Supplement: Figure 3—figure supplement 1—source data 1. [file elife-82324-fig3-figsupp1-data1.zip › Figure 3-figure supplement 1-souce data 1/Figure 3S1 initial trial/Extract--RPA.tif]

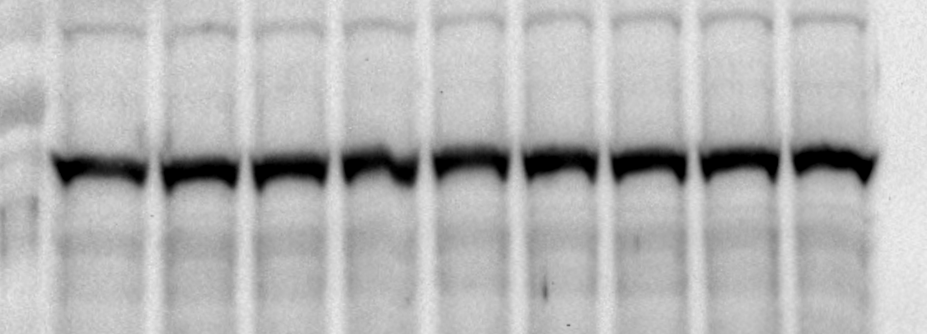

Supplement: Figure 3—figure supplement 1—source data 1. [file elife-82324-fig3-figsupp1-data1.zip › Figure 3-figure supplement 1-souce data 1/Figure 3S1 initial trial/Extract-Chk1.tif]

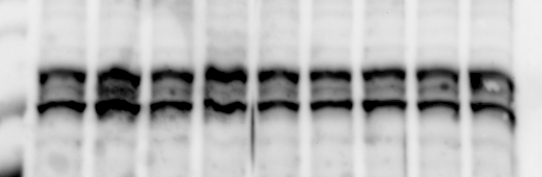

Supplement: Figure 3—figure supplement 1—source data 1. [file elife-82324-fig3-figsupp1-data1.zip › Figure 3-figure supplement 1-souce data 1/Figure 3S1 Repeat1/Extract--ATRIP.tif]

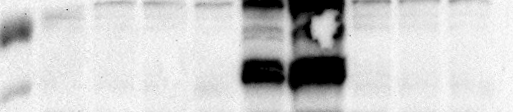

Supplement: Figure 3—figure supplement 1—source data 1. [file elife-82324-fig3-figsupp1-data1.zip › Figure 3-figure supplement 1-souce data 1/Figure 3S1 Repeat1/Extract-Chk1-P.tif]

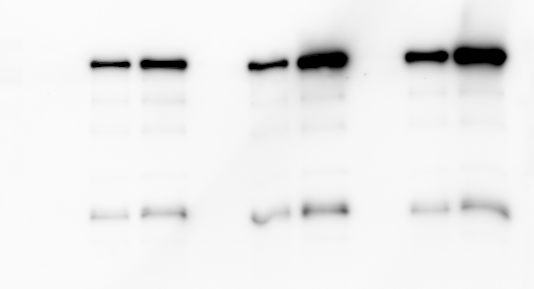

Supplement: Figure 3—figure supplement 1—source data 1. [file elife-82324-fig3-figsupp1-data1.zip › Figure 3-figure supplement 1-souce data 1/Figure 3S1 Repeat1/Bead-RPA.tif]

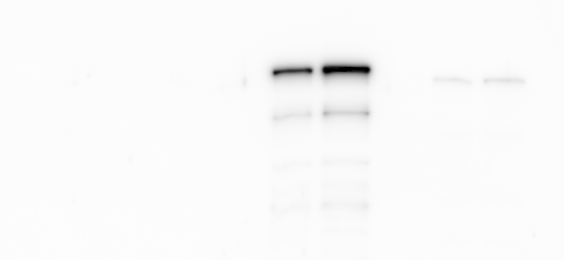

Supplement: Figure 3—figure supplement 1—source data 1. [file elife-82324-fig3-figsupp1-data1.zip › Figure 3-figure supplement 1-souce data 1/Figure 3S1 Repeat1/Bead-GST.tif]

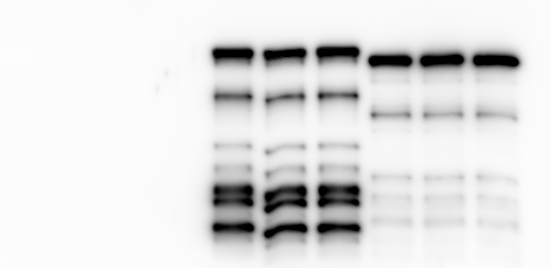

Supplement: Figure 3—figure supplement 1—source data 1. [file elife-82324-fig3-figsupp1-data1.zip › Figure 3-figure supplement 1-souce data 1/Figure 3S1 Repeat1/Extract--GST.tif]

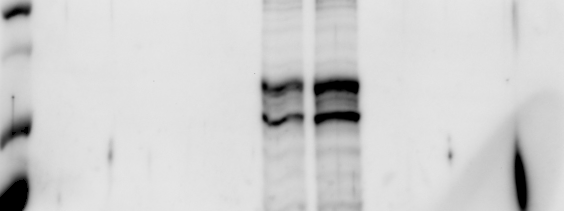

Supplement: Figure 3—figure supplement 1—source data 1. [file elife-82324-fig3-figsupp1-data1.zip › Figure 3-figure supplement 1-souce data 1/Figure 3S1 Repeat1/Bead-ATRIP.tif]

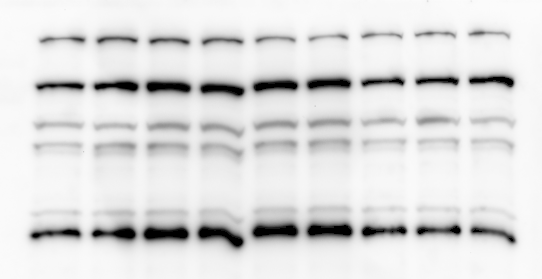

Supplement: Figure 3—figure supplement 1—source data 1. [file elife-82324-fig3-figsupp1-data1.zip › Figure 3-figure supplement 1-souce data 1/Figure 3S1 Repeat1/Extract--RPA.tif]

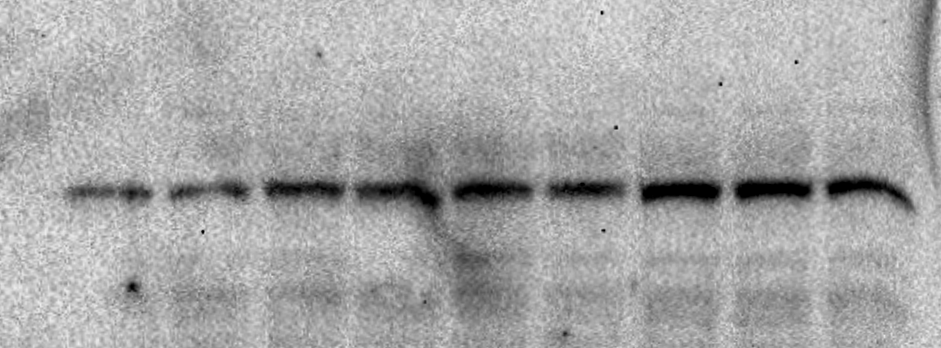

Supplement: Figure 3—figure supplement 1—source data 1. [file elife-82324-fig3-figsupp1-data1.zip › Figure 3-figure supplement 1-souce data 1/Figure 3S1 Repeat1/Extract-Chk1.tif]

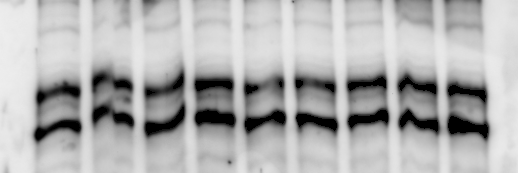

Supplement: Figure 3—figure supplement 1—source data 1. [file elife-82324-fig3-figsupp1-data1.zip › Figure 3-figure supplement 1-souce data 1/Figure 3S1 Repeat2/Extract--ATRIP.tif]

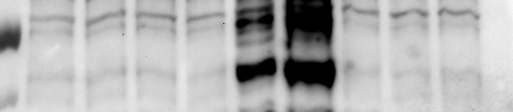

Supplement: Figure 3—figure supplement 1—source data 1. [file elife-82324-fig3-figsupp1-data1.zip › Figure 3-figure supplement 1-souce data 1/Figure 3S1 Repeat2/Extract-Chk1-P.tif]

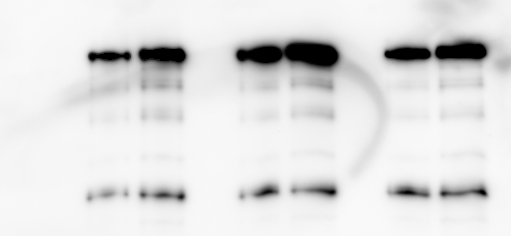

Supplement: Figure 3—figure supplement 1—source data 1. [file elife-82324-fig3-figsupp1-data1.zip › Figure 3-figure supplement 1-souce data 1/Figure 3S1 Repeat2/Bead-RPA.tif]

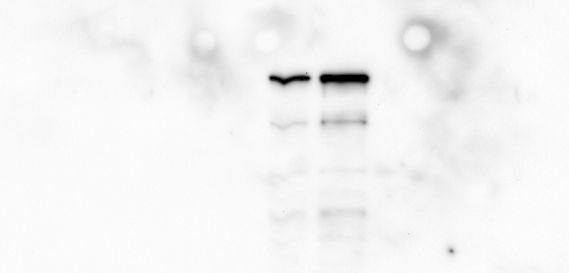

Supplement: Figure 3—figure supplement 1—source data 1. [file elife-82324-fig3-figsupp1-data1.zip › Figure 3-figure supplement 1-souce data 1/Figure 3S1 Repeat2/Bead-GST.tif]

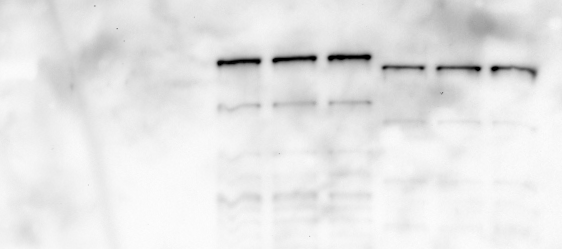

Supplement: Figure 3—figure supplement 1—source data 1. [file elife-82324-fig3-figsupp1-data1.zip › Figure 3-figure supplement 1-souce data 1/Figure 3S1 Repeat2/Extract--GST.tif]

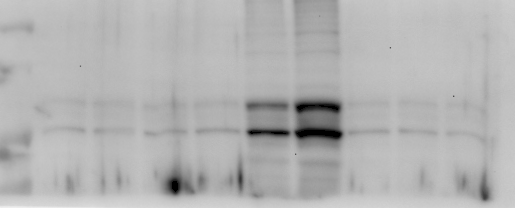

Supplement: Figure 3—figure supplement 1—source data 1. [file elife-82324-fig3-figsupp1-data1.zip › Figure 3-figure supplement 1-souce data 1/Figure 3S1 Repeat2/Bead-ATRIP.tif]

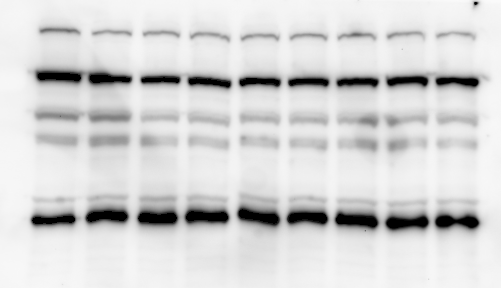

Supplement: Figure 3—figure supplement 1—source data 1. [file elife-82324-fig3-figsupp1-data1.zip › Figure 3-figure supplement 1-souce data 1/Figure 3S1 Repeat2/Extract--RPA.tif]

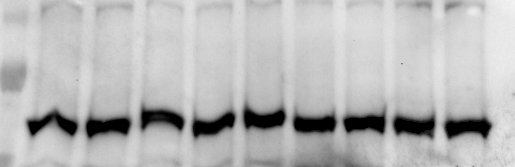

Supplement: Figure 3—figure supplement 1—source data 1. [file elife-82324-fig3-figsupp1-data1.zip › Figure 3-figure supplement 1-souce data 1/Figure 3S1 Repeat2/Extract-Chk1.tif]

Figure 4A

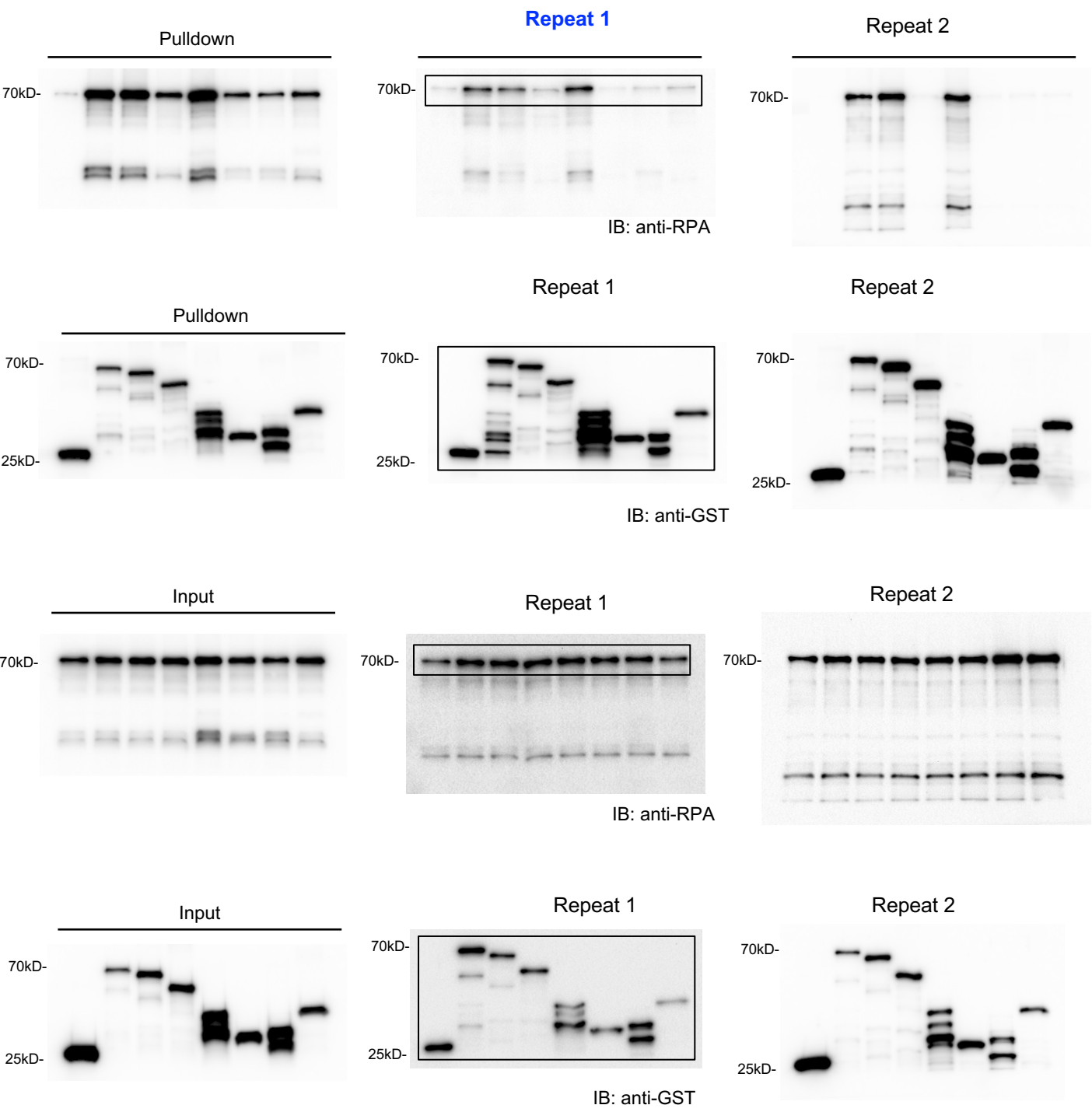

Supplement: Figure 4—source data 1. [file elife-82324-fig4-data1.zip › Figure 4-source data 1/IB-data-Figure 4A.pdf]

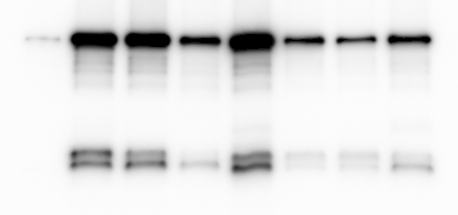

Supplement: Figure 4—source data 1. [file elife-82324-fig4-data1.zip › Figure 4-source data 1/Figure 4A initial trial/Pulldown-RPA.tif]

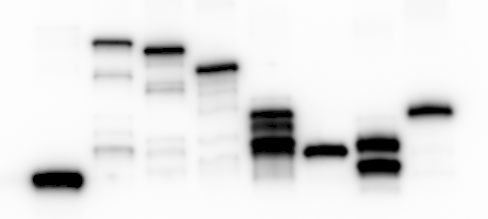

Supplement: Figure 4—source data 1. [file elife-82324-fig4-data1.zip › Figure 4-source data 1/Figure 4A initial trial/Pulldown-GST.tif]

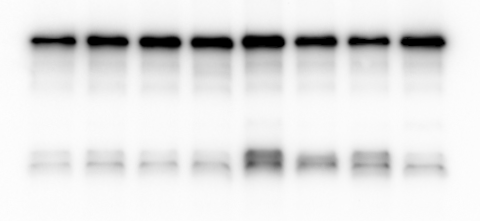

Supplement: Figure 4—source data 1. [file elife-82324-fig4-data1.zip › Figure 4-source data 1/Figure 4A initial trial/Input-RPA.tif]

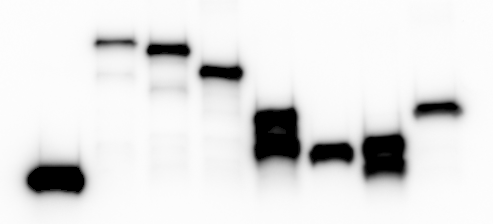

Supplement: Figure 4—source data 1. [file elife-82324-fig4-data1.zip › Figure 4-source data 1/Figure 4A initial trial/Input-GST.tif]

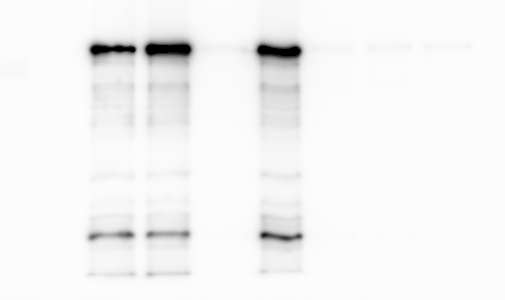

Supplement: Figure 4—source data 1. [file elife-82324-fig4-data1.zip › Figure 4-source data 1/Figure 4A Repeat2/Pulldown-RPA.tif]

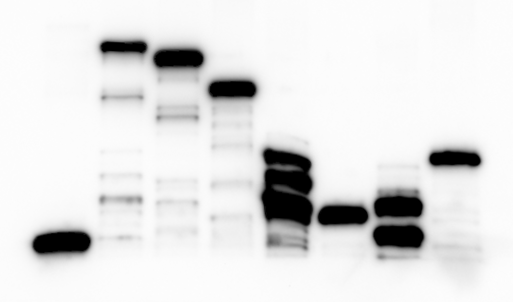

Supplement: Figure 4—source data 1. [file elife-82324-fig4-data1.zip › Figure 4-source data 1/Figure 4A Repeat2/Pulldown-GST.tif]

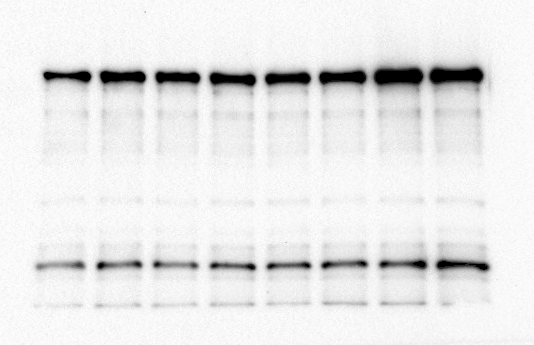

Supplement: Figure 4—source data 1. [file elife-82324-fig4-data1.zip › Figure 4-source data 1/Figure 4A Repeat2/Input-RPA.tif]

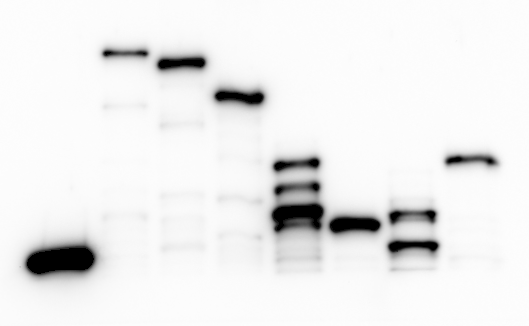

Supplement: Figure 4—source data 1. [file elife-82324-fig4-data1.zip › Figure 4-source data 1/Figure 4A Repeat2/Input-GST.tif]

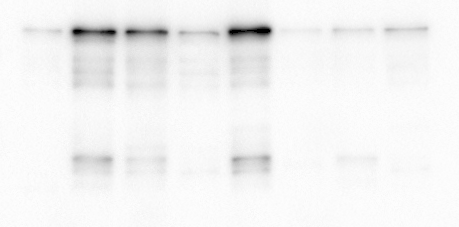

Supplement: Figure 4—source data 1. [file elife-82324-fig4-data1.zip › Figure 4-source data 1/Figure 4A Repeat1/Pulldown-RPA.tif]

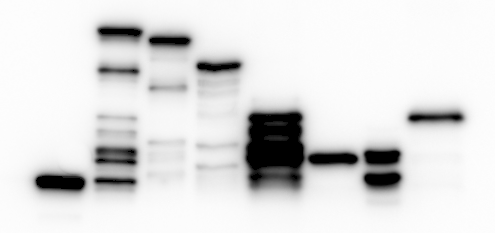

Supplement: Figure 4—source data 1. [file elife-82324-fig4-data1.zip › Figure 4-source data 1/Figure 4A Repeat1/Pulldown-GST.tif]

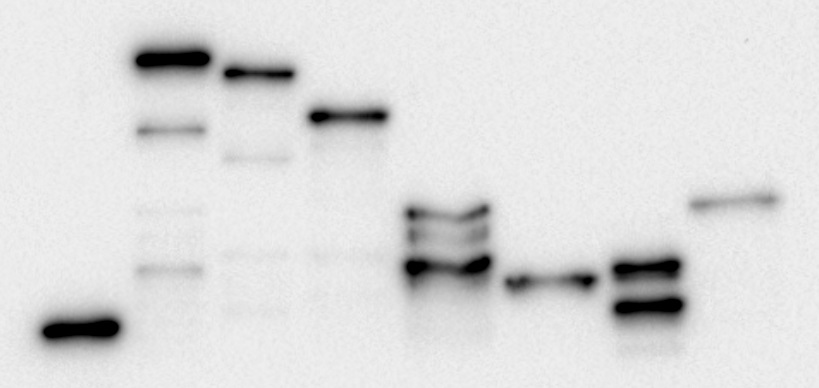

Supplement: Figure 4—source data 1. [file elife-82324-fig4-data1.zip › Figure 4-source data 1/Figure 4A Repeat1/Input-GST.jpg]

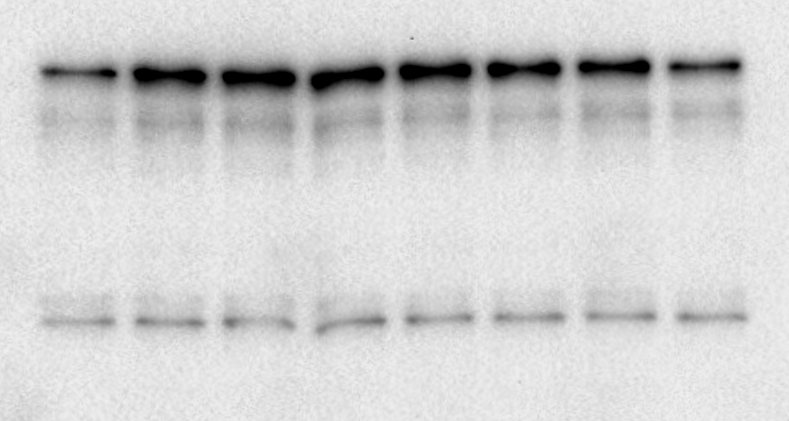

Supplement: Figure 4—source data 1. [file elife-82324-fig4-data1.zip › Figure 4-source data 1/Figure 4A Repeat1/Input-RPA.tif]

Figure 4C

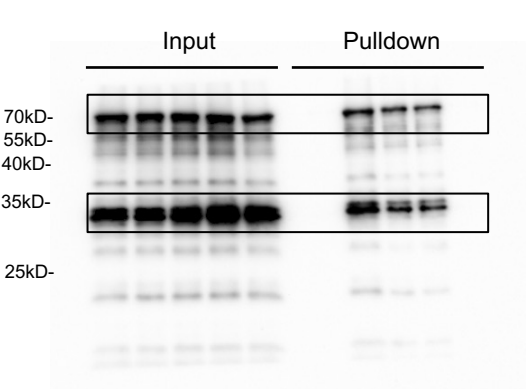

IB: anti-RPA

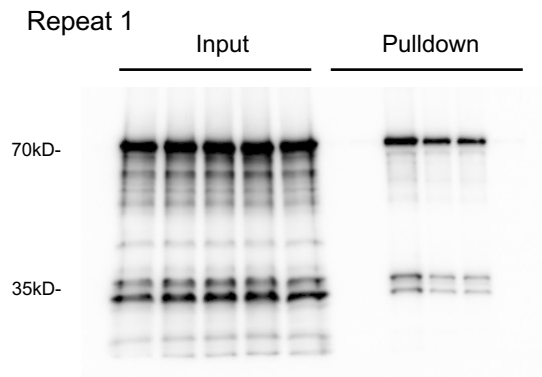

Repeat 2

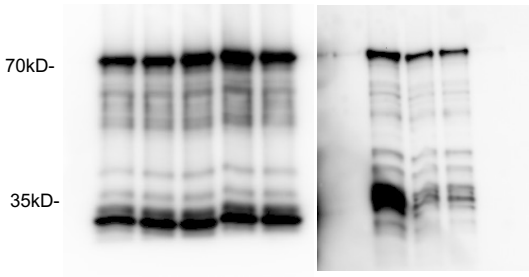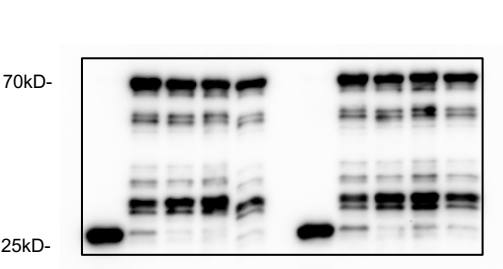

IB: anti-GST

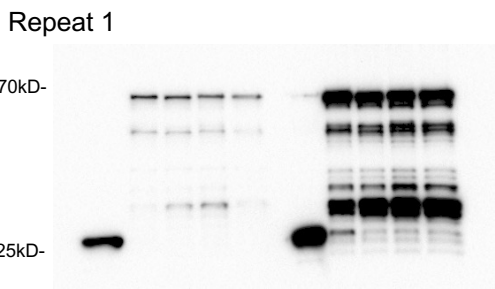

Repeat 2

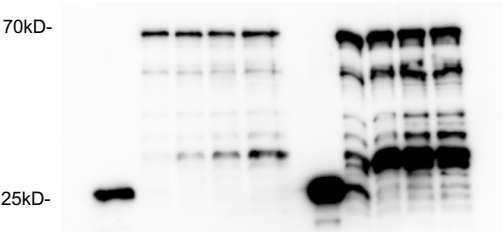

Supplement: Figure 4—source data 2. [file elife-82324-fig4-data2.zip › Figure 4-source data 2/IB-data-Figure 4C.pdf]

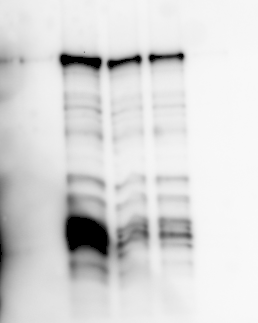

Supplement: Figure 4—source data 2. [file elife-82324-fig4-data2.zip › Figure 4-source data 2/Figure 4C Repeat2/Pulldown-RPA.tif]

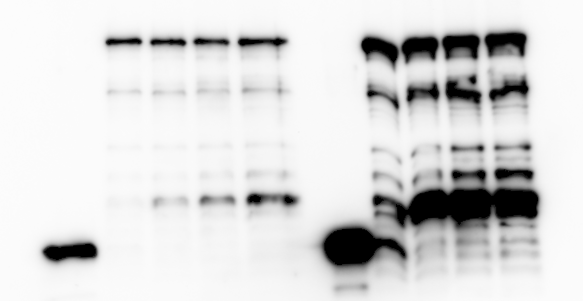

Supplement: Figure 4—source data 2. [file elife-82324-fig4-data2.zip › Figure 4-source data 2/Figure 4C Repeat2/Input&Pulldown-GST.tif]

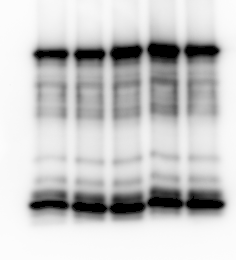

Supplement: Figure 4—source data 2. [file elife-82324-fig4-data2.zip › Figure 4-source data 2/Figure 4C Repeat2/Input-RPA.tif]

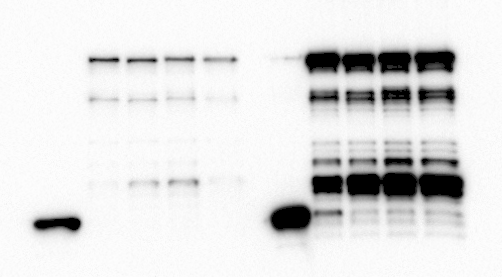

Supplement: Figure 4—source data 2. [file elife-82324-fig4-data2.zip › Figure 4-source data 2/Figure 4C Repeat1/Input&Pulldown-GST.tif]

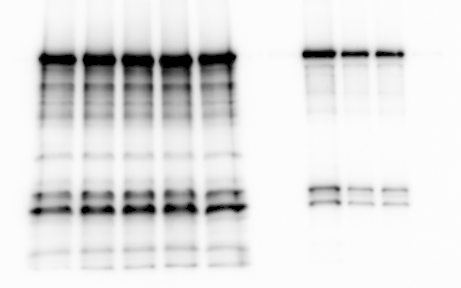

Supplement: Figure 4—source data 2. [file elife-82324-fig4-data2.zip › Figure 4-source data 2/Figure 4C Repeat1/Input&Pulldown-RPA.tif]

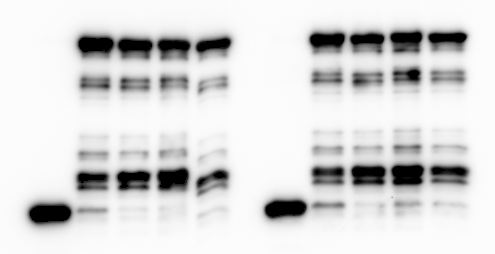

Supplement: Figure 4—source data 2. [file elife-82324-fig4-data2.zip › Figure 4-source data 2/Figure 4C initial trial/Input&Pulldown-GST.tif]

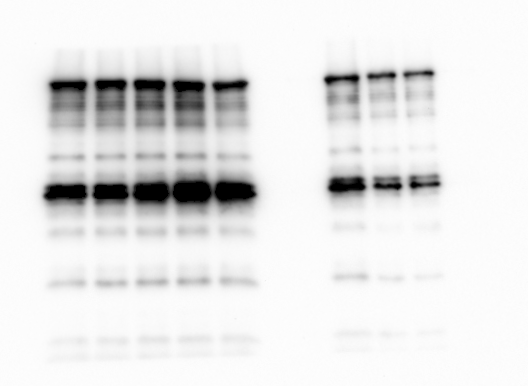

Supplement: Figure 4—source data 2. [file elife-82324-fig4-data2.zip › Figure 4-source data 2/Figure 4C initial trial/Input&Pulldown-RPA.tif]

Figure 4D

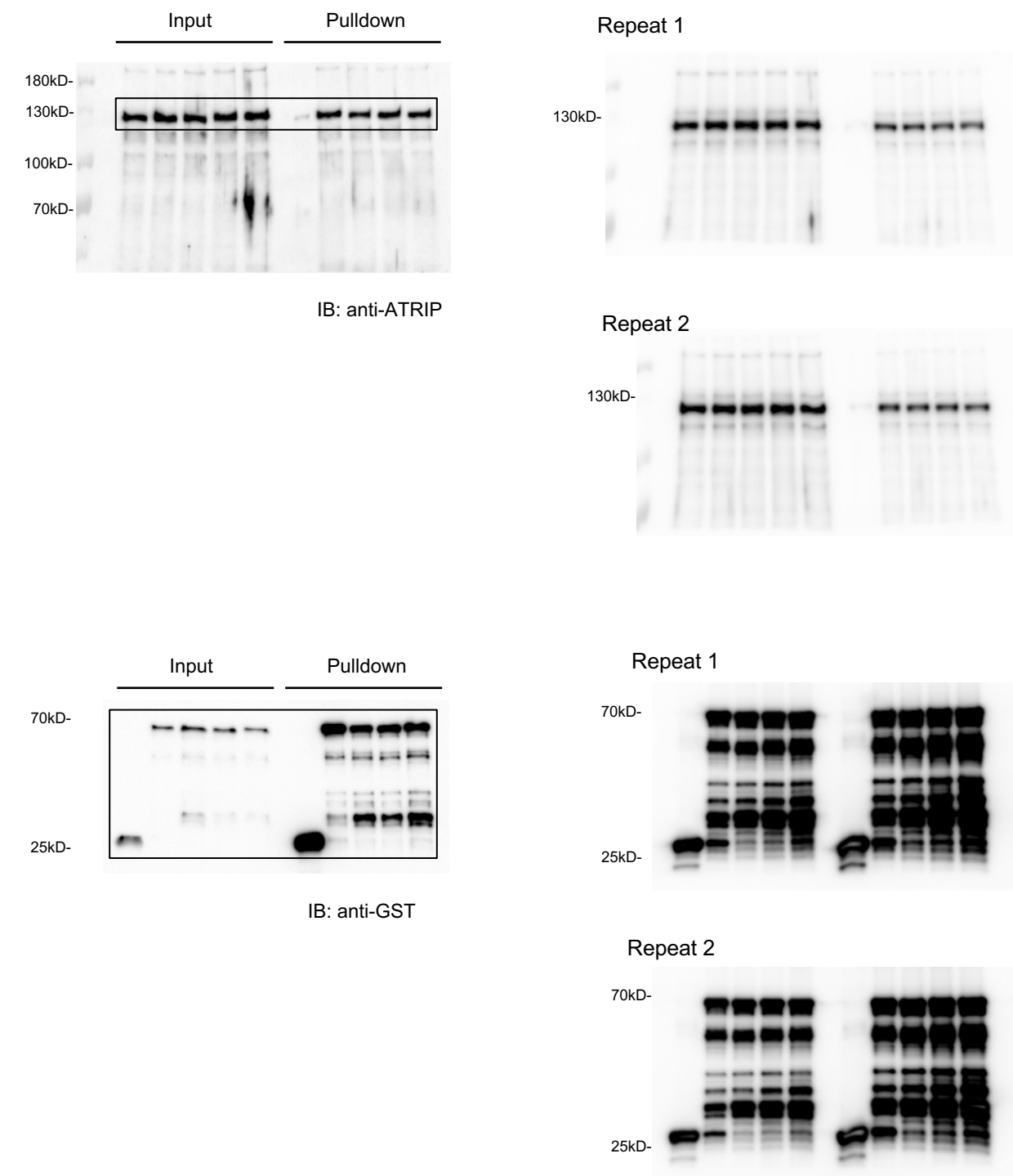

Supplement: Figure 4—source data 3. [file elife-82324-fig4-data3.zip › Figure 4-source data 3/IB-data-Figure 4D.pdf]

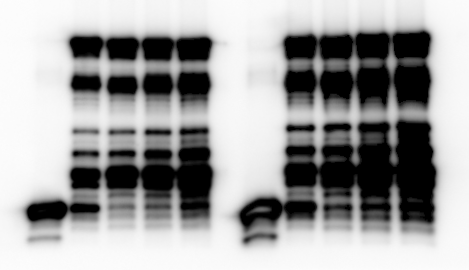

Supplement: Figure 4—source data 3. [file elife-82324-fig4-data3.zip › Figure 4-source data 3/Figure 4D Repeat1/Input&Pulldown-GST.tif]

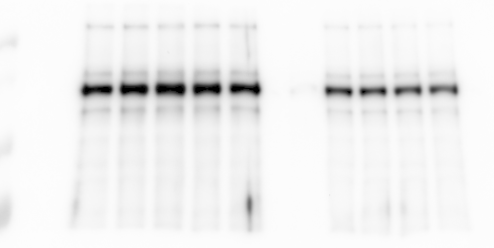

Supplement: Figure 4—source data 3. [file elife-82324-fig4-data3.zip › Figure 4-source data 3/Figure 4D Repeat1/Input&Pulldown-ATRIP.tif]

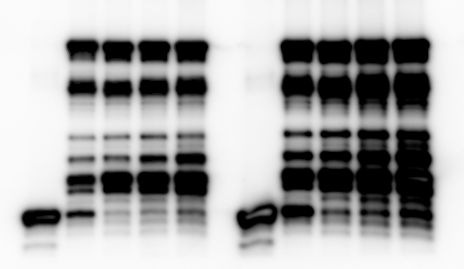

Supplement: Figure 4—source data 3. [file elife-82324-fig4-data3.zip › Figure 4-source data 3/Figure 4D Repeat2/Input and Pulldown-GST.tif]

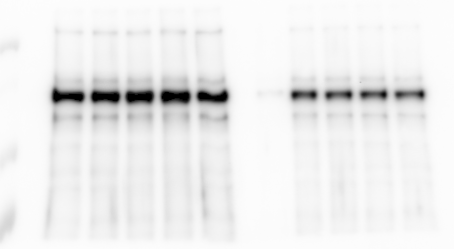

Supplement: Figure 4—source data 3. [file elife-82324-fig4-data3.zip › Figure 4-source data 3/Figure 4D Repeat2/Input and Pulldown-ATRIP.tif]

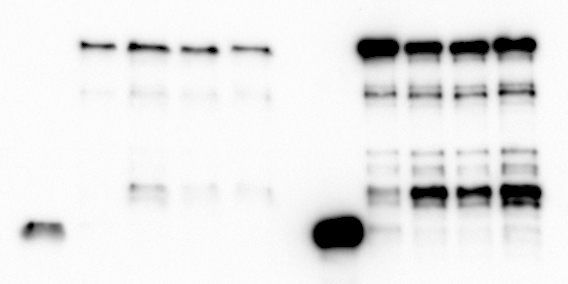

Supplement: Figure 4—source data 3. [file elife-82324-fig4-data3.zip › Figure 4-source data 3/Figure 4D initial trial/Input&Pulldown-GST.tif]

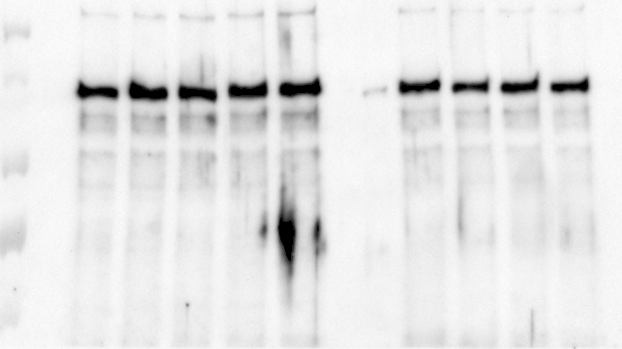

Supplement: Figure 4—source data 3. [file elife-82324-fig4-data3.zip › Figure 4-source data 3/Figure 4D initial trial/Input&Pulldown-ATRIP.tif]

Figure 4E

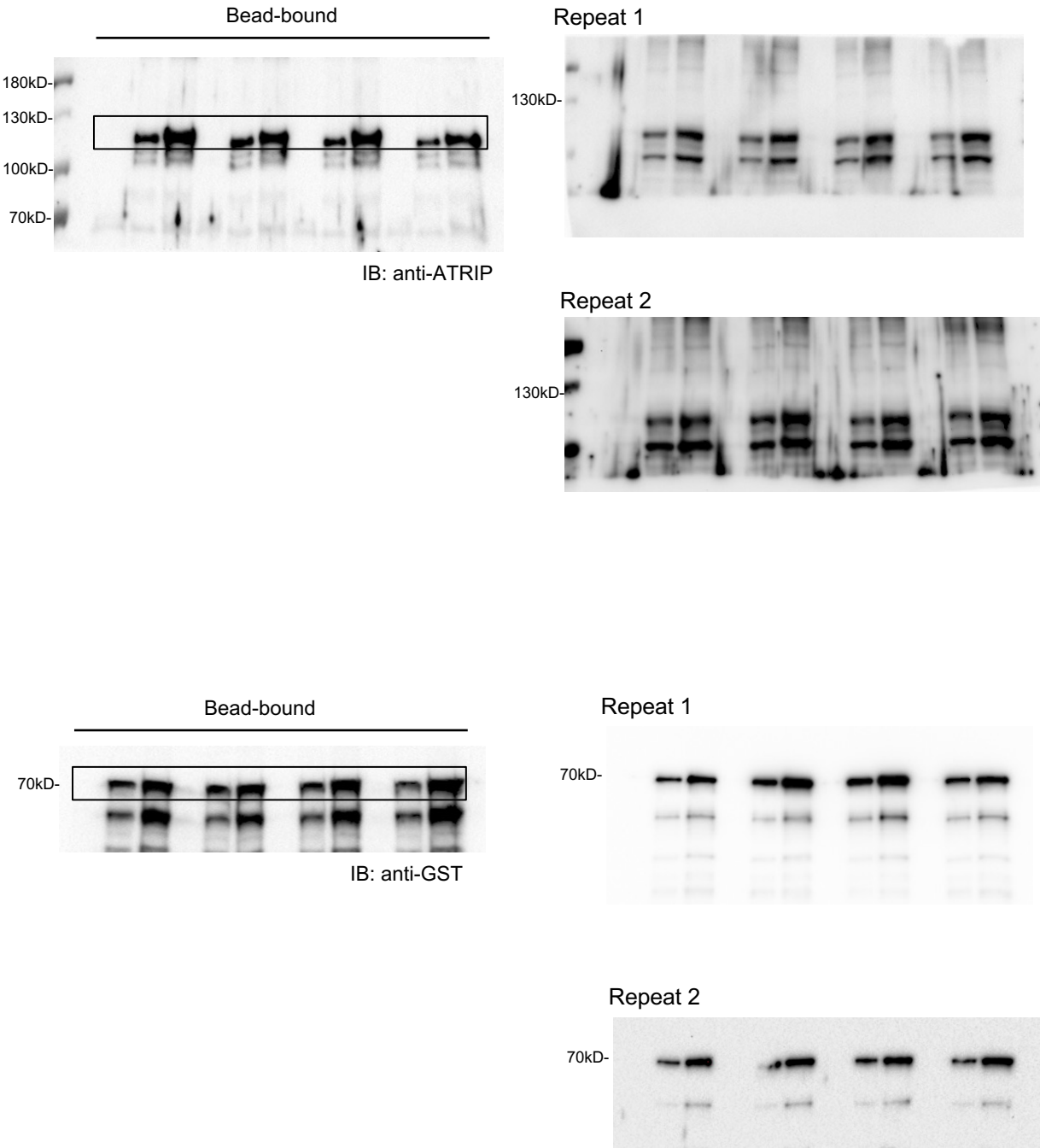

Figure 4E

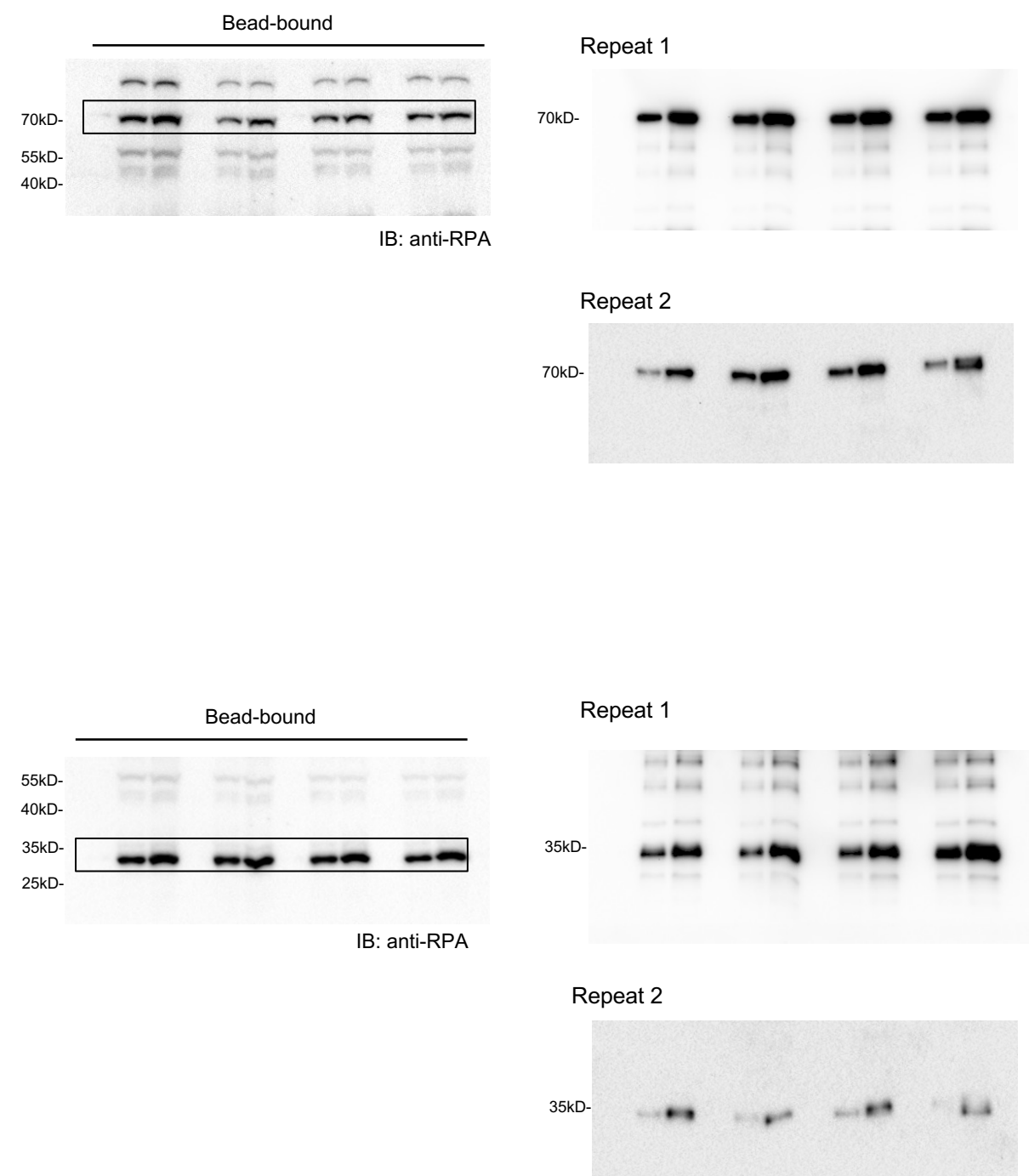

Figure 4E

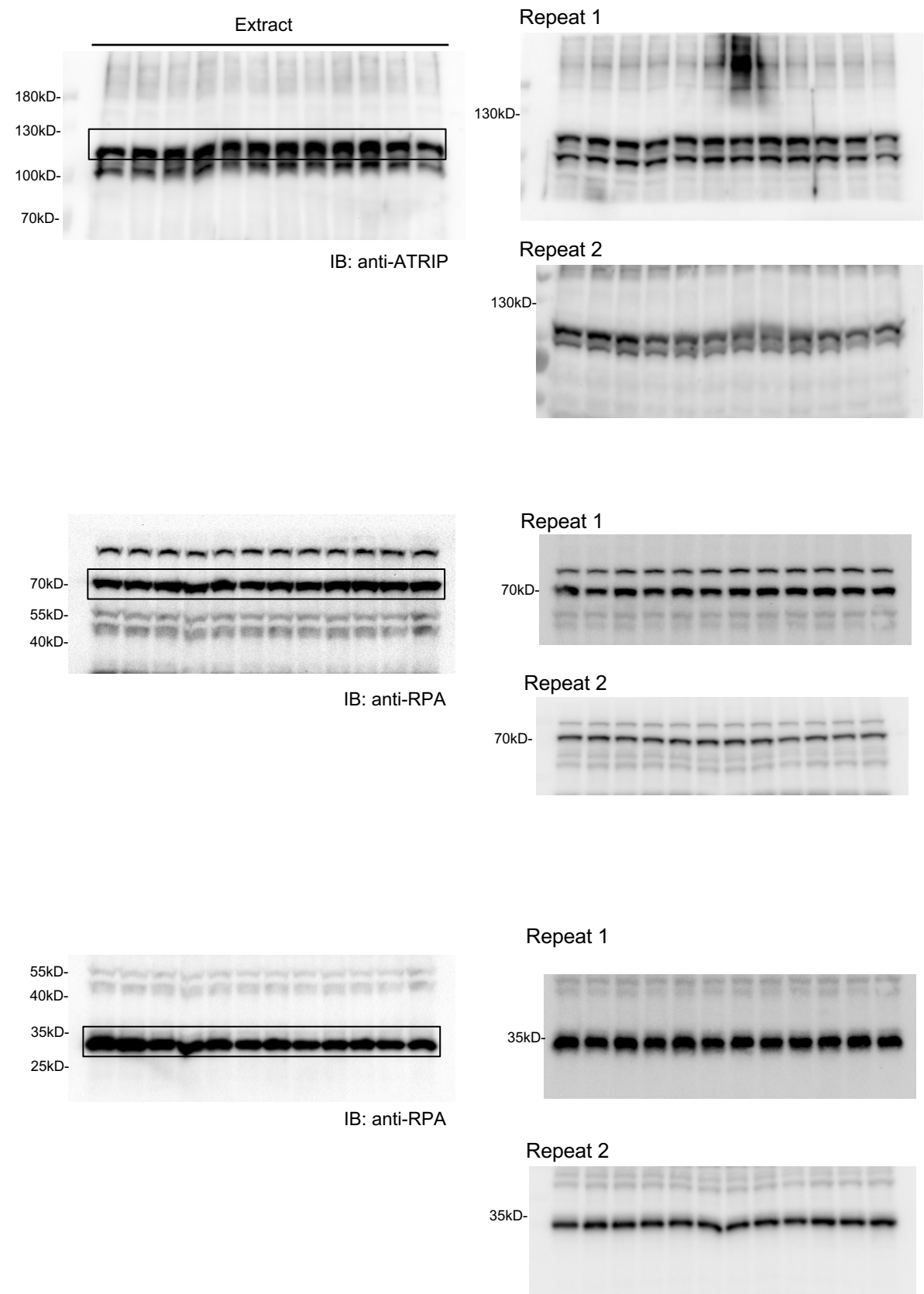

Figure 4E

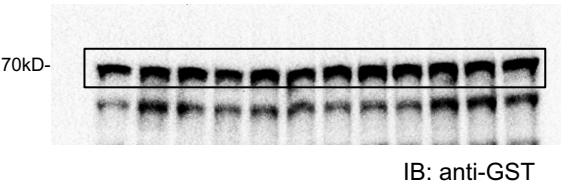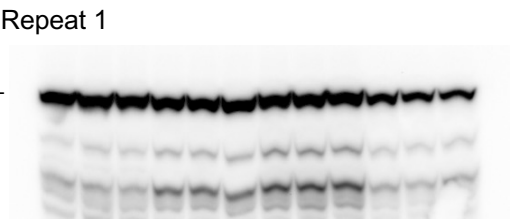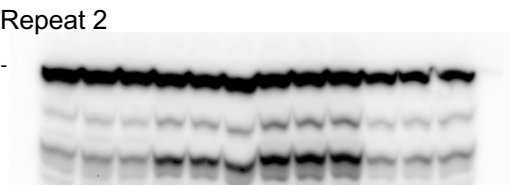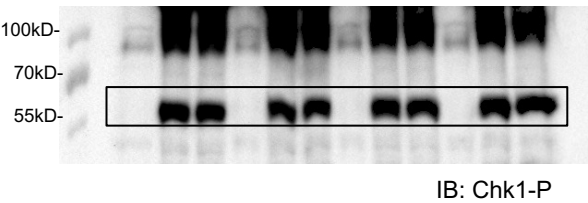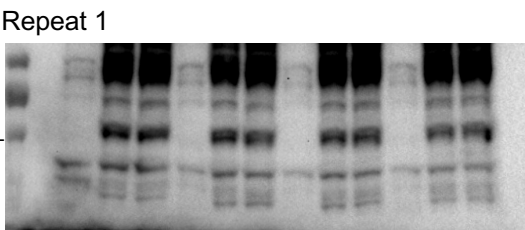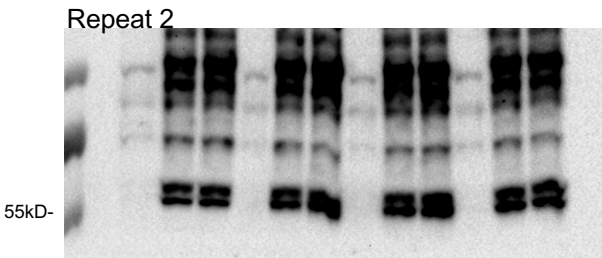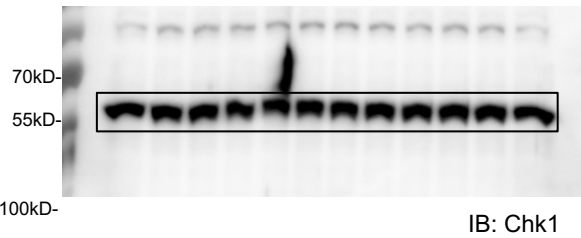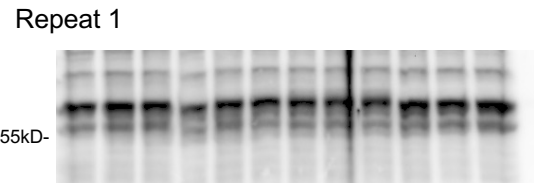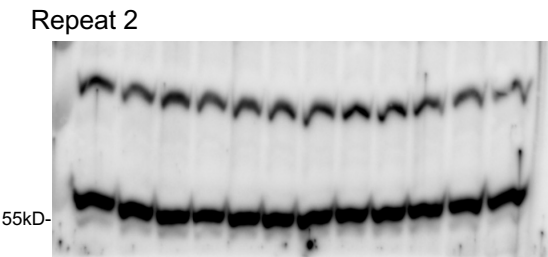

Supplement: Figure 4—source data 4. [file elife-82324-fig4-data4.zip › Figure 4-source data 4/IB-data-Figure 4E.pdf]

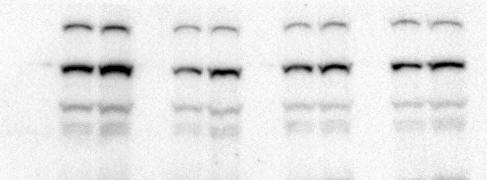

Supplement: Figure 4—source data 4. [file elife-82324-fig4-data4.zip › Figure 4-source data 4/Figure 4E initial trial/Bead-bound-RPA70.tif]

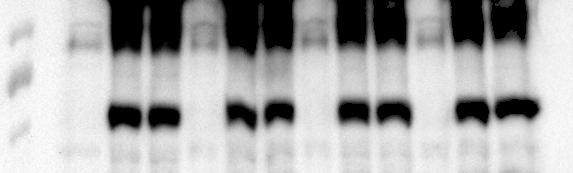

Supplement: Figure 4—source data 4. [file elife-82324-fig4-data4.zip › Figure 4-source data 4/Figure 4E initial trial/Extract-Chk1-P.tif]

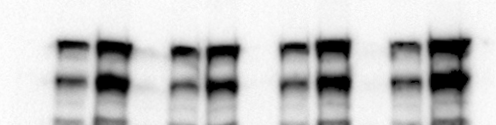

Supplement: Figure 4—source data 4. [file elife-82324-fig4-data4.zip › Figure 4-source data 4/Figure 4E initial trial/Bead-bound-GST.tif]

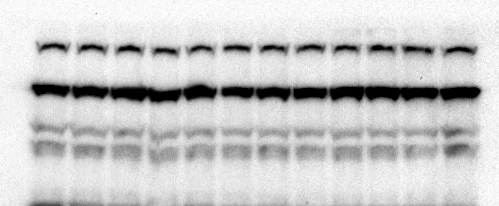

Supplement: Figure 4—source data 4. [file elife-82324-fig4-data4.zip › Figure 4-source data 4/Figure 4E initial trial/Extract-RPA70.tif]

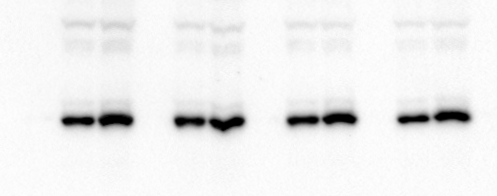

Supplement: Figure 4—source data 4. [file elife-82324-fig4-data4.zip › Figure 4-source data 4/Figure 4E initial trial/Bead-bound-RPA32.tif]

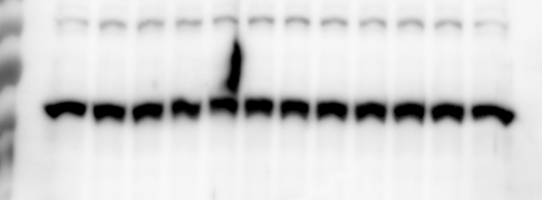

Supplement: Figure 4—source data 4. [file elife-82324-fig4-data4.zip › Figure 4-source data 4/Figure 4E initial trial/Extract-Chk1.tif]

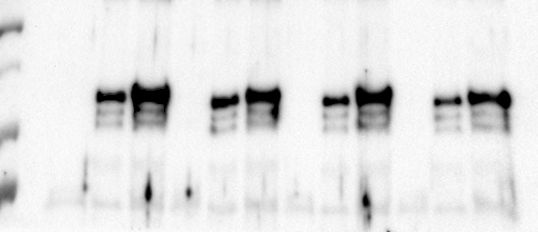

Supplement: Figure 4—source data 4. [file elife-82324-fig4-data4.zip › Figure 4-source data 4/Figure 4E initial trial/Bead-bound-ATRIP.tif]

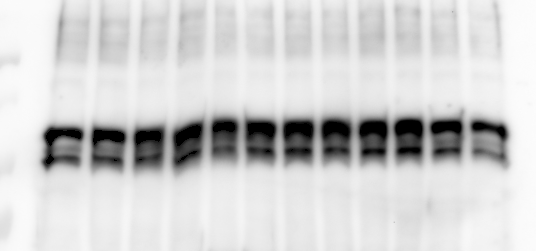

Supplement: Figure 4—source data 4. [file elife-82324-fig4-data4.zip › Figure 4-source data 4/Figure 4E initial trial/Extract-ATRIP.tif]

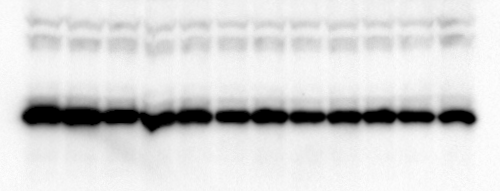

Supplement: Figure 4—source data 4. [file elife-82324-fig4-data4.zip › Figure 4-source data 4/Figure 4E initial trial/Extract-RPA32.tif]

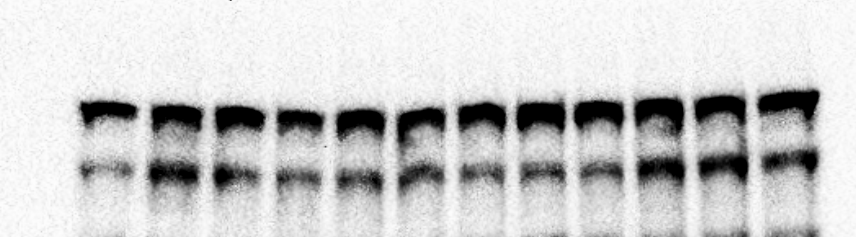

Supplement: Figure 4—source data 4. [file elife-82324-fig4-data4.zip › Figure 4-source data 4/Figure 4E initial trial/Extract-GST.tif]

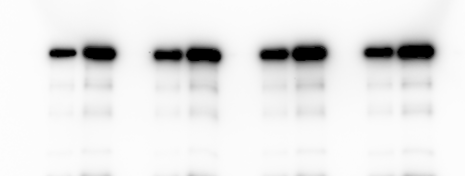

Supplement: Figure 4—source data 4. [file elife-82324-fig4-data4.zip › Figure 4-source data 4/Figure 4E Repeat1/Bead-bound-RPA70.tif]

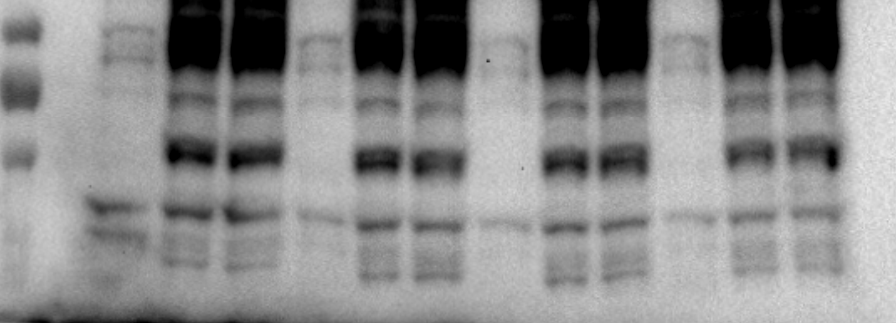

Supplement: Figure 4—source data 4. [file elife-82324-fig4-data4.zip › Figure 4-source data 4/Figure 4E Repeat1/Extract-Chk1-P.tif]

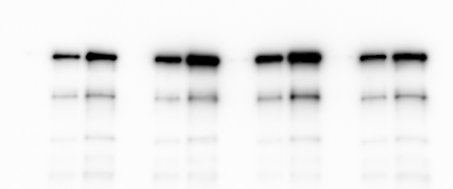

Supplement: Figure 4—source data 4. [file elife-82324-fig4-data4.zip › Figure 4-source data 4/Figure 4E Repeat1/Bead-bound-GST.tif]

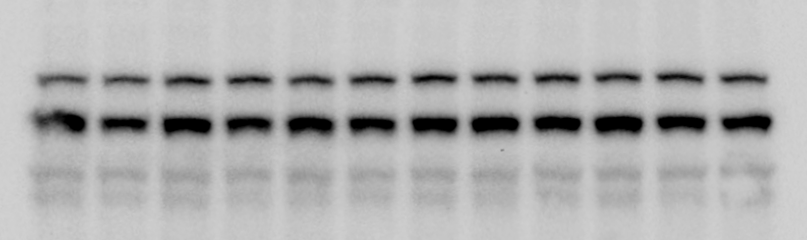

Supplement: Figure 4—source data 4. [file elife-82324-fig4-data4.zip › Figure 4-source data 4/Figure 4E Repeat1/Extract-RPA70.tif]

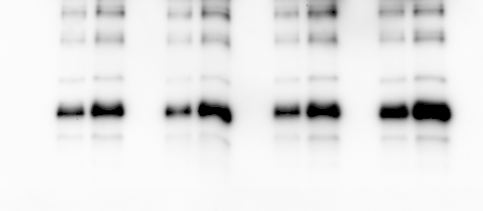

Supplement: Figure 4—source data 4. [file elife-82324-fig4-data4.zip › Figure 4-source data 4/Figure 4E Repeat1/Bead-bound-RPA32.tif]

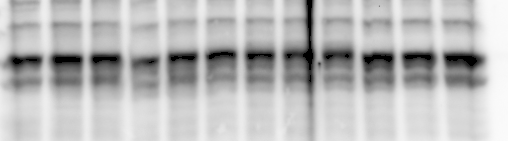

Supplement: Figure 4—source data 4. [file elife-82324-fig4-data4.zip › Figure 4-source data 4/Figure 4E Repeat1/Extract-Chk1.tif]

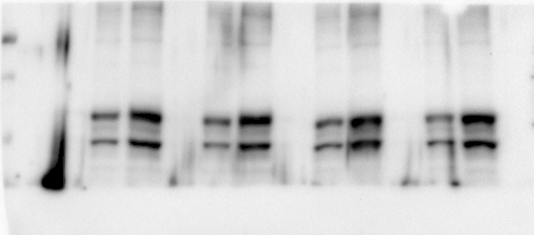

Supplement: Figure 4—source data 4. [file elife-82324-fig4-data4.zip › Figure 4-source data 4/Figure 4E Repeat1/Bead-bound-ATRIP.tif]
