# Supplementary material for: APE1 recruits ATRIP to ssDNA in an RPA-dependent and -independent manner to promote the ATR DNA damage response
Source: eLife. 2023 May 22;12:e82324. doi: 10.7554/eLife.82324 (PMC10202453; doi:10.7554/eLife.82324)

Figure 3-figure supplement 1

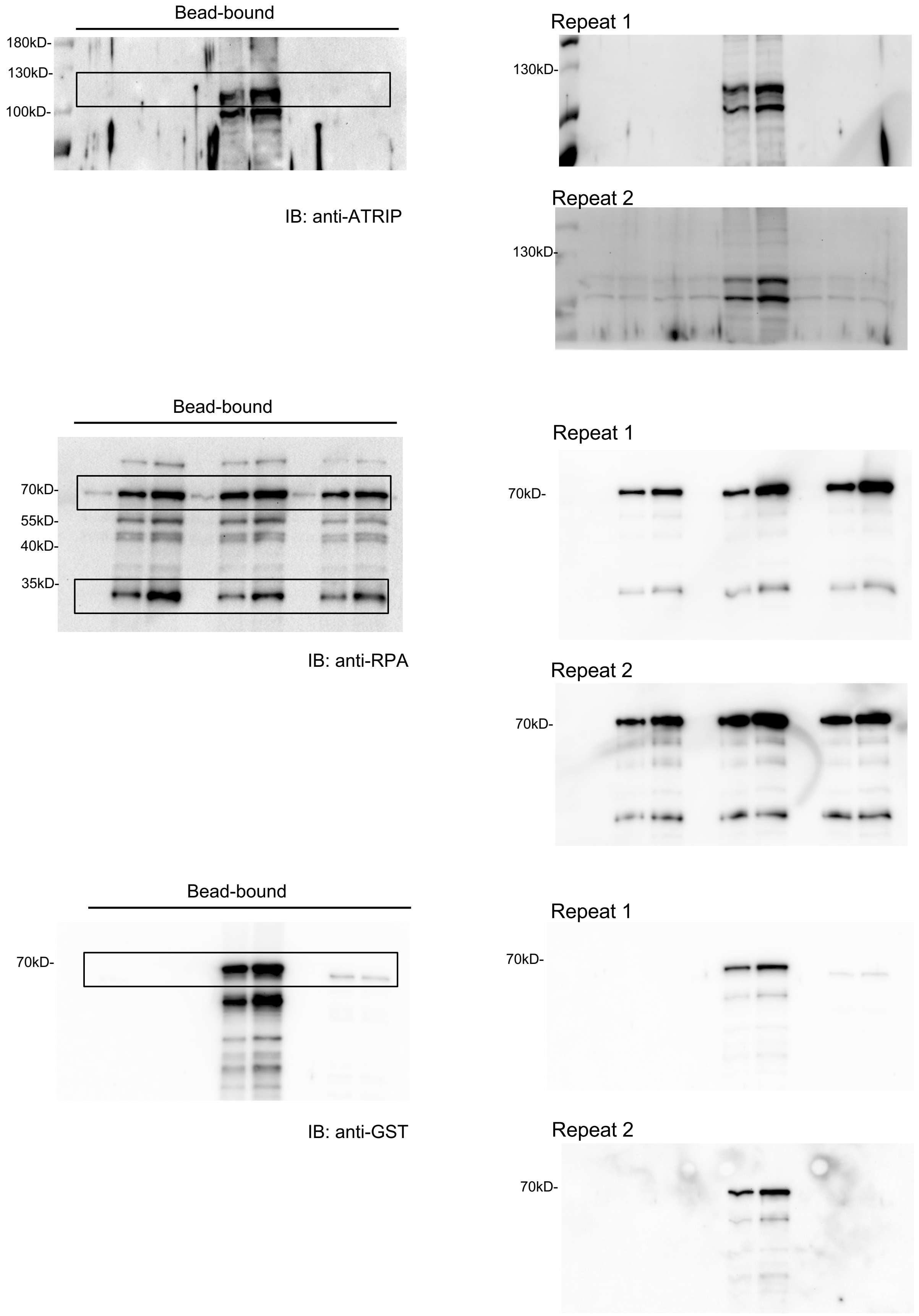

Figure 3-figure supplement 1

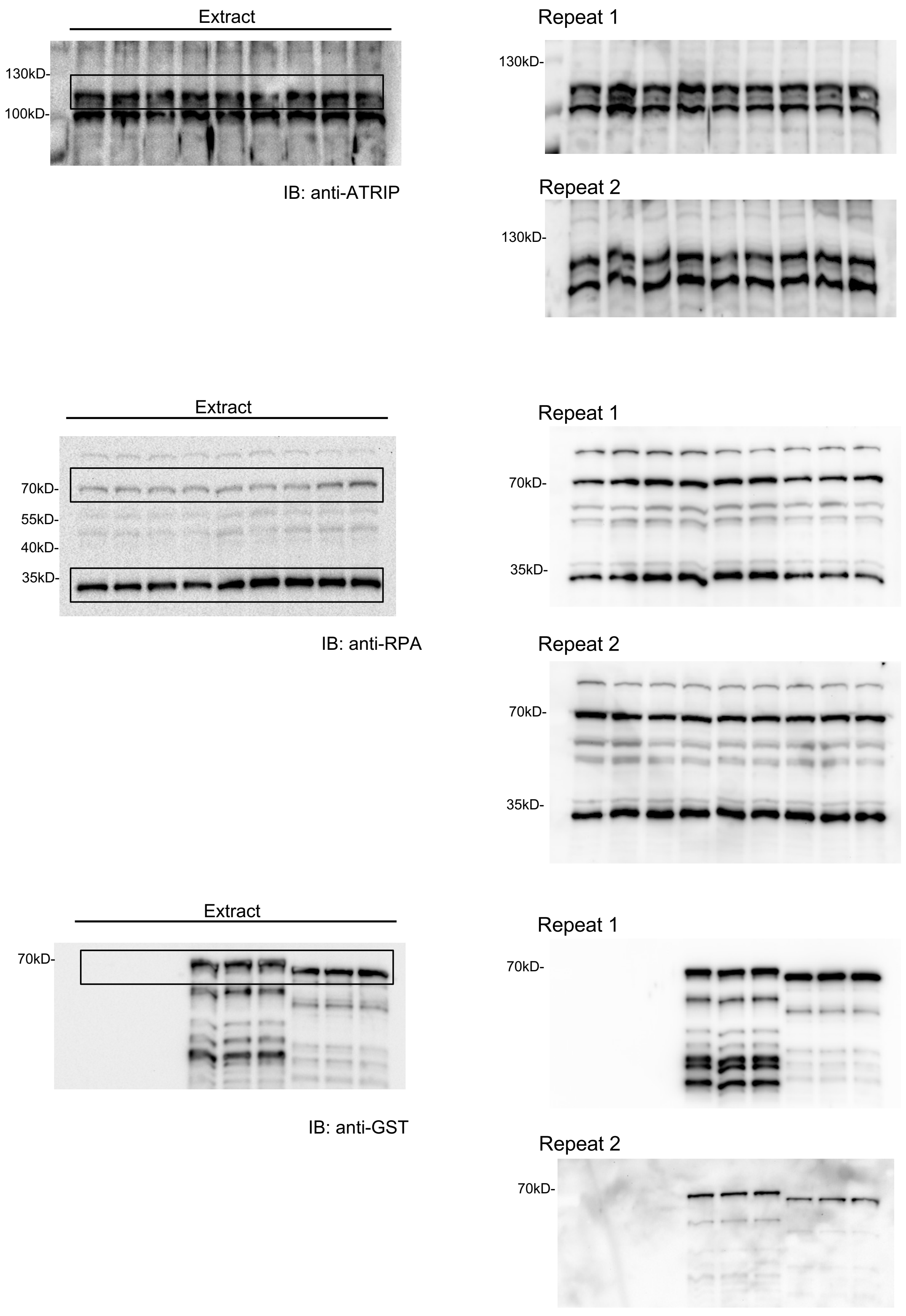

Figure 3-figure supplement 1

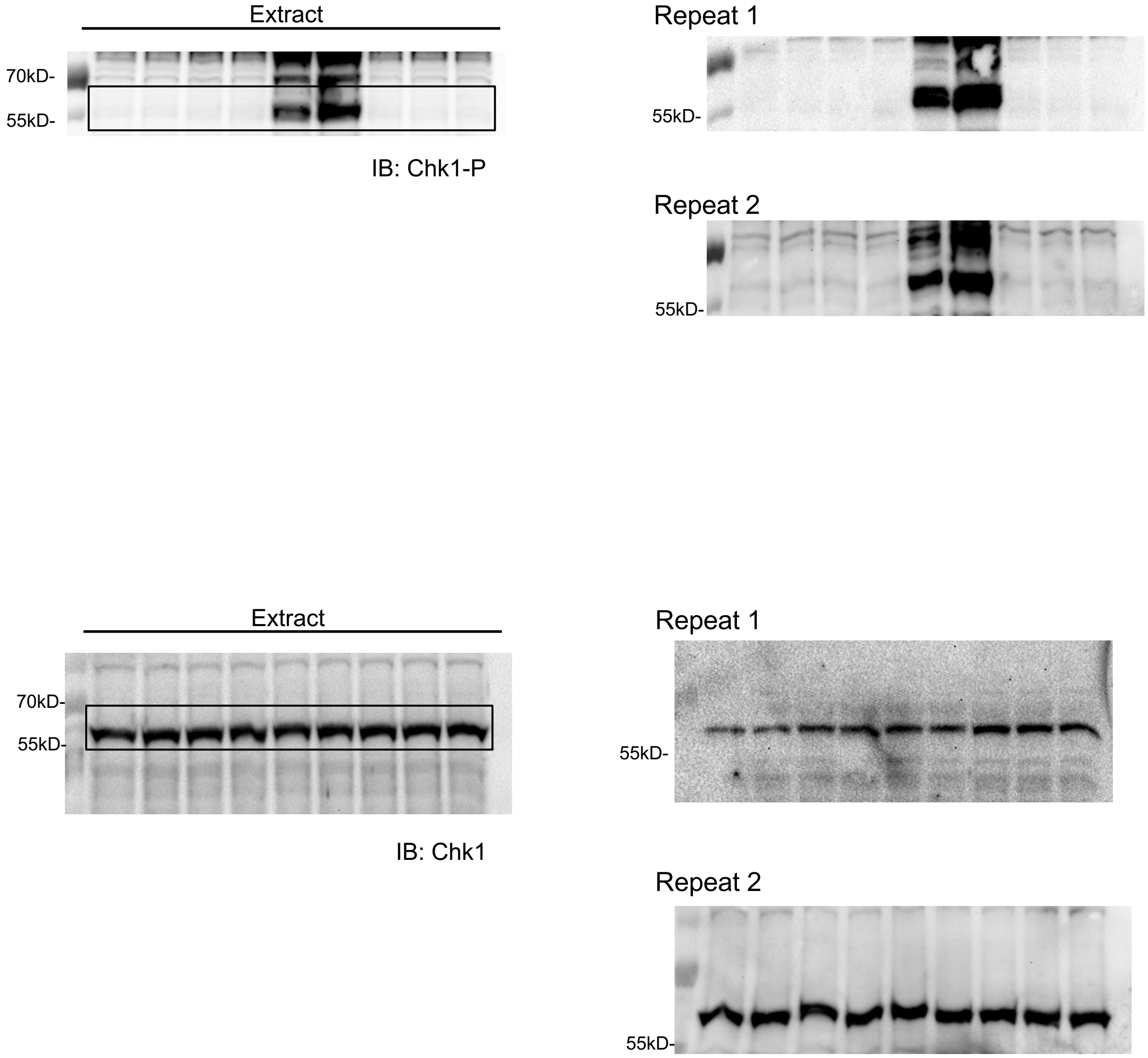

Supplement: Figure 3—figure supplement 1—source data 1. [file elife-82324-fig3-figsupp1-data1.zip › Figure 3-figure supplement 1-souce data 1/IB-data-Figure 3S1.pdf]
