# Supplementary figures and images for: APE1 recruits ATRIP to ssDNA in an RPA-dependent and -independent manner to promote the ATR DNA damage response (part 4 of 4)
Source: eLife. 2023 May 22;12:e82324. doi: 10.7554/eLife.82324 (PMC10202453; doi:10.7554/eLife.82324)

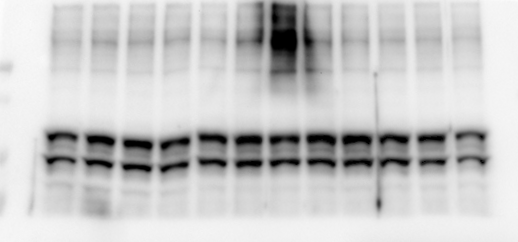

Supplement: Figure 4—source data 4. [file elife-82324-fig4-data4.zip › Figure 4-source data 4/Figure 4E Repeat1/Extract-ATRIP.tif]

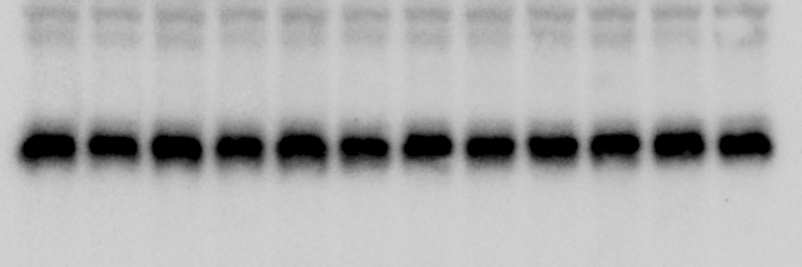

Supplement: Figure 4—source data 4. [file elife-82324-fig4-data4.zip › Figure 4-source data 4/Figure 4E Repeat1/Extract-RPA32.tif]

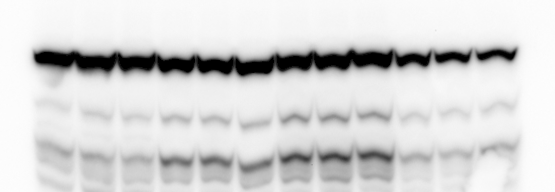

Supplement: Figure 4—source data 4. [file elife-82324-fig4-data4.zip › Figure 4-source data 4/Figure 4E Repeat1/Extract-GST.tif]

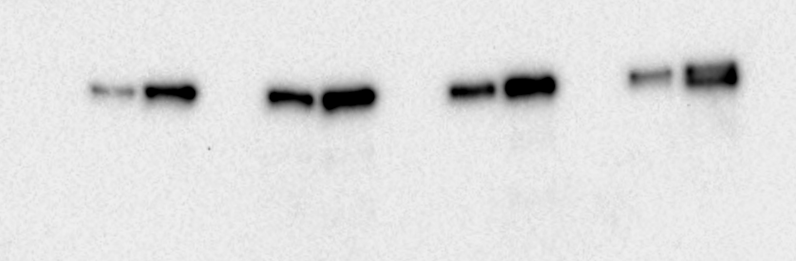

Supplement: Figure 4—source data 4. [file elife-82324-fig4-data4.zip › Figure 4-source data 4/Figure 4E Repeat2/Bead-bound-RPA70.tif]

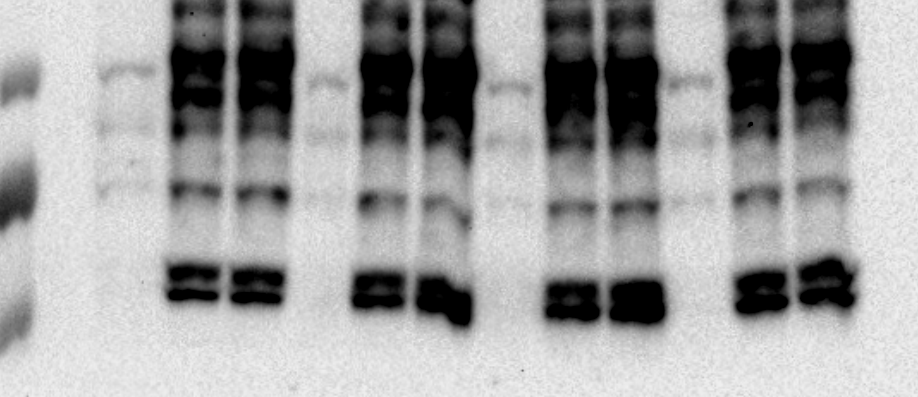

Supplement: Figure 4—source data 4. [file elife-82324-fig4-data4.zip › Figure 4-source data 4/Figure 4E Repeat2/Extract-Chk1-P.tif]

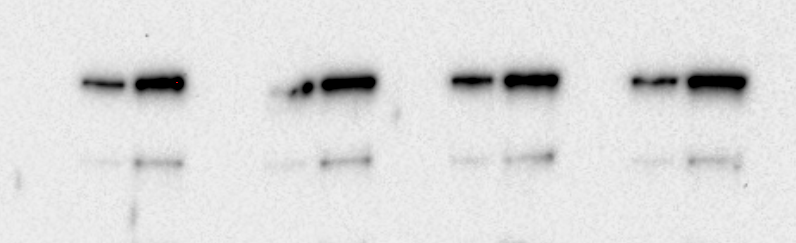

Supplement: Figure 4—source data 4. [file elife-82324-fig4-data4.zip › Figure 4-source data 4/Figure 4E Repeat2/Bead-bound-GST.tif]

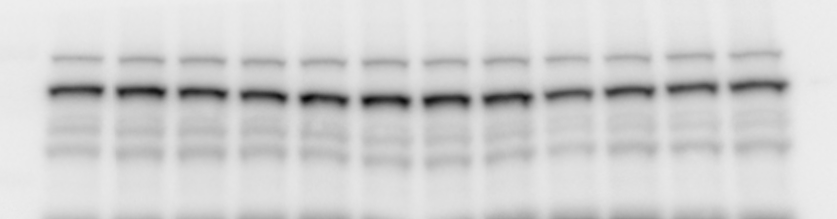

Supplement: Figure 4—source data 4. [file elife-82324-fig4-data4.zip › Figure 4-source data 4/Figure 4E Repeat2/Extract-RPA70.tif]

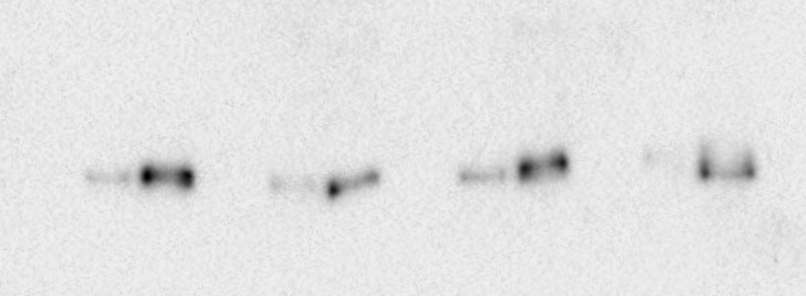

Supplement: Figure 4—source data 4. [file elife-82324-fig4-data4.zip › Figure 4-source data 4/Figure 4E Repeat2/Bead-bound-RPA32.tif]

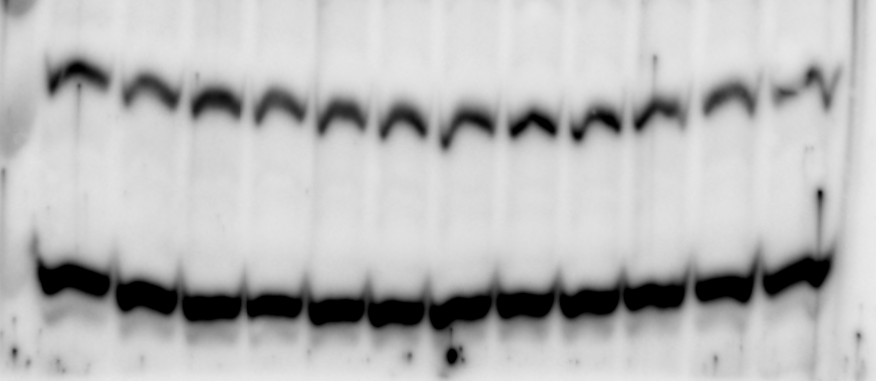

Supplement: Figure 4—source data 4. [file elife-82324-fig4-data4.zip › Figure 4-source data 4/Figure 4E Repeat2/Extract-Chk1.tif]

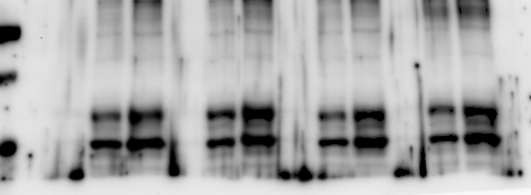

Supplement: Figure 4—source data 4. [file elife-82324-fig4-data4.zip › Figure 4-source data 4/Figure 4E Repeat2/Bead-bound-ATRIP.tif]

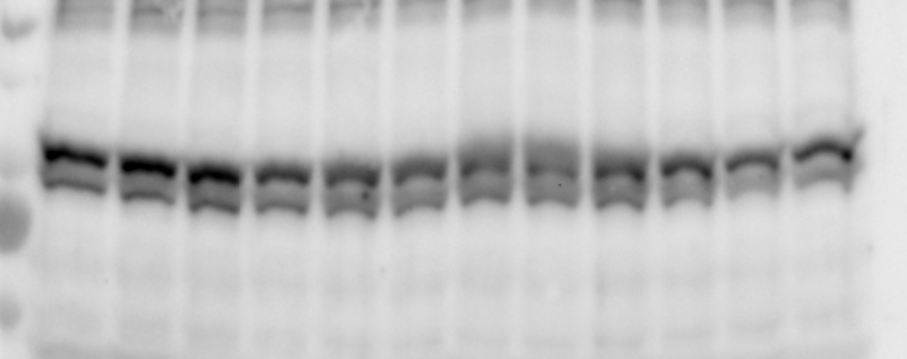

Supplement: Figure 4—source data 4. [file elife-82324-fig4-data4.zip › Figure 4-source data 4/Figure 4E Repeat2/Extract-ATRIP.tif]

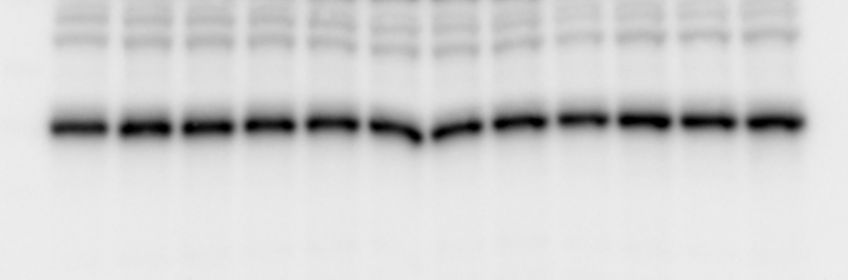

Supplement: Figure 4—source data 4. [file elife-82324-fig4-data4.zip › Figure 4-source data 4/Figure 4E Repeat2/Extract-RPA32.tif]

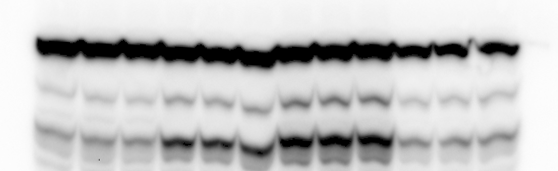

Supplement: Figure 4—source data 4. [file elife-82324-fig4-data4.zip › Figure 4-source data 4/Figure 4E Repeat2/Extract-GST.tif]

Figure 4-figure supplement 1B

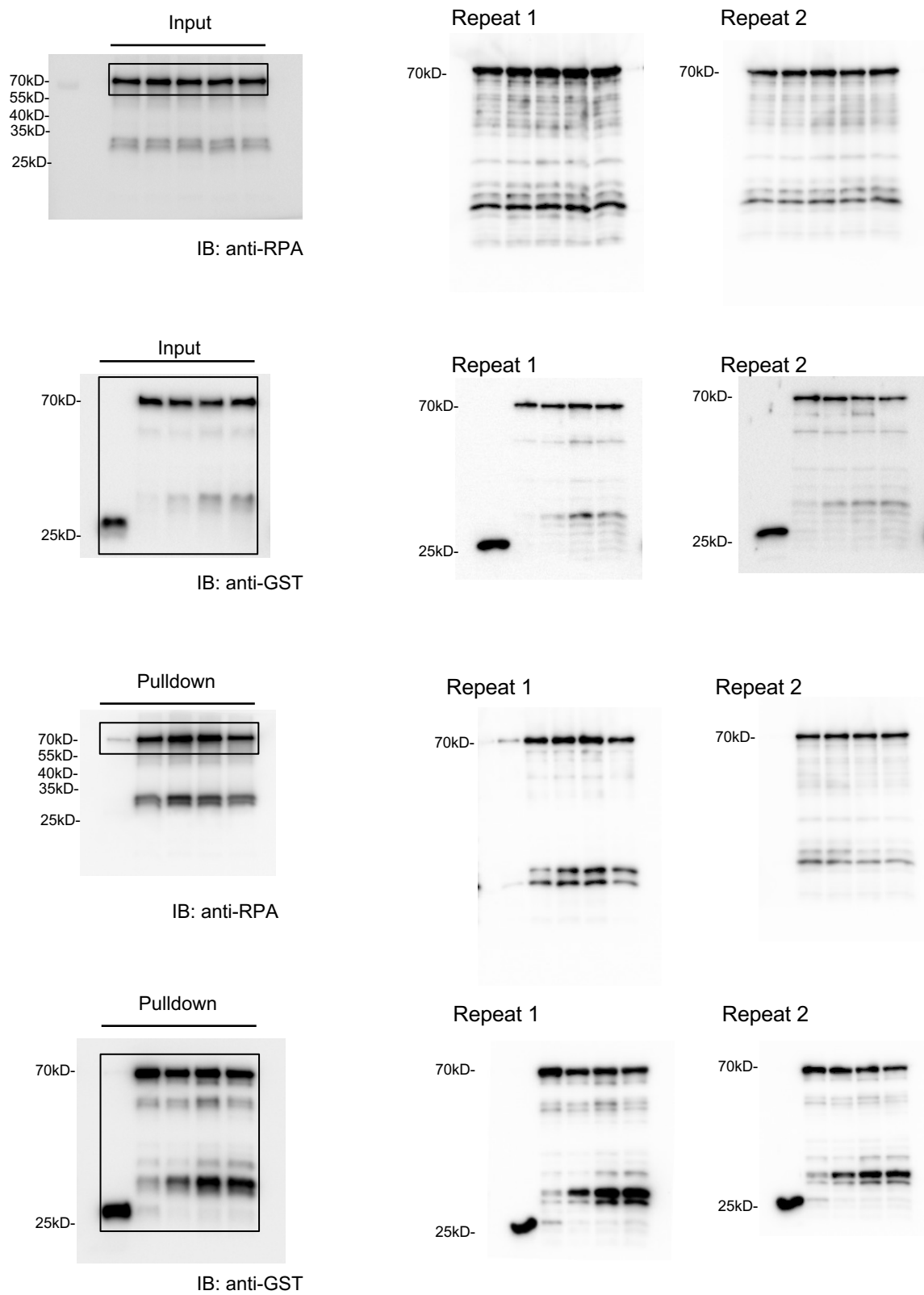

Supplement: Figure 4—figure supplement 1—source data 1. [file elife-82324-fig4-figsupp1-data1.zip › Figure 4-figure supplement 1-souce data 1/IB-data-Figure 4S1B.pdf]

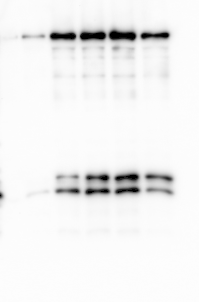

Supplement: Figure 4—figure supplement 1—source data 1. [file elife-82324-fig4-figsupp1-data1.zip › Figure 4-figure supplement 1-souce data 1/Figure 4S1B Repeat1/Pulldown-RPA.tif]

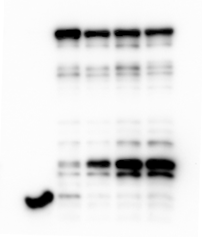

Supplement: Figure 4—figure supplement 1—source data 1. [file elife-82324-fig4-figsupp1-data1.zip › Figure 4-figure supplement 1-souce data 1/Figure 4S1B Repeat1/Pulldown-GST.tif]

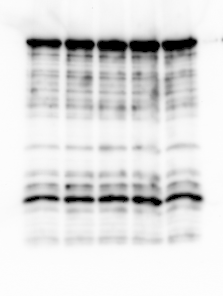

Supplement: Figure 4—figure supplement 1—source data 1. [file elife-82324-fig4-figsupp1-data1.zip › Figure 4-figure supplement 1-souce data 1/Figure 4S1B Repeat1/Input-RPA.tif]

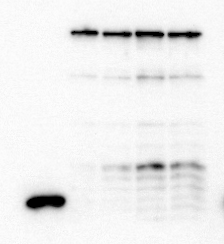

Supplement: Figure 4—figure supplement 1—source data 1. [file elife-82324-fig4-figsupp1-data1.zip › Figure 4-figure supplement 1-souce data 1/Figure 4S1B Repeat1/Input-GST.tif]

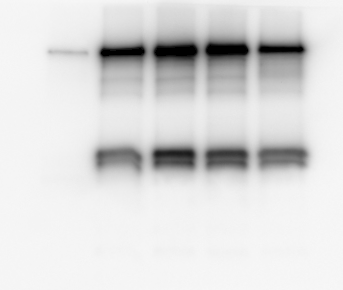

Supplement: Figure 4—figure supplement 1—source data 1. [file elife-82324-fig4-figsupp1-data1.zip › Figure 4-figure supplement 1-souce data 1/Figure 4S1B initial/Pulldown-RPA.tif]

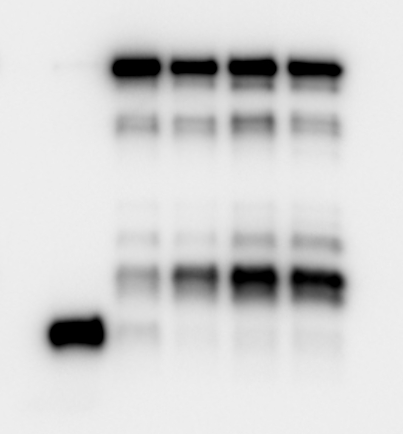

Supplement: Figure 4—figure supplement 1—source data 1. [file elife-82324-fig4-figsupp1-data1.zip › Figure 4-figure supplement 1-souce data 1/Figure 4S1B initial/Pulldown-GST.tif]

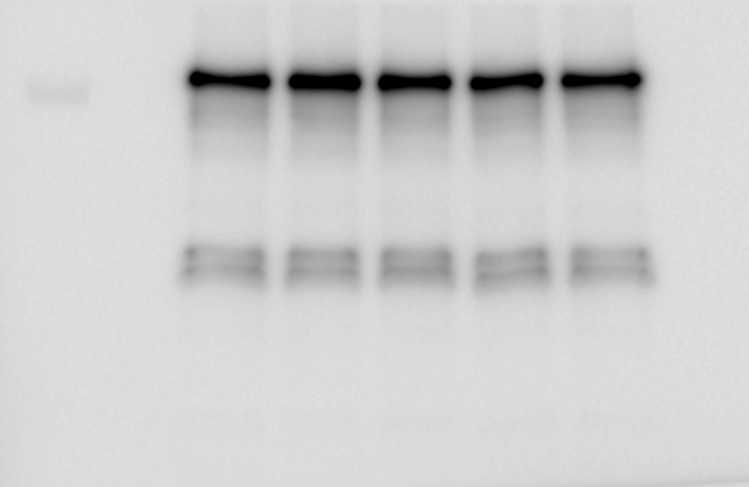

Supplement: Figure 4—figure supplement 1—source data 1. [file elife-82324-fig4-figsupp1-data1.zip › Figure 4-figure supplement 1-souce data 1/Figure 4S1B initial/Input-RPA.tif]

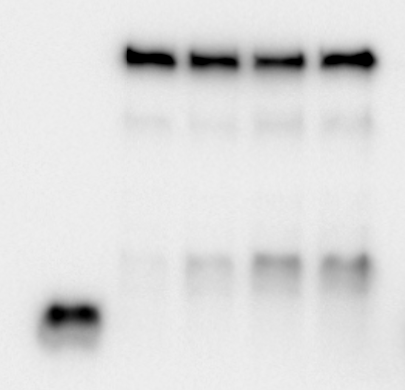

Supplement: Figure 4—figure supplement 1—source data 1. [file elife-82324-fig4-figsupp1-data1.zip › Figure 4-figure supplement 1-souce data 1/Figure 4S1B initial/Input-GST.tif]

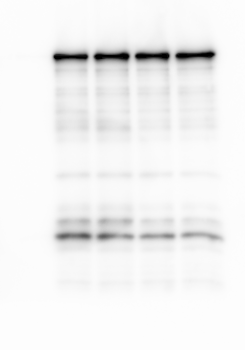

Supplement: Figure 4—figure supplement 1—source data 1. [file elife-82324-fig4-figsupp1-data1.zip › Figure 4-figure supplement 1-souce data 1/Figure 4S1B Repeat2/Pulldown-RPA.tif]

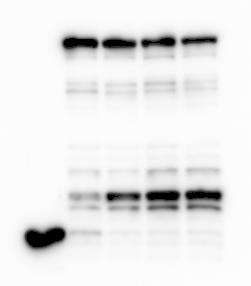

Supplement: Figure 4—figure supplement 1—source data 1. [file elife-82324-fig4-figsupp1-data1.zip › Figure 4-figure supplement 1-souce data 1/Figure 4S1B Repeat2/Pulldown-GST.tif]

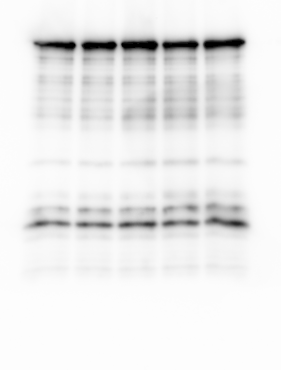

Supplement: Figure 4—figure supplement 1—source data 1. [file elife-82324-fig4-figsupp1-data1.zip › Figure 4-figure supplement 1-souce data 1/Figure 4S1B Repeat2/Input-RPA.tif]

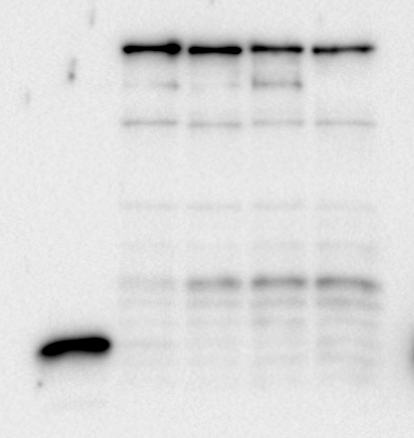

Supplement: Figure 4—figure supplement 1—source data 1. [file elife-82324-fig4-figsupp1-data1.zip › Figure 4-figure supplement 1-souce data 1/Figure 4S1B Repeat2/Input-GST.tif]

Figure 4-figure supplement 3A

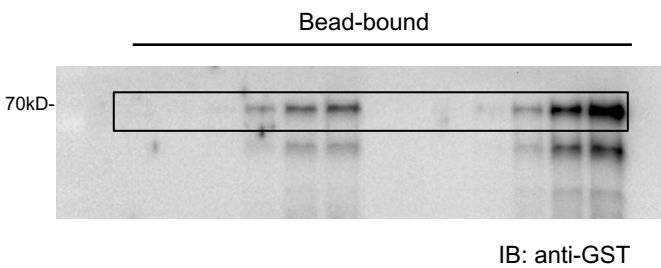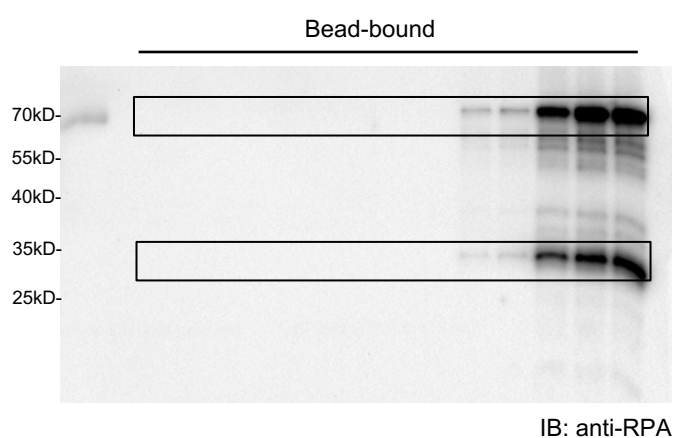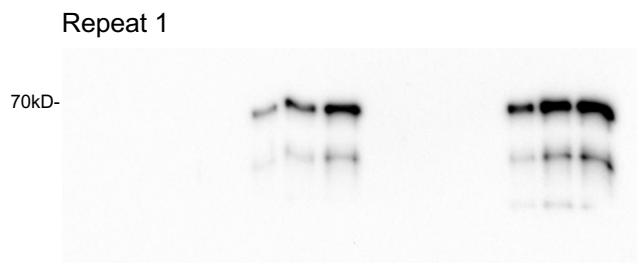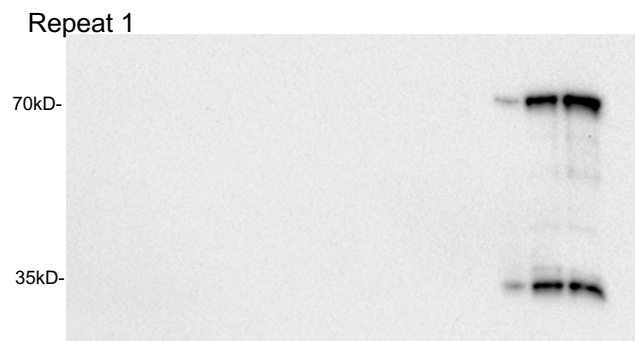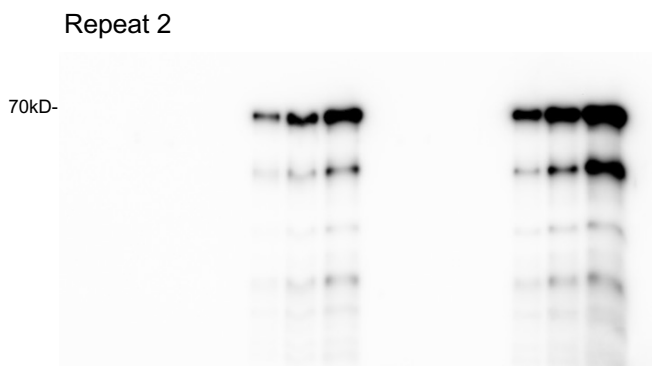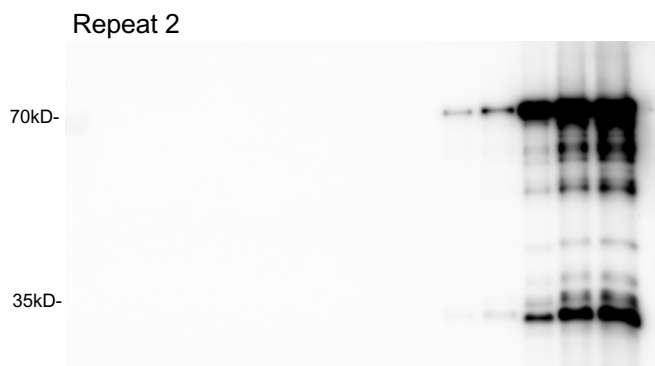

Figure 4-figure supplement 3A

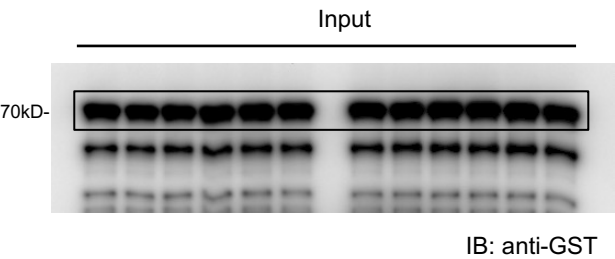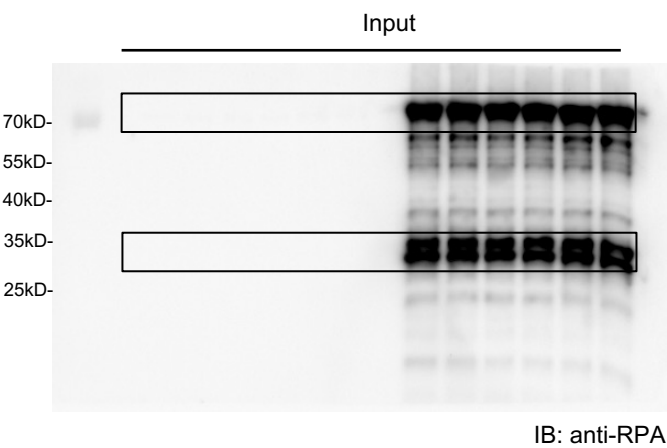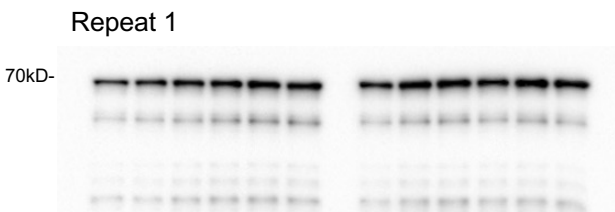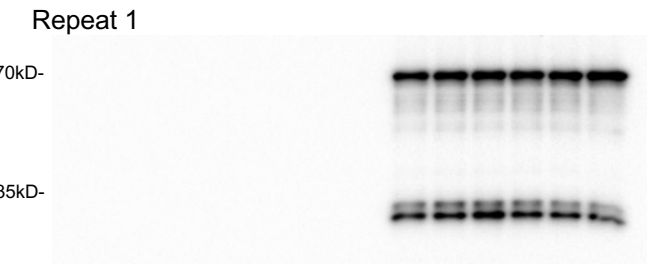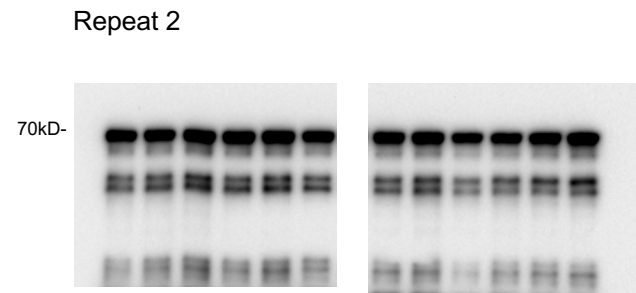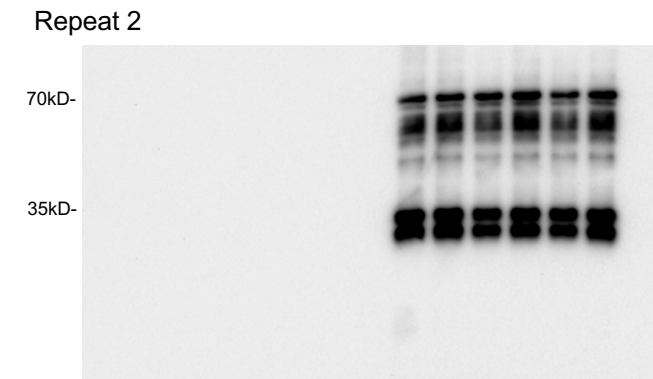

Supplement: Figure 4—figure supplement 3—source data 1. [file elife-82324-fig4-figsupp3-data1.zip › Figure 4-figure supplement 3-souce data 1/IB-data-Figure 4S3A.pdf]

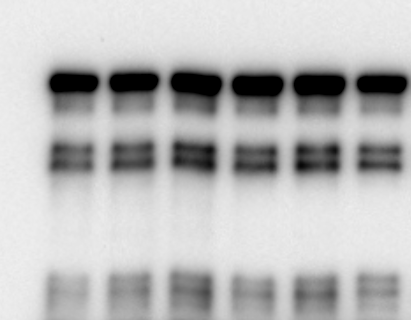

Supplement: Figure 4—figure supplement 3—source data 1. [file elife-82324-fig4-figsupp3-data1.zip › Figure 4-figure supplement 3-souce data 1/Figure 4S3A Repeat2/Input-GST-APE1.tif]

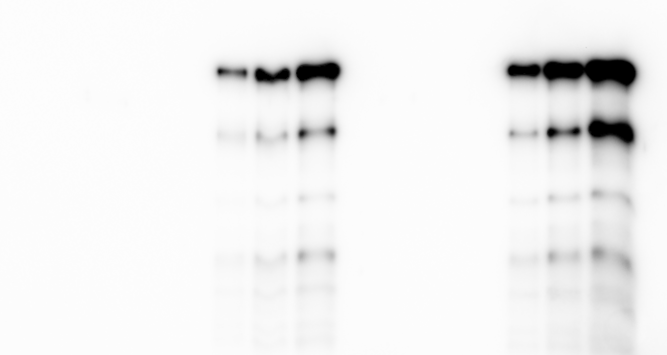

Supplement: Figure 4—figure supplement 3—source data 1. [file elife-82324-fig4-figsupp3-data1.zip › Figure 4-figure supplement 3-souce data 1/Figure 4S3A Repeat2/Bead-GST-APE1.tif]

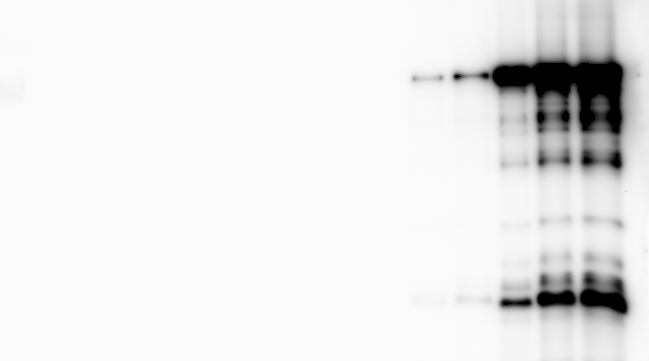

Supplement: Figure 4—figure supplement 3—source data 1. [file elife-82324-fig4-figsupp3-data1.zip › Figure 4-figure supplement 3-souce data 1/Figure 4S3A Repeat2/Bead-RPA.tif]

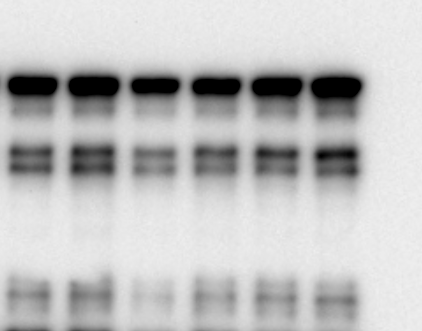

Supplement: Figure 4—figure supplement 3—source data 1. [file elife-82324-fig4-figsupp3-data1.zip › Figure 4-figure supplement 3-souce data 1/Figure 4S3A Repeat2/Input-GST-APE1-2.tif]

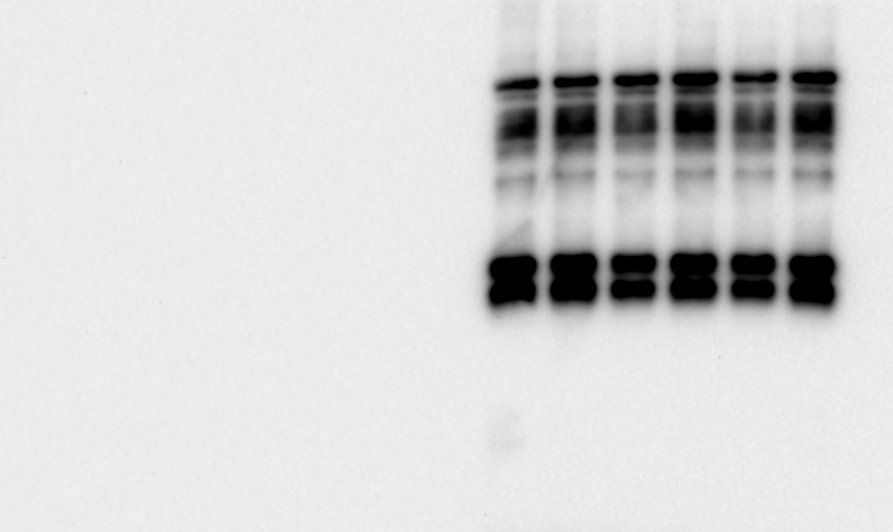

Supplement: Figure 4—figure supplement 3—source data 1. [file elife-82324-fig4-figsupp3-data1.zip › Figure 4-figure supplement 3-souce data 1/Figure 4S3A Repeat2/Input-RPA.tif]

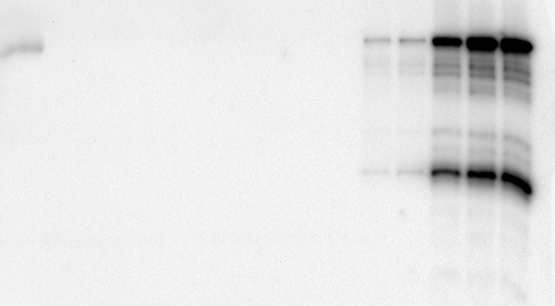

Supplement: Figure 4—figure supplement 3—source data 1. [file elife-82324-fig4-figsupp3-data1.zip › Figure 4-figure supplement 3-souce data 1/Figure 4S3A initial/Bead-RPA.tif]

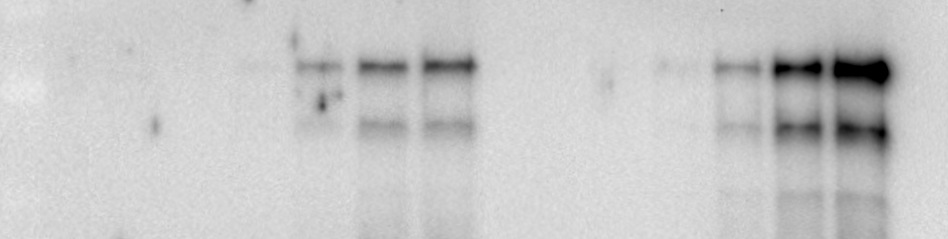

Supplement: Figure 4—figure supplement 3—source data 1. [file elife-82324-fig4-figsupp3-data1.zip › Figure 4-figure supplement 3-souce data 1/Figure 4S3A initial/Bead-APE1.tif]

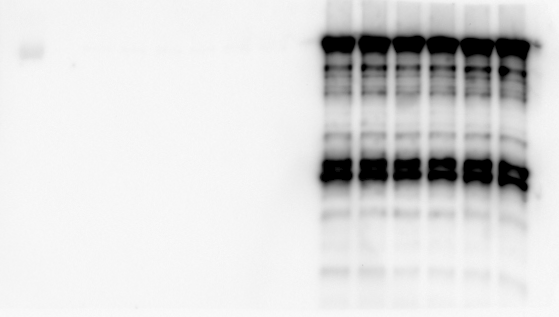

Supplement: Figure 4—figure supplement 3—source data 1. [file elife-82324-fig4-figsupp3-data1.zip › Figure 4-figure supplement 3-souce data 1/Figure 4S3A initial/Input-RPA.tif]

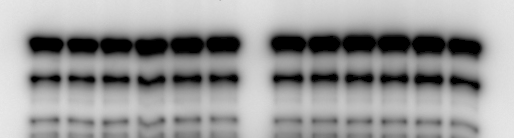

Supplement: Figure 4—figure supplement 3—source data 1. [file elife-82324-fig4-figsupp3-data1.zip › Figure 4-figure supplement 3-souce data 1/Figure 4S3A initial/Input-APE1.tif]

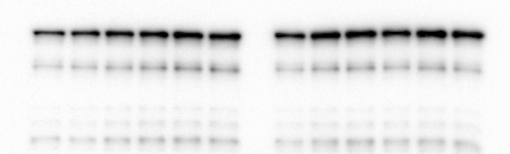

Supplement: Figure 4—figure supplement 3—source data 1. [file elife-82324-fig4-figsupp3-data1.zip › Figure 4-figure supplement 3-souce data 1/Figure 4S3A Repeat1/Input-GST-APE1.tif]

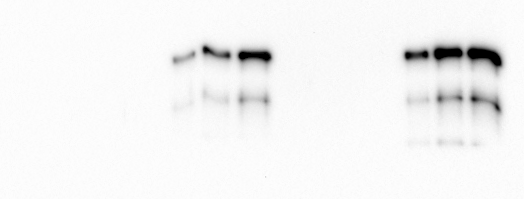

Supplement: Figure 4—figure supplement 3—source data 1. [file elife-82324-fig4-figsupp3-data1.zip › Figure 4-figure supplement 3-souce data 1/Figure 4S3A Repeat1/Bead-GST-APE1.tif]

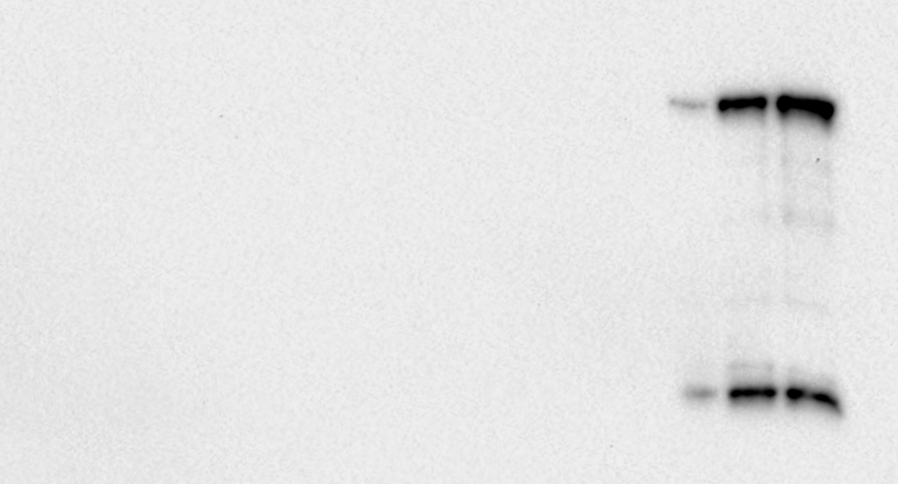

Supplement: Figure 4—figure supplement 3—source data 1. [file elife-82324-fig4-figsupp3-data1.zip › Figure 4-figure supplement 3-souce data 1/Figure 4S3A Repeat1/Bead-RPA.tif]

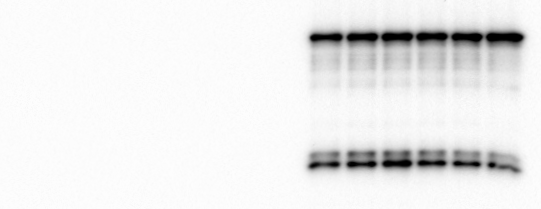

Supplement: Figure 4—figure supplement 3—source data 1. [file elife-82324-fig4-figsupp3-data1.zip › Figure 4-figure supplement 3-souce data 1/Figure 4S3A Repeat1/Input-RPA.tif]

Figure 4-figure supplement 3C

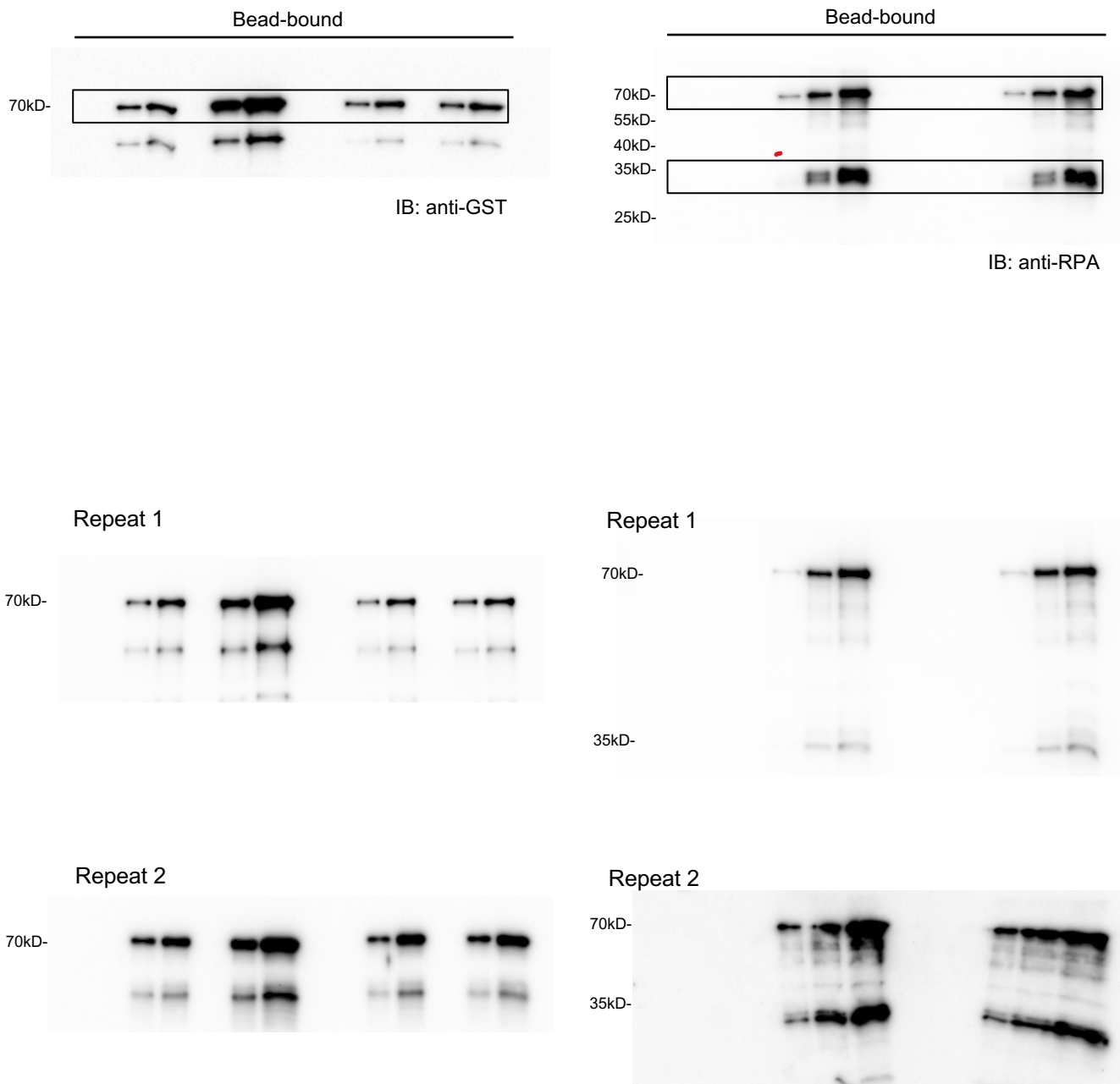

Figure 4-figure supplement 3C

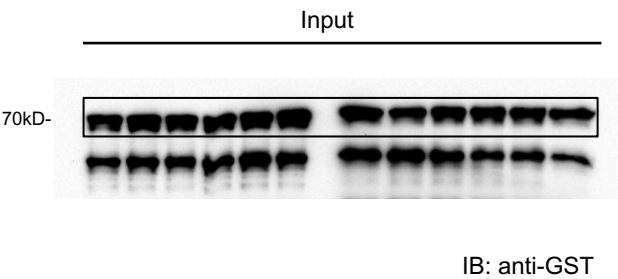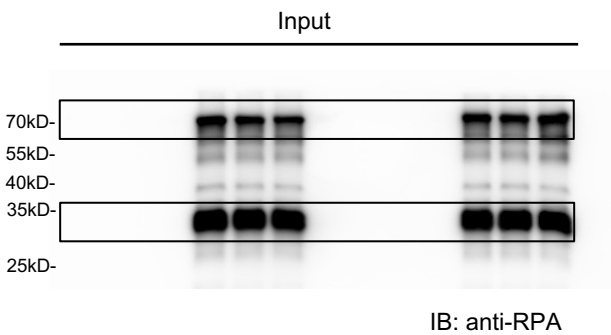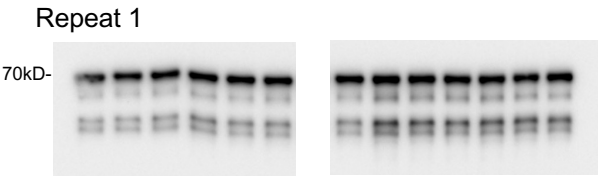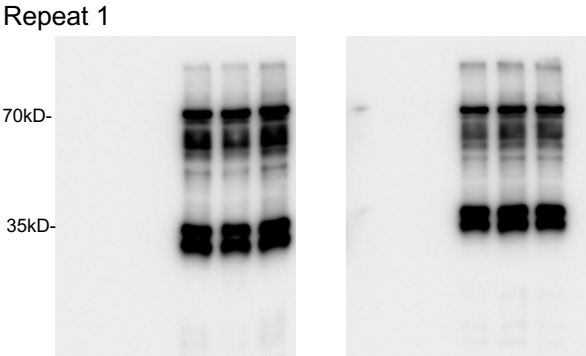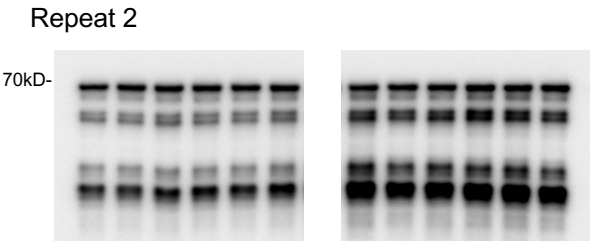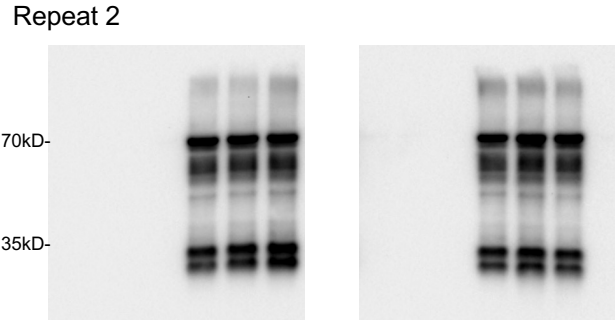

Supplement: Figure 4—figure supplement 3—source data 2. [file elife-82324-fig4-figsupp3-data2.zip › Figure 4-figure supplement 3-souce data 2/IB-data-Figure 4S3C.pdf]

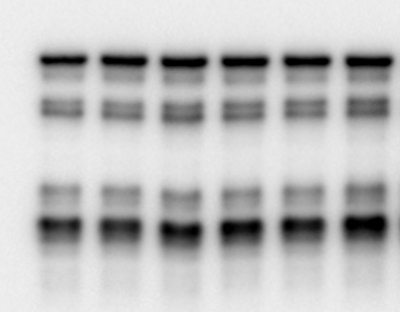

Supplement: Figure 4—figure supplement 3—source data 2. [file elife-82324-fig4-figsupp3-data2.zip › Figure 4-figure supplement 3-souce data 2/Figure 4S3C Repeat2/Input-GST-APE1.tif]

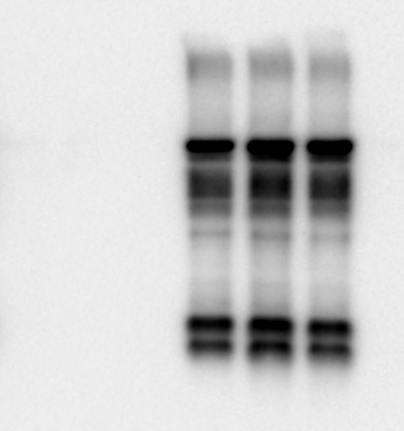

Supplement: Figure 4—figure supplement 3—source data 2. [file elife-82324-fig4-figsupp3-data2.zip › Figure 4-figure supplement 3-souce data 2/Figure 4S3C Repeat2/Input-RPA-2.tif]

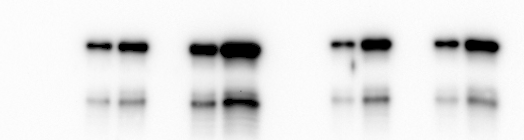

Supplement: Figure 4—figure supplement 3—source data 2. [file elife-82324-fig4-figsupp3-data2.zip › Figure 4-figure supplement 3-souce data 2/Figure 4S3C Repeat2/Bead-GST-APE1.tif]

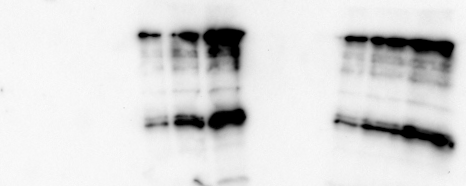

Supplement: Figure 4—figure supplement 3—source data 2. [file elife-82324-fig4-figsupp3-data2.zip › Figure 4-figure supplement 3-souce data 2/Figure 4S3C Repeat2/Bead-RPA.tif]

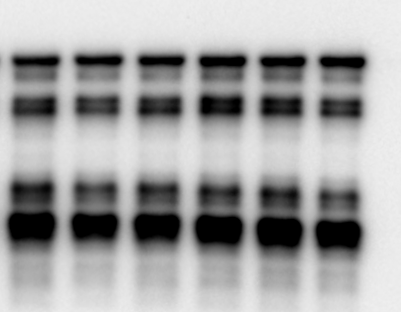

Supplement: Figure 4—figure supplement 3—source data 2. [file elife-82324-fig4-figsupp3-data2.zip › Figure 4-figure supplement 3-souce data 2/Figure 4S3C Repeat2/Input-GST-APE1-2.tif]

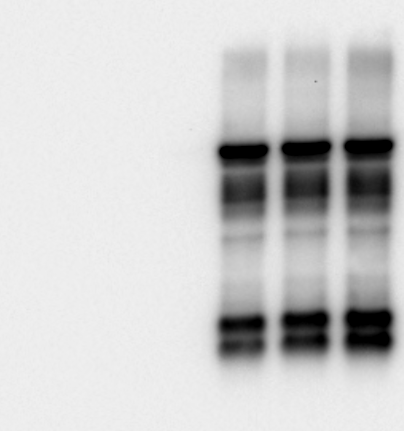

Supplement: Figure 4—figure supplement 3—source data 2. [file elife-82324-fig4-figsupp3-data2.zip › Figure 4-figure supplement 3-souce data 2/Figure 4S3C Repeat2/Input-RPA.tif]

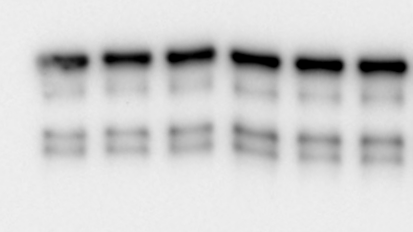

Supplement: Figure 4—figure supplement 3—source data 2. [file elife-82324-fig4-figsupp3-data2.zip › Figure 4-figure supplement 3-souce data 2/Figure 4S3C Repeat1/Input-GST-APE1.tif]

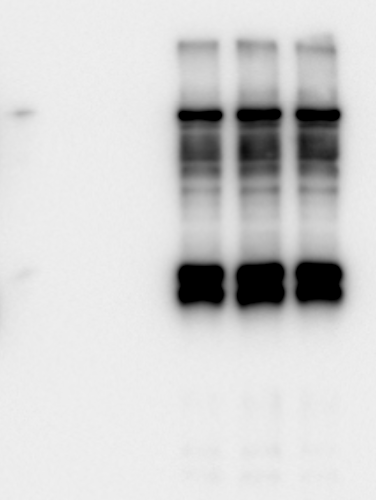

Supplement: Figure 4—figure supplement 3—source data 2. [file elife-82324-fig4-figsupp3-data2.zip › Figure 4-figure supplement 3-souce data 2/Figure 4S3C Repeat1/Input-RPA-2.tif]

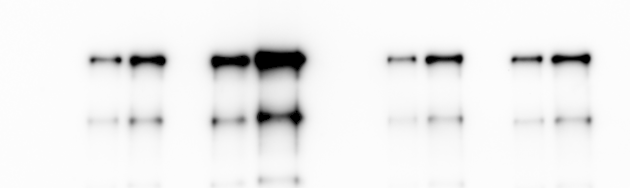

Supplement: Figure 4—figure supplement 3—source data 2. [file elife-82324-fig4-figsupp3-data2.zip › Figure 4-figure supplement 3-souce data 2/Figure 4S3C Repeat1/Bead-GST-APE1.tif]

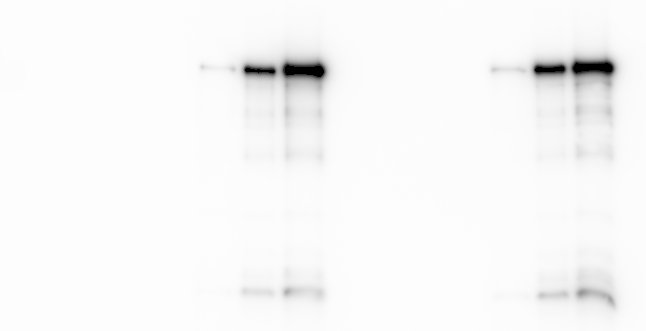

Supplement: Figure 4—figure supplement 3—source data 2. [file elife-82324-fig4-figsupp3-data2.zip › Figure 4-figure supplement 3-souce data 2/Figure 4S3C Repeat1/Bead-RPA.tif]

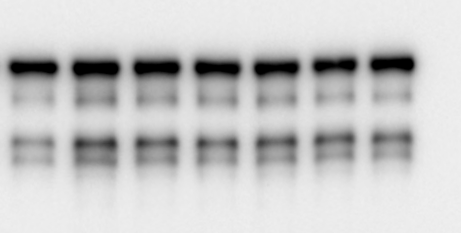

Supplement: Figure 4—figure supplement 3—source data 2. [file elife-82324-fig4-figsupp3-data2.zip › Figure 4-figure supplement 3-souce data 2/Figure 4S3C Repeat1/Input-GST-APE1-2.tif]

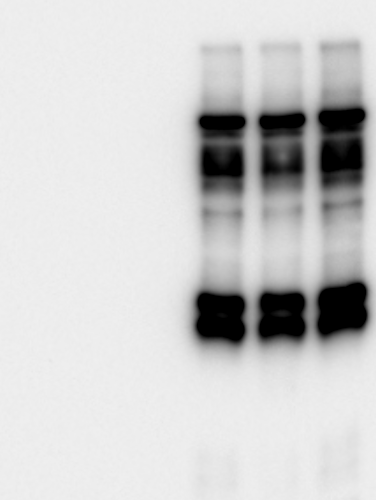

Supplement: Figure 4—figure supplement 3—source data 2. [file elife-82324-fig4-figsupp3-data2.zip › Figure 4-figure supplement 3-souce data 2/Figure 4S3C Repeat1/Input-RPA.tif]

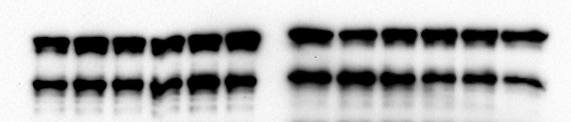

Supplement: Figure 4—figure supplement 3—source data 2. [file elife-82324-fig4-figsupp3-data2.zip › Figure 4-figure supplement 3-souce data 2/Figure 4S3C initial/Input-GST-APE1.tif]

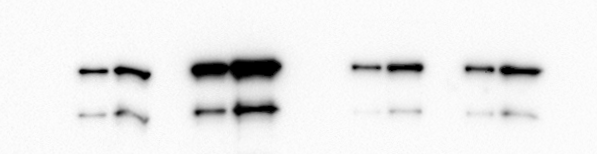

Supplement: Figure 4—figure supplement 3—source data 2. [file elife-82324-fig4-figsupp3-data2.zip › Figure 4-figure supplement 3-souce data 2/Figure 4S3C initial/Bead-GST-APE1.tif]

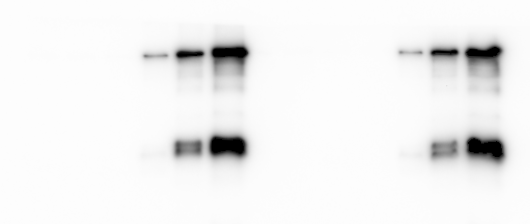

Supplement: Figure 4—figure supplement 3—source data 2. [file elife-82324-fig4-figsupp3-data2.zip › Figure 4-figure supplement 3-souce data 2/Figure 4S3C initial/Bead-RPA.tif]

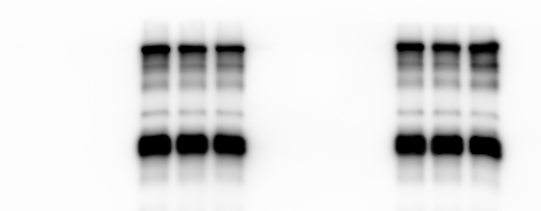

Supplement: Figure 4—figure supplement 3—source data 2. [file elife-82324-fig4-figsupp3-data2.zip › Figure 4-figure supplement 3-souce data 2/Figure 4S3C initial/Input-RPA.tif]

Figure 4-figure supplement 3E

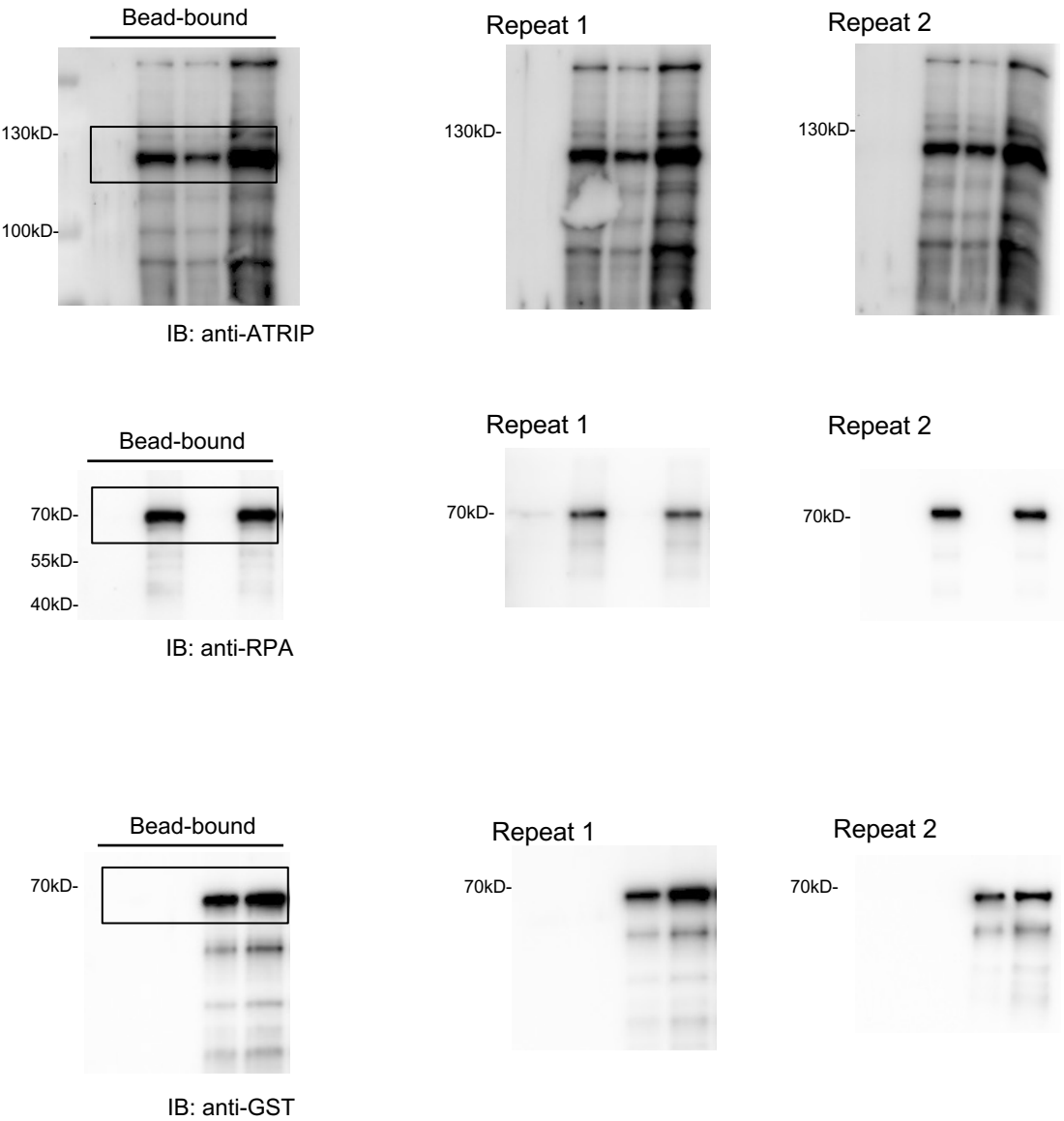

Figure 4-figure supplement 3E

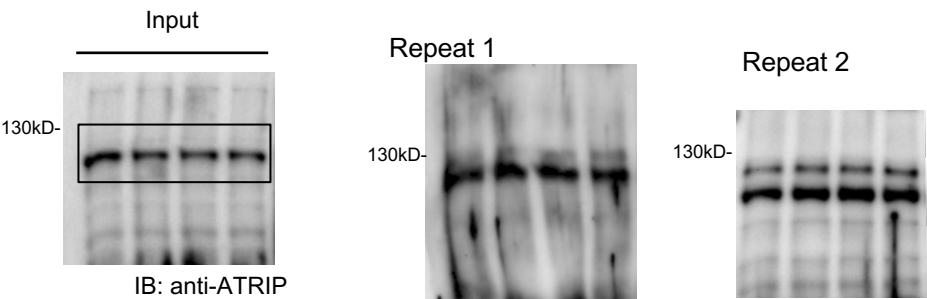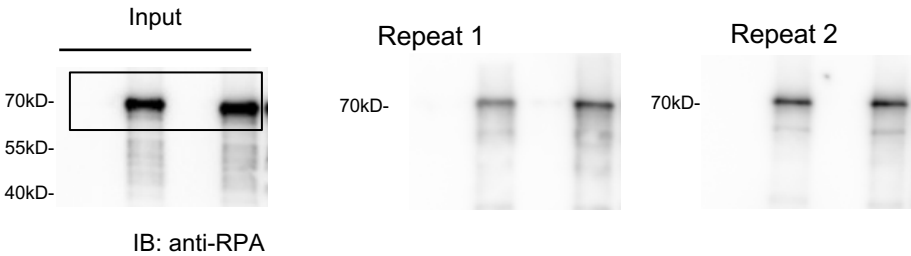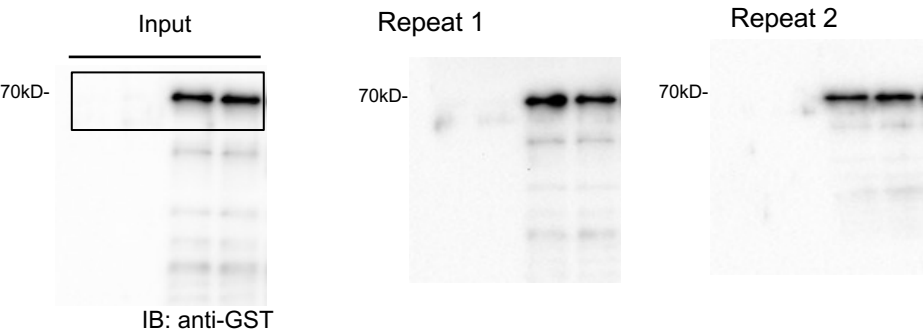

Supplement: Figure 4—figure supplement 3—source data 3. [file elife-82324-fig4-figsupp3-data3.zip › Figure 4-figure supplement 3-souce data 3/IB-data-Figure 4S3E.pdf]

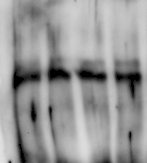

Supplement: Figure 4—figure supplement 3—source data 3. [file elife-82324-fig4-figsupp3-data3.zip › Figure 4-figure supplement 3-souce data 3/Figure 4S3E Repeat1/Input-ATRIP.tif]

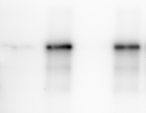

Supplement: Figure 4—figure supplement 3—source data 3. [file elife-82324-fig4-figsupp3-data3.zip › Figure 4-figure supplement 3-souce data 3/Figure 4S3E Repeat1/Bead-RPA.tif]

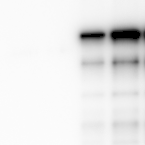

Supplement: Figure 4—figure supplement 3—source data 3. [file elife-82324-fig4-figsupp3-data3.zip › Figure 4-figure supplement 3-souce data 3/Figure 4S3E Repeat1/Bead-GST.tif]

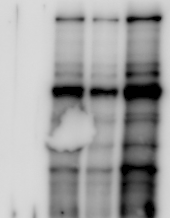

Supplement: Figure 4—figure supplement 3—source data 3. [file elife-82324-fig4-figsupp3-data3.zip › Figure 4-figure supplement 3-souce data 3/Figure 4S3E Repeat1/Bead-ATRIP.tif]

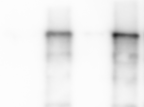

Supplement: Figure 4—figure supplement 3—source data 3. [file elife-82324-fig4-figsupp3-data3.zip › Figure 4-figure supplement 3-souce data 3/Figure 4S3E Repeat1/Input-RPA.tif]

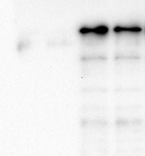

Supplement: Figure 4—figure supplement 3—source data 3. [file elife-82324-fig4-figsupp3-data3.zip › Figure 4-figure supplement 3-souce data 3/Figure 4S3E Repeat1/Input-GST.tif]

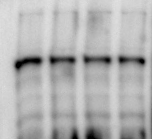

Supplement: Figure 4—figure supplement 3—source data 3. [file elife-82324-fig4-figsupp3-data3.zip › Figure 4-figure supplement 3-souce data 3/Figure 4S3E initial/Input-ATRIP.tif]

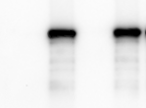

Supplement: Figure 4—figure supplement 3—source data 3. [file elife-82324-fig4-figsupp3-data3.zip › Figure 4-figure supplement 3-souce data 3/Figure 4S3E initial/Bead-RPA.tif]

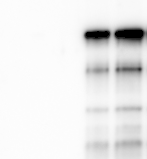

Supplement: Figure 4—figure supplement 3—source data 3. [file elife-82324-fig4-figsupp3-data3.zip › Figure 4-figure supplement 3-souce data 3/Figure 4S3E initial/Bead-GST.tif]

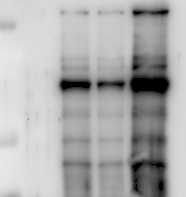

Supplement: Figure 4—figure supplement 3—source data 3. [file elife-82324-fig4-figsupp3-data3.zip › Figure 4-figure supplement 3-souce data 3/Figure 4S3E initial/Bead-ATRIP.tif]

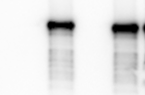

Supplement: Figure 4—figure supplement 3—source data 3. [file elife-82324-fig4-figsupp3-data3.zip › Figure 4-figure supplement 3-souce data 3/Figure 4S3E initial/Input-RPA.tif]

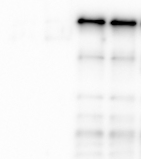

Supplement: Figure 4—figure supplement 3—source data 3. [file elife-82324-fig4-figsupp3-data3.zip › Figure 4-figure supplement 3-souce data 3/Figure 4S3E initial/Input-GST.tif]

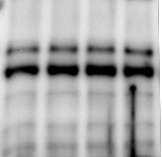

Supplement: Figure 4—figure supplement 3—source data 3. [file elife-82324-fig4-figsupp3-data3.zip › Figure 4-figure supplement 3-souce data 3/Figure 4S3E Repeat2/Input-ATRIP.tif]

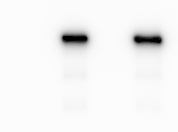

Supplement: Figure 4—figure supplement 3—source data 3. [file elife-82324-fig4-figsupp3-data3.zip › Figure 4-figure supplement 3-souce data 3/Figure 4S3E Repeat2/Bead-RPA.tif]

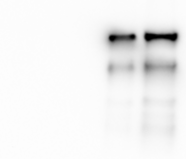

Supplement: Figure 4—figure supplement 3—source data 3. [file elife-82324-fig4-figsupp3-data3.zip › Figure 4-figure supplement 3-souce data 3/Figure 4S3E Repeat2/Bead-GST.tif]

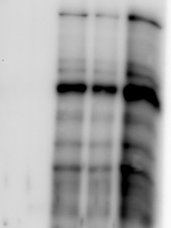

Supplement: Figure 4—figure supplement 3—source data 3. [file elife-82324-fig4-figsupp3-data3.zip › Figure 4-figure supplement 3-souce data 3/Figure 4S3E Repeat2/Bead-ATRIP.tif]

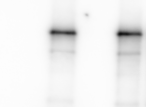

Supplement: Figure 4—figure supplement 3—source data 3. [file elife-82324-fig4-figsupp3-data3.zip › Figure 4-figure supplement 3-souce data 3/Figure 4S3E Repeat2/Input-RPA.tif]

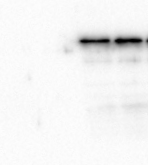

Supplement: Figure 4—figure supplement 3—source data 3. [file elife-82324-fig4-figsupp3-data3.zip › Figure 4-figure supplement 3-souce data 3/Figure 4S3E Repeat2/Input-GST.tif]
